# Directed Cyclic Graphs for Simultaneous Discovery of Time-Lagged and Instantaneous Causality from Longitudinal Data Using Instrumental Variables

**Published:** 2025

**Authors:** Wei Jin, Yang Ni, Amanda B. Spence, Leah H. Rubin, Yanxun Xu

**Affiliations:** Department of Applied Mathematics and Statistics, Johns Hopkins University, Baltimore, MD 21218, USA; Department of Statistics, Texas A&M University, College Station, TX 77843, USA; Department of Medicine, Georgetown University, Washington, DC 20007, USA; Departments of Neurology and Psychiatry, Johns Hopkins University School of Medicine, Baltimore, MD 21287, USA; Department of Applied Mathematics and Statistics, Johns Hopkins University, Baltimore, MD 21218, USA

**Keywords:** Bayesian structural learning, Causal discovery, Directed cyclic graph, Instrumental variable, Longitudinal cohort study

## Abstract

We consider the problem of causal discovery from longitudinal observational data. We develop a novel framework that simultaneously discovers the time-lagged causality and the possibly cyclic instantaneous causality. Under common causal discovery assumptions, combined with additional instrumental information typically available in longitudinal data, we prove the proposed model is generally identifiable. To the best of our knowledge, this is the first causal identification theory for directed graphs with general cyclic patterns that achieves unique causal identifiability. Structural learning is carried out in a fully Bayesian fashion. Through extensive simulations and an application to the Women’s Interagency HIV Study, we demonstrate the identifiability, utility, and superiority of the proposed model against state-of-the-art alternative methods.

## 1. Introduction

Causal discovery, which investigates the underlying causal relationships among a set of variables, has become increasingly important in statistics and machine learning and has found a broad range of applications in, e.g., bioinformatics (Hill et al., 2016), neuroscience (Shen et al., 2020), and atmospheric science (Runge et al., 2019a). While controlled experiments are the gold standard for establishing causality, they can be expensive, unethical, or even infeasible to implement, especially in scientific fields that involve human subjects. Therefore, many causal discovery methods aim to establish causality from observational data alone.

In this paper, we focus on causal discovery for longitudinal data, which naturally arise from many scientific disciplines. In our motivating application, people with HIV are recommended to follow up with their physicians semi-annually by current HIV guidelines (US Department of Health & Human Services, 2020) in order to collect their health information such as viral load, depressive symptoms, and kidney function longitudinally for better disease management. Among these health outcomes, some may cause others. For example, cognitive impairment may cause depression (Murata et al., 2000), and obesity may increase the risk for cardiovascular diseases (Zalesin et al., 2008). Learning causation instead of correlation among these comorbid conditions from such complex longitudinal data not only provides more accurate and robust predictions on future observations but also lays a foundation for downstream studies such as early intervention and therapeutic development.

Discovering causality from longitudinal/time-series data possesses its own advantages and challenges. On the one hand, the blessing of temporal priority (i.e., the cause always precedes its effects) breaks the symmetry in time, which can be used for orientating the causal relationship between two variables measured at different time points. On the other hand, the time gaps could be too large (say, months/years) for inferring causality that occurs at a faster rate (say, days/weeks). Although numerous methods have been developed for discovering causal structure from longitudinal/time-series data, such as the Granger causality model (Granger, 1969), the vector autoregressive model (Swanson and Granger, 1997), and the constraint-based approach (Runge et al., 2019b), the vast majority of them fail to account for instantaneous causality, which could be problematic if the gap between measuring times is large relatively to the rate of change from causal influence. In many real-world applications, detecting instantaneous causality is important. For example, the follow-up visits in our motivating HIV application are 6-months apart but the physiological and psychological changes can occur in a much shorter period of time, e.g., the improvement of people’s psychosocial functioning usually reduces subsequent depressive symptoms within a month (Dunn et al., 2012).

Causal discovery is commonly formalized as a structural learning task of a directed graph 𝒢=(𝒱,ℰ), which consists of a finite set of vertices 𝒱 representing the random variables of interest and a set of directed edges ℰ⊂𝒱×𝒱 representing the direct causal relationships (e.g., for any X,Y∈𝒱,(X→Y)∈ℰ indicates that X has a direct causal effect on Y) (Spirtes et al., 2000; Pearl, 2009). A fundamental challenge using this framework is to determine under what circumstances one can uniquely identify the graphical representation of the underlying causal mechanism from purely observational data. Existing works often rely on the assumption of acyclicity (Hyvärinen et al., 2010; Peters et al., 2013; Pamfil et al., 2020), i.e., there exist no feedback loops/directed cycles in the directed graph. For example, X⇄Y is not allowed. The assumption of acyclicity significantly simplifies both theoretical and computational analyses of directed graphs due to its convenient factorization, but many real-world causal relationships are cyclic/reciprocal. For instance, there may exist a cyclic causal relationship between viral load and depression for people with HIV: a high level of viral load is a crucial risk factor for developing depressive symptoms (Jain et al., 2021), while depressed people are more likely to engage in risk-taking behaviors, which may result in rapid HIV disease progression with a higher viral load (Brickman et al., 2017).

Despite the remarkable success of causal identification theories under the assumption of acyclicity (Shimizu et al., 2006; Hoyer et al., 2008a; Peters and Bühlmann, 2014), it remains an open question whether the unique causal identifiability for observational data alone can be achieved in general directed graphs that allow for cycles. It is well-known that a directed cyclic graph is generally only identifiable up to its Markov equivalence class (Spirtes, 1995; Koster, 1996; Lanne et al., 2017; Mooij and Claassen, 2020). The literature of unique causal identifiability is quite sparse. Lacerda et al. (2008) developed a linear additive model with non-Gaussian noises and provided a sufficient condition for its unique causal identifiability by assuming that the underlying directed graph only contains disjoint cycles, which may not hold and is hard to verify in practice. Mooij et al. (2011) proposed a non-linear additive model and proved its unique identifiability under the bivariate case. Hyttinen et al. (2012) proved the identifiability of a linear cyclic model using both observational and interventional data. In summary, existing work either makes the disjoint cycle assumption, requires interventions, or is restricted to the bivariate case.

In this paper, we develop a novel framework built upon directed cyclic graphs for discovering causal relationships from longitudinal data using instrumental variables. The key idea is to use time-lagged causes as instrumental variables for instantaneous causal discoveries. While instrumental variable approaches (Angrist et al., 1996) have been extensively used as powerful tools for inferring causal effects from observational data, our use of instrumental variables is to identify the causal structure without imposing the disjoint cycle assumption. By taking advantage of the identifiability results of independent component analysis (Comon, 1994), we prove the unique causal identifiability of the proposed model with a sufficient set of instrumental variables. For structural learning, we adopt a fully Bayesian approach through spike-and-slab priors for selecting a sparse set of causes, while adjusting for both time-varying and time-invariant covariates. Through extensive simulations and an application to a large-scale HIV longitudinal cohort study, i.e., the Women’s Interagency HIV Study (WIHS, Adimora et al. 2018), we demonstrate the identifiability, utility, and robustness of the proposed method, and also prove its advantages over state-of-the-art competitors. For reproducibility and broader dissemination, we make the R code that implements the proposed model publicly available at https://github.com/bluejw/BayesDCG.

In summary, our major contributions are two-fold. First, we propose a novel framework for longitudinal causal discovery that accounts for both time-lagged and possibly cyclic instantaneous causal relationships. Second, we establish the causal identifiability theory for directed graphs with general cyclic patterns by using the instrumental variable approach and taking advantage of the longitudinal nature of the data.

The rest of this paper proceeds as follows. In Section 2, we present the data-generating model of the proposed framework. In Section 3, we prove the unique causal identifiability of the proposed model under a general directed cyclic graph setup. In Section 4, we describe the Bayesian structural learning procedure for estimating the proposed model. In Section 5, through extensive simulation studies, we empirically verify our causal identification theory and evaluate the performance of the proposed model against state-of-the-art alternatives. In Section 6, we demonstrate the utility and superiority of the proposed model by applying it to a real-world large-scale HIV database. Lastly, we conclude with a discussion in Section 7.

## 2. Data-Generating Model

Let $Y_{ij}\in R^{Q}$ denote longitudinal health outcomes (e.g., viral load and depression score) for individual i at visit $j=1,\ldots ,J_{i}$, for which we aim to discover their causal relationships. Let $X_{ij}\in R^{S}$ denote a set of exogenous variables/covariates (e.g., age and race) for individual i at visit j, which can be either time-varying or time-invariant. The causal direction between any covariate and any health outcome is fixed to be from the former to the latter *a priori*. The causal relationships/dependencies among covariates are not of primary interest and thus will not be modeled in this work.

For notational simplicity, the individual index i will be suppressed when understood from the context. To take into account both the time-lagged and the instantaneous causalities, we propose the following data-generating model,

(1) $$Y_{j}=\mu +\sum_{\ell =0}^{L_{y}}B_{\ell }Y_{j-\ell }+\sum_{\ell =0}^{L_{x}}A_{\ell }X_{j-\ell }+E_{j},$$

where $L_{y}$ and $L_{x}$ are the numbers of time lags for longitudinal health outcomes and covariates, respectively, $B_{\ell }$ is a Q×Q matrix whose (q, p)-th element is $\beta_{\ell qp}$, $A_{\ell }$ is a Q×S matrix whose (q, s)-th element is $\alpha_{\ell qs}$, $\mu \in R^{Q}$ is the intercept, and $E_{j}\in R^{Q}$ is the exogenous error. The direct causes of $Y_{j}$ consist of both instantaneous direct causes (i.e., a subset of $Y_{j}$ and $X_{j}$) and time-lagged direct causes (i.e., a subset of $Y_{j-1},\ldots ,Y_{j-L_{y}}$, $X_{j-1},\ldots ,X_{j-L_{x}}$).

**Figure 1:**
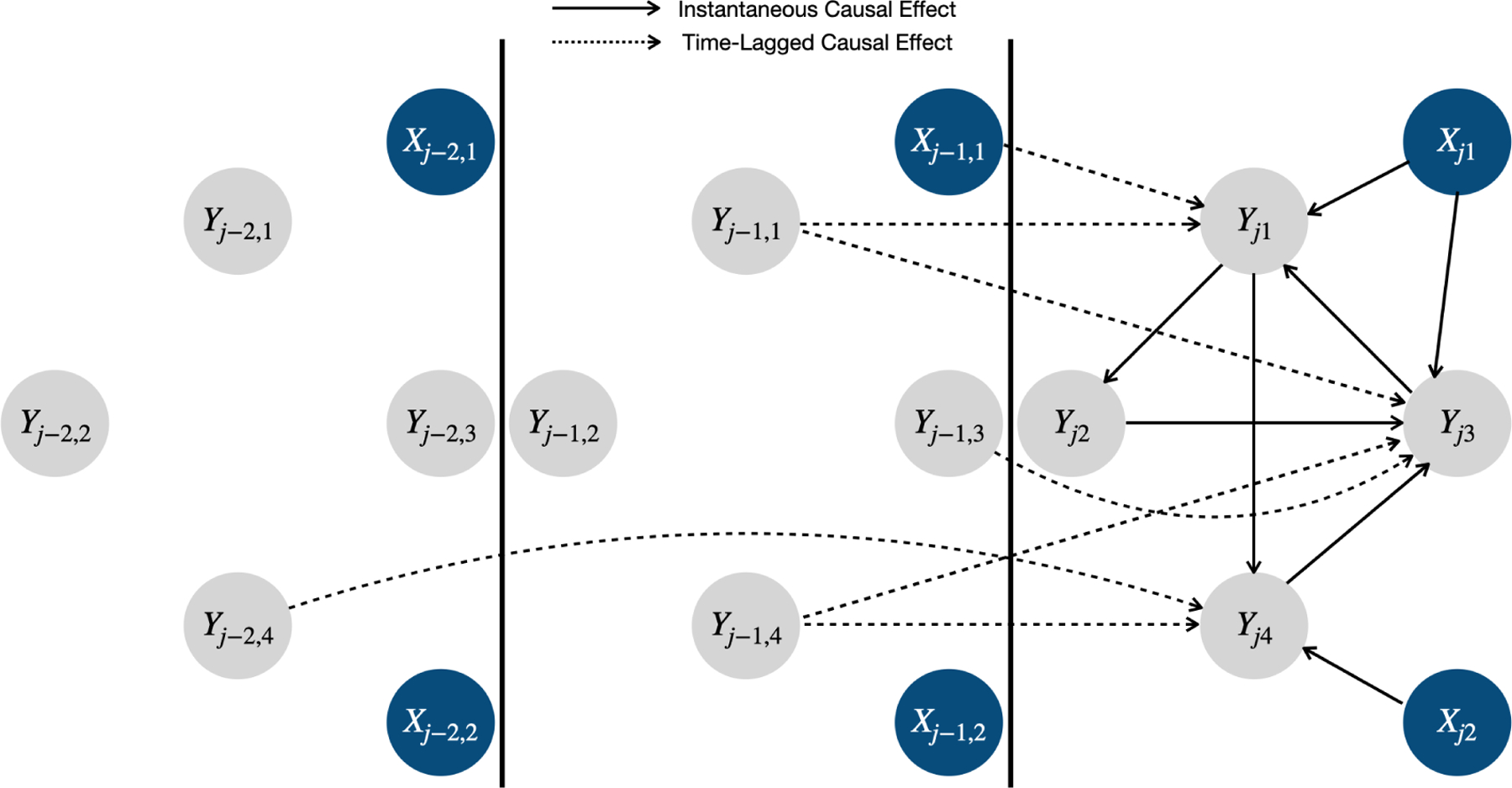
Graphical representation of the proposed model. The grey and blue circles represent the longitudinal health outcomes and covariates, respectively. The solid and dashed black lines indicate the instantaneous and time-lagged causal effects, respectively. Directed cycles are allowed for the instantaneous causality in the proposed model. The causal relationships among covariates are not accounted for in the proposed model. Note that due to the assumption of stationarity, this graphical representation applies to any time step j.

The proposed model is paired with a directed graph 𝒢=(𝒱,ℰ), which consists of a finite set of vertices $𝒱=\left(\cup_{j=1}^{J}{,}_{q=1}^{Q}Y_{jq}\right)\cup \left(\cup_{j=1}^{J}{,}_{s=1}^{S}X_{js}\right)$ and a set of directed edges ℰ⊂𝒱×𝒱. Under the causal Markov assumption (Richardson, 1996), i.e., the probability distribution respects the Markov property of the causal graph, 𝒢 has a causal interpretation. Specifically, there exists a causal relationship between two health outcomes $\left(Y_{jq}\leftarrow Y_{j^{\prime }p}\right)\in ℰ$ if $0\leq j-j^{\prime }\leq L_{y}$ and $\beta_{j-j^{\prime },qp}\neq 0$. Note that we assume $\beta_{0qq}=0$ for any q as we do not allow instantaneous *self-loops* (i.e., $Y_{jq}\leftarrow Y_{jq}$). For health outcomes and covariates, there exists a causal relationship between a health outcome and a covariate $\left(Y_{jq}\leftarrow X_{j^{\prime }s}\right)\in ℰ$ if $0\leq j-j^{\prime }\leq L_{x}$ and $\alpha_{j-j^{\prime },qs}\neq 0$. By definition, cycles are allowed for the instantaneous causality, e.g., if $\beta_{0qp}\neq 0$ and $\beta_{0pq}\neq 0,p\neq q$, then there is a cycle $Y_{jq}⇄Y_{jp}$ between $Y_{jq}$ and $Y_{jp}$. Figure 1 illustrates the graphical representation of the proposed model.

Due to the existence of cycles in the instantaneous causal effects $B_{0}$, the right-hand side of Equation (1) does not directly specify the conditional distribution of $Y_{j}$. However, we can derive this conditional distribution from the distribution of the errors $E_{j}$. Specifically, consider the mapping $f:Y_{j}\to E_{j}$, which has the form $\left(I-B_{0}\right)Y_{j}-\mu -\sum_{\ell =1}^{L_{y}}B_{\ell }Y_{j-\ell }-\sum_{\ell =0}^{L_{x}}A_{\ell }X_{j-\ell }=E_{j}$ induced by Equation (1). By the change-of-variable formula, we have

(2) $$p_{Y_{j}}\left(y_{j}\right)=p_{E_{j}}\left(f\left(y_{j}\right)\right)\left|\nabla f\left(y_{j}\right)\right|=p_{E_{j}}\left(y_{j}-B_{0}y_{j}-\mu -\sum_{\ell =1}^{L_{y}}B_{\ell }y_{j-\ell }-\sum_{\ell =0}^{L_{x}}A_{\ell }x_{j-\ell }\right)\left|I-B_{0}\right|,$$

where |⋅| denotes the absolute value of the determinant of a matrix.

For each time step j, Equation (1) can be written as $Y_{j}=B_{0}Y_{j}+C_{j}$, where $C_{j}$ includes all non-cyclic components (i.e., the intercept μ, the time-lagged effects of health outcomes $\sum_{\ell =1}^{L_{y}}B_{\ell }Y_{j-\ell }$, the covariate effects $\sum_{\ell =0}^{L_{x}}A_{\ell }X_{j-\ell }$, and the exogenous error $E_{j}$). To ensure that the proposed model is well-defined, we impose the following *stability* condition on $B_{0}$. Specifically, we assume that the maximum modulus of $B_{0}$’s eigenvalues is strictly less than 1, indicating that there exists a matrix norm ‖⋅‖ such that $\left\|B_{0}\right\|<1$ (Theorem 5.6.12 in Horn and Johnson (2012)). Consequently, the matrix $I-B_{0}$ is always invertible, ensuring that Equation (1) has a unique solution at each time step j, given by $Y_{j}={\left(I-B_{0}\right)}^{-1}C_{j}$. This is because if there exists a matrix norm ‖⋅‖ such that $\left\|B_{0}\right\|<1$, then the series $\sum_{n=0}^{\infty }B_{0}^{n}$ converges to some matrix. Since $\left(I-B_{0}\right)\sum_{n=0}^{N}B_{0}^{n}=I-B_{0}^{N+1}\to I$ as N→∞, we conclude that ${\left(I-B_{0}\right)}^{-1}=\sum_{n=0}^{\infty }B_{0}^{n}$, and hence $I-B_{0}$ is invertible (Corollary 5.6.16 in Horn and Johnson (2012)).

There are two special cases of the proposed model, i.e., the (cross-sectional) structural causal model (SCM, Bollen 1989) and the vector autoregressive model (VAR, Swanson and Granger 1997). In SCM, $L_{y}=L_{x}=0$ in Equation (1); and in VAR, $Y_{j}$ only appears on the left-hand side of Equation (1). The proposed model is advantageous over both of them. Compared to SCM, the proposed model, as we will show later, has stronger causal identification results by leveraging the longitudinal data. Compared to VAR, the proposed model can capture the instantaneous causality that occurs faster than the measuring gaps. Although there exist many causal discovery methods that also account for both time-lagged and instantaneous causal relationships (Entner and Hoyer, 2010; Kadowaki et al., 2013; Malinsky and Spirtes, 2018; Runge, 2020), they all rely on the assumption of acyclicity. While theoretically and computationally convenient, acyclic models fall short when attempting to represent the cyclic/reciprocal causal relationships, which are common in many real-world applications. In this work, we address this critical gap by offering a novel framework for longitudinal causal discovery, which not only establishes a theoretical guarantee of unique causal identifiability for directed graphs with general cyclic patterns but also provides a Bayesian structural learning algorithm that efficiently estimates the proposed model.

Here we briefly introduce the key ideas for proving the causal identifiability of the proposed model (i.e., Equation (1)), with more details to be discussed in the next section. We begin by establishing the causal identifiability theory for directed graphs with general cyclic patterns, leveraging the identifiability results of the independent component analysis (ICA, Comon 1994), and using instrumental variables. We then apply this causal identifiability theory to the causal graph ${𝒢}_{j}$ associated with the (cross-sectional) SCM, $Y_{j}=B_{0}Y_{j}+E_{j}$, defined by the instantaneous causal effects $B_{0}$ that potentially involve cycles among variables in $Y_{j}$ at each time step j of the proposed model. Lastly, we derive the causal identifiability of the proposed model from the causal identifiability of the instantaneous causal graph ${𝒢}_{j}$.

Specifically, we first follow the idea of Shimizu et al. (2006) and Lacerda et al. (2008) by making the following assumptions, which are common in the causal discovery literature.

**Assumption 1 (Causal Sufficiency)** *There are no unmeasured confounders (i.e., hidden common causes of two or more longitudinal health outcomes)*.

**Assumption 2 (Non-Gaussian Noise)** *The jointly independent exogenous errors are all continuous-valued random variables with non-Gaussian distributions*.

**Remark 1** *Causal sufficiency is assumed only for longitudinal health outcomes, while no assumptions are made regarding causal relationships/dependencies among covariates. The covariates in* Equation (1) *essentially serve the same role as secondary variables in a conditional directed acyclic graph* (Oates et al., 2016). *In other words, the proposed model is conditioned on the covariates*.

These two assumptions are critical to establishing our causal identifiability theory by leveraging the identifiability result of ICA. The identifiability theory of ICA defines an equivalence class that is much smaller and easier to handle compared to the Markov equivalence class of directed graphs with general cyclic patterns.

Next, to further determine a unique directed graph from the equivalence class defined by ICA, we use the instrumental variable approach (Angrist et al., 1996).

**Definition 2 (Instrumental Variable)** *For a variable* Y *in the directed graph* $𝒢,I_{Y}\notin 𝒢$ *is an instrumental variable for* Y *if* $I_{Y}\to Y$ *is the only directed edge involving both* $I_{Y}$ *and any variables in* 𝒢.

**Remark 3** *Note that the above definition slightly differs from the original concept of an instrumental variable. In our definition, we assume the absence of unmeasured confounders (i.e., Assumption 1), whereas the traditional instrumental variable is required to be independent of any unmeasured confounders*.

**Remark 4** *Note that instrumental variables are known to be parents, but not children, of the variables in the directed graph* 𝒢,* and they are not in* 𝒢.* For example, at each time step* j *of the proposed model* (1), *the time-lagged variables* $Y_{j-\ell },1\leq \ell \leq L_{y}$ *and the covariates* $X_{j-\ell },0\leq \ell \leq L_{x}$ *can serve as the potential instrumental variables for* $Y_{j}\in {𝒢}_{j}$.

**Remark 5** *There are no constraints on the causal relationships/dependencies among instrumental variables. This is because, as we will see later, the causal relationships/dependencies among instrumental variables do not impact our causal identification results*.

The instrumental variable approach is a powerful tool commonly used in causal inference literature to identify causal effects under unmeasured confounding. However, its application in identifying causal structures using SCMs remains relatively limited in the literature. Both Oates et al. (2016) and Chen et al. (2023) developed methods for estimating cross-sectional SCMs using instrumental variables. Thams et al. (2024) proposed a framework for learning underlying causal structures among multivariate time series using instrumental time series. However, all these approaches were restricted to directed acyclic graphs. A recent work (Li et al., 2024) proposed to discover the causal direction in bivariate graphs using instrumental variables, allowing for both cycles and unmeasured confounders, but it remains unclear how to extend the approach to handle multivariate graphs.

In this work, we propose to use the instrumental variable approach to identify a unique directed graph within the equivalence class defined by ICA. In particular, each instrumental variable may have different children across different graphs in the equivalence class, enabling the separation of certain causal graphs from others. When a sufficient set of instrumental variables is available, unique identifiability of the causal graph can be achieved.

## 3. Causal Identification Theory

In this section, under common causal discovery assumptions, combined with additional instrumental information typically available in longitudinal data, we establish the first causal identification theory for directed graphs with general cyclic patterns that achieves unique causal identifiability. The main idea is to find a sufficient set of instrumental variables that guarantee the causal identifiability, by taking advantage of the longitudinal data.

We begin with a brief introduction to structural causal models, directed cyclic graphs, and the independent component analysis, which are important ingredients for establishing our causal identification theory. Then we present our causal identification results. Lastly, we prove the unique causal identifiability of the proposed model (1) by applying our causal identification theory.

### 3.1 Preliminaries

Let $Y=\left(Y_{1},\ldots ,Y_{Q}\right)$ denote a number of Q observed variables. A structural causal model (SCM, Bollen 1989; Pearl 2009) consists of Q structural equations,

(3) $$Y_{q}=f_{q}\left(\text{pa}\left(Y_{q}\right),e_{q}\right),\;q=1,\ldots ,Q,$$

where $\text{pa}\left(Y_{q}\right)\subseteq \left\{Y_{1},\ldots ,Y_{Q}\right\}\setminus Y_{q}$ is the set of parents (i.e., direct causes) of $Y_{q},f_{q}(\cdot )$ is the structural causal function determining the value of the effect $Y_{q}$ in terms of its direct causes $\text{pa}\left(Y_{q}\right)$ and an exogenous error $e_{q}$. For simplicity and interpretability, we assume $f_{q}(\cdot )$ to be linear in this work. Then the above Q structural equations can be written in the following equivalent matrix form,

(4) Y=BY+E,

where B denotes the Q×Q linear coefficient matrix whose (q, p)-th element is $\beta_{qp}$, and $E=\left(e_{1},\ldots ,e_{Q}\right)$ denotes the errors. If the maximum modulus of B’s eigenvalues is strictly less than 1, then the SCM (4) is stable. The SCM (4) is associated with a directed graph 𝒢=(𝒱,ℰ) that represents its causal structure, where $Y_{q}\in 𝒱,q=1,\ldots ,Q$, and $\left(Y_{q}\leftarrow Y_{p}\right)\in ℰ$ if and only if $\beta_{qp}\neq 0$. Note that directed cycles are allowed in 𝒢.

**Definition 6 (Directed Cycle)** *A (directed) cycle, denoted by* 𝒪, *consists of a sequence of vertices* $\left(Y_{1},\ldots ,Y_{M}\right)$ *along with exactly* M≥2 *directed edges* $\left(Y_{1}\to Y_{2}\right)\in ℰ,\ldots ,\left(Y_{M-1}\to Y_{M}\right)\in ℰ,\left(Y_{M}\to Y_{1}\right)\in ℰ$.

**Figure 2:**
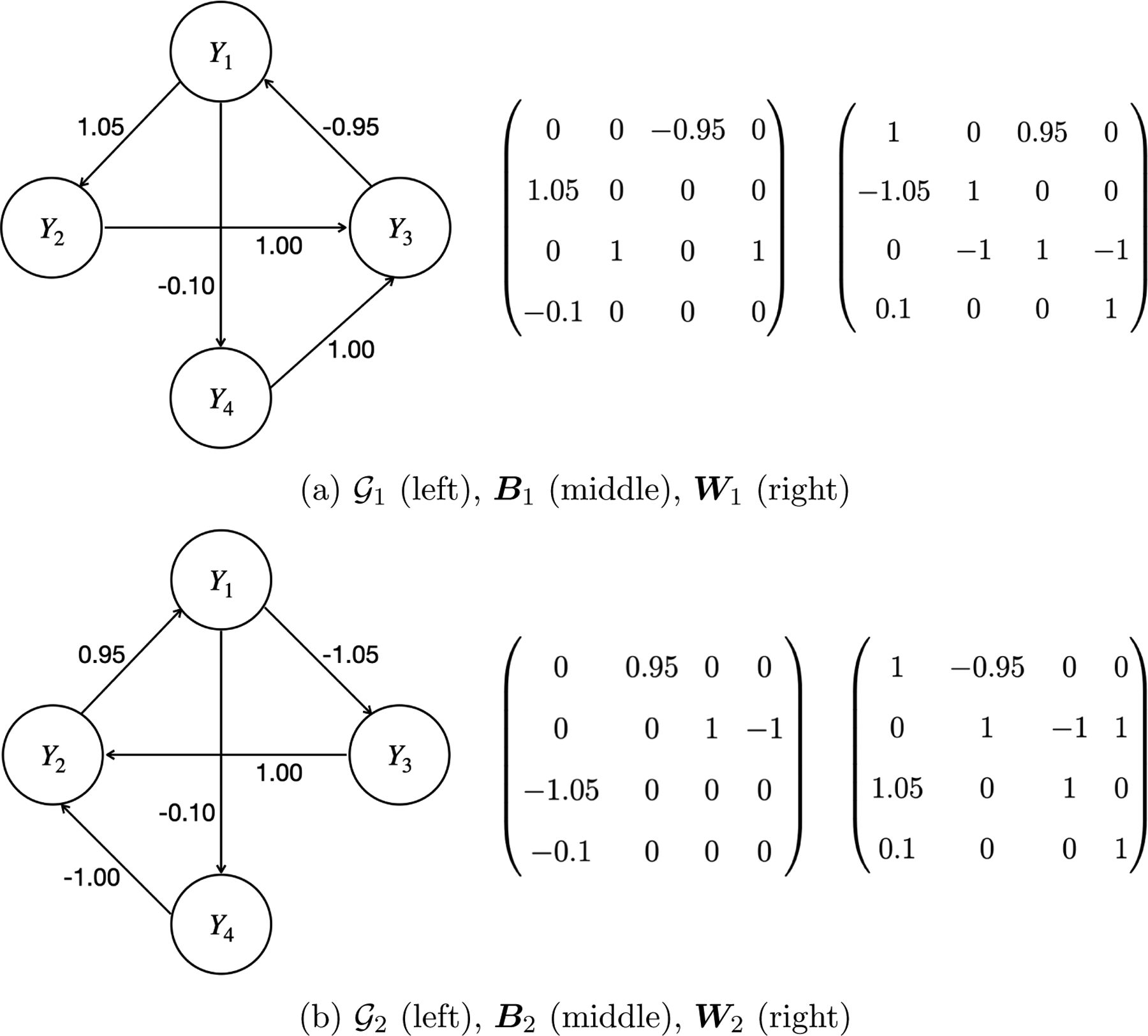
Two directed graphs ${𝒢}_{1}$ and ${𝒢}_{2}$ (both contain joint cycles) with their corresponding linear coefficient matrices $B_{1}$ and $B_{2}$, and unmixing matrices $W_{1}$ and $W_{2}$. ${𝒢}_{1}$ and ${𝒢}_{2}$ are in the same ICA equivalence class. In ${𝒢}_{2}$, the values 0.95 and −1.05 are approximations of 1/1.05 and −1/0.95, respectively.

**Figure S1:**
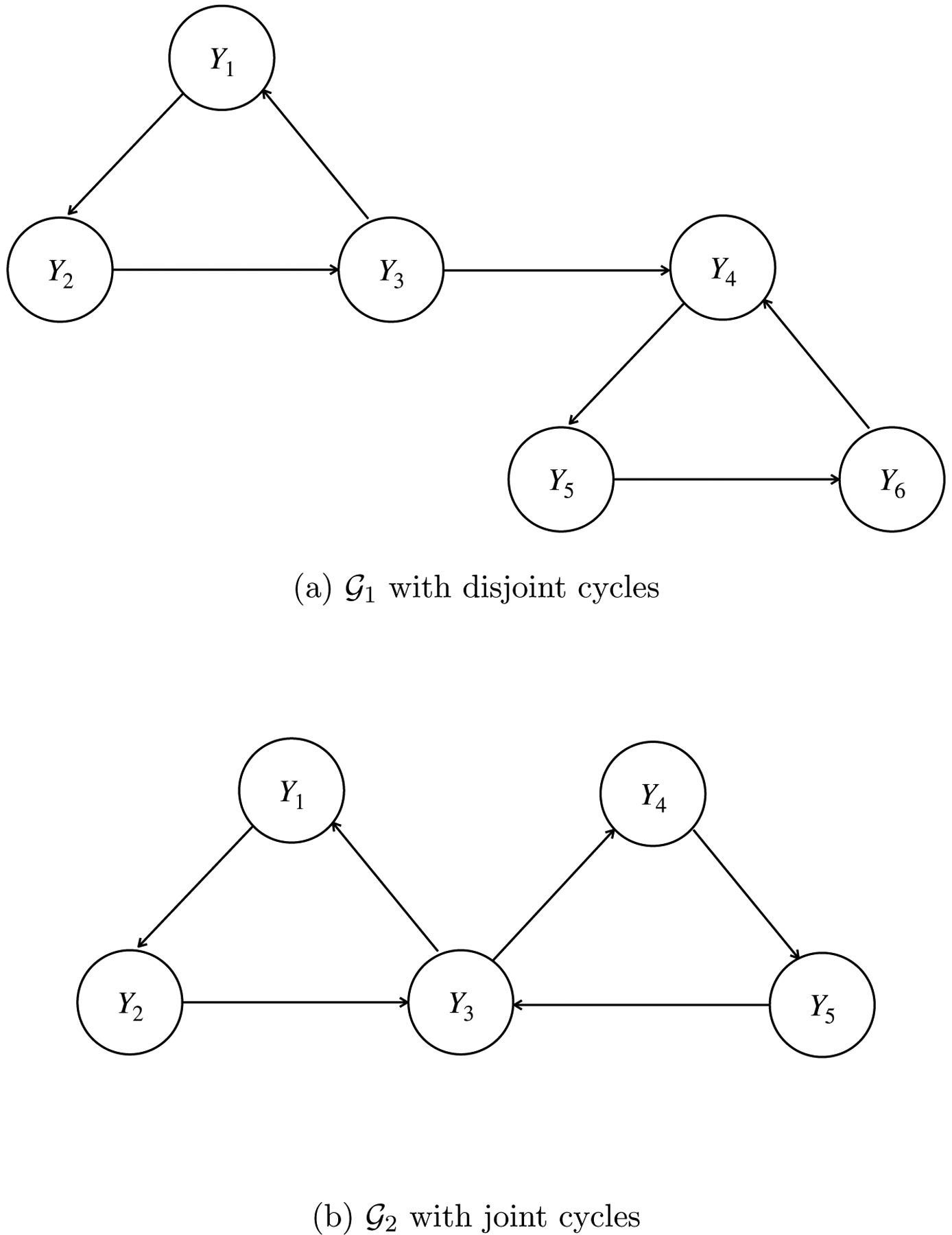
Directed graphs that contain disjoint cycles (top) and joint cycles (bottom). In ${𝒢}_{1}$, ${𝒪}_{1}:Y_{1}\to Y_{2}\to Y_{3}\to Y_{1}$ and ${𝒪}_{2}:Y_{4}\to Y_{5}\to Y_{6}\to Y_{4}$, where ${𝒪}_{1}\cap {𝒪}_{2}=\emptyset$. In ${𝒢}_{2}$, ${𝒪}_{3}:Y_{1}\to Y_{2}\to Y_{3}\to Y_{1}$ and ${𝒪}_{4}:Y_{3}\to Y_{4}\to Y_{5}\to Y_{3}$, where ${𝒪}_{3}\cap {𝒪}_{4}=\left\{Y_{3}\right\}$.

We assume that the number of vertices M≥2 as we exclude self-loops (e.g., $Y_{1}\to Y_{1}$). For example, the sequence of vertices ($Y_{1}$, $Y_{2}$, $Y_{3}$) forms a cycle if $Y_{1}\to Y_{2}$, $Y_{2}\to Y_{3}$, and $Y_{3}\to Y_{1}$; while it does not form a cycle if (i) $Y_{1}\to Y_{2}$, $Y_{2}\to Y_{3}$, and $Y_{1}\to Y_{3}$, or (ii) $Y_{1}\to Y_{2}$, $Y_{2}\to Y_{3}$, $Y_{3}\to Y_{1}$, and $Y_{1}\to Y_{3}$. Definition 6 is general for defining a directed cycle, which is also consistent with the definitions introduced by Spirtes (1995) and Koster (1996). By definition, for any variable Y in a cycle 𝒪, its parent in 𝒪 denoted by ${\text{pa}}_{𝒪}(Y)$ is unique. In addition, with a slight abuse of notation, we define the intersection of two cycles to be the vertices that are common to both cycles. Then two cycles ${𝒪}_{1}$ and ${𝒪}_{2}$ are *disjoint* if ${𝒪}_{1}\cap {𝒪}_{2}=\emptyset$, i.e., they donť share any vertices. Figure 2(a,b) illustrate two examples of directed graphs with joint cycles. Specifically, in ${𝒢}_{1}$, two cycles $Y_{1}\to Y_{2}\to Y_{3}\to Y_{1}$ and $Y_{1}\to Y_{4}\to Y_{3}\to Y_{1}$ intersect at $\left\{Y_{1},Y_{3}\right\};$ in ${𝒢}_{2}$, two cycles $Y_{1}\to Y_{3}\to Y_{2}\to Y_{1}$ and $Y_{1}\to Y_{4}\to Y_{2}\to Y_{1}$ intersect at $\left\{Y_{1},Y_{2}\right\}$. More examples of joint/disjoint cycles can be found in Appendix Figure S1.

An SCM associated with a directed cyclic graph is generally only identifiable up to its Markov equivalence class (Spirtes, 1995; Koster, 1996). To achieve unique causal identifiability, we adopt standard causal discovery assumptions (i.e., Assumptions 1 and 2 introduced in Section 2), which are essential for establishing our causal identification theory by leveraging the identifiability result of ICA. Specifically, the goal of the ICA is to obtain a unique *unmixing* matrix W=I-B in Equation (4) (so that Y can be uniquely expressed as a linear combination of the errors, i.e., $Y=W^{-1}E$, provided W is invertible), which is equivalent to a unique graphical representation 𝒢. Under Assumptions 1 and 2, the solution of ICA is guaranteed to be identifiable up to row-permuted row-scaled versions of W, which define an equivalence class of 𝒢. Note that if there are no self-loops in 𝒢 (e.g., a directed edge $\left(Y_{q}\leftarrow Y_{q}\right)\in ℰ$), then all the diagonal elements of B are zero (i.e., $\beta_{qq}=0,q=1,\ldots ,Q$). This implies that all the diagonal elements of W are non-zero (up to a scaling factor which can be determined by normalizing the rows of W such that all diagonal elements equal to one). Therefore, to avoid self-loops in 𝒢, we only consider row-permutations that are admissible, the definition of which is provided below.

**Definition 7 (Row-Permutation)** *A row-permutation* ϕ *(applied to a subset of the rows of an unmixing matrix W) is a bijective mapping from* R *to* R, *where* $R=\left\{r_{1},\ldots ,r_{K}\right\}\subseteq \{1,\ldots ,Q\},K\geq 2$*, such that* $\phi \left(r_{k}\right)\neq r_{k}$ *for* k=1,…,K.

**Definition 8 (Admissible Row-Permutation)** *A row-permutation* ϕ *(applied to an unmixing matrix) is said to be admissible if all the diagonal elements of the resulting unmixing matrix are non-zero*.

In summary, the *ICA equivalence class* consists of all directed graphs 𝒢 whose unmixing matrices W’s are admissible row-permutations of each other. For example, Figure 2 displays two directed graphs ${𝒢}_{1}$ and ${𝒢}_{2}$ with their corresponding unmixing matrices $W_{1}$ and $W_{2}$. Note that $W_{2}$ can be obtained by applying the admissible row-permutation ϕ:{1,2,3}→{1,2,3} such that ϕ(1)=3, ϕ(2)=1, ϕ(3)=2 to $W_{1}$, and then normalizing each row by dividing its diagonal element. Therefore, ${𝒢}_{1}$ and ${𝒢}_{2}$ are in the same ICA equivalence class.

Using this theoretical framework, Shimizu et al. (2006) proved that there exists exactly one directed acyclic graph in its ICA equivalence class. Lacerda et al. (2008) further proved that there exists exactly one directed graph associated with a stable SCM among all directed graphs that only contain disjoint cycles. However, the theory for unique causal identifiability still remains open for directed graphs that possibly contain joint cycles, since it is easy to find examples such that there are multiple directed graphs with joint cycles in the same ICA equivalence class, each of which corresponds to a stable SCM. For example, ${𝒢}_{1}$ and ${𝒢}_{2}$ in Figure 2 are in the same ICA equivalence class, and both of them are associated with stable SCMs (since the modulus of all eigenvalues of $B_{1}$ and $B_{2}$ are strictly less than 1). Therefore, ${𝒢}_{1}$ and ${𝒢}_{2}$ are not identifiable from observational data, although their graphical representations and causal interpretations are quite different. To the best of our knowledge, there are no existing unique causal identification results that consider such a setup where directed graphs may contain joint cycles. In the rest of this section, we will fill the gap and all detailed proofs are provided in Appendix A.

### 3.2 Main Results

We now summarize our main causal identification result for SCMs under the general directed cyclic graph setup using instrumental variables in Theorem 9.

**Theorem 9 (Causal Identification)** *Suppose that Assumptions 1–2 hold, then a directed graph* 𝒢 *with* N *directed cycles* ${𝒪}_{1},\ldots ,{𝒪}_{N}$ *(possibly joint with each other) can be uniquely identified in its ICA equivalence class if there are* N *(not necessarily distinct) variables $Y_{1}\in {𝒪}_{1},\ldots ,Y_{N}\in {𝒪}_{N}$*, *each of which has its own instrumental variable $I_{Y_{n}}$*, *for* n=1,…,N.

**Figure 3:**
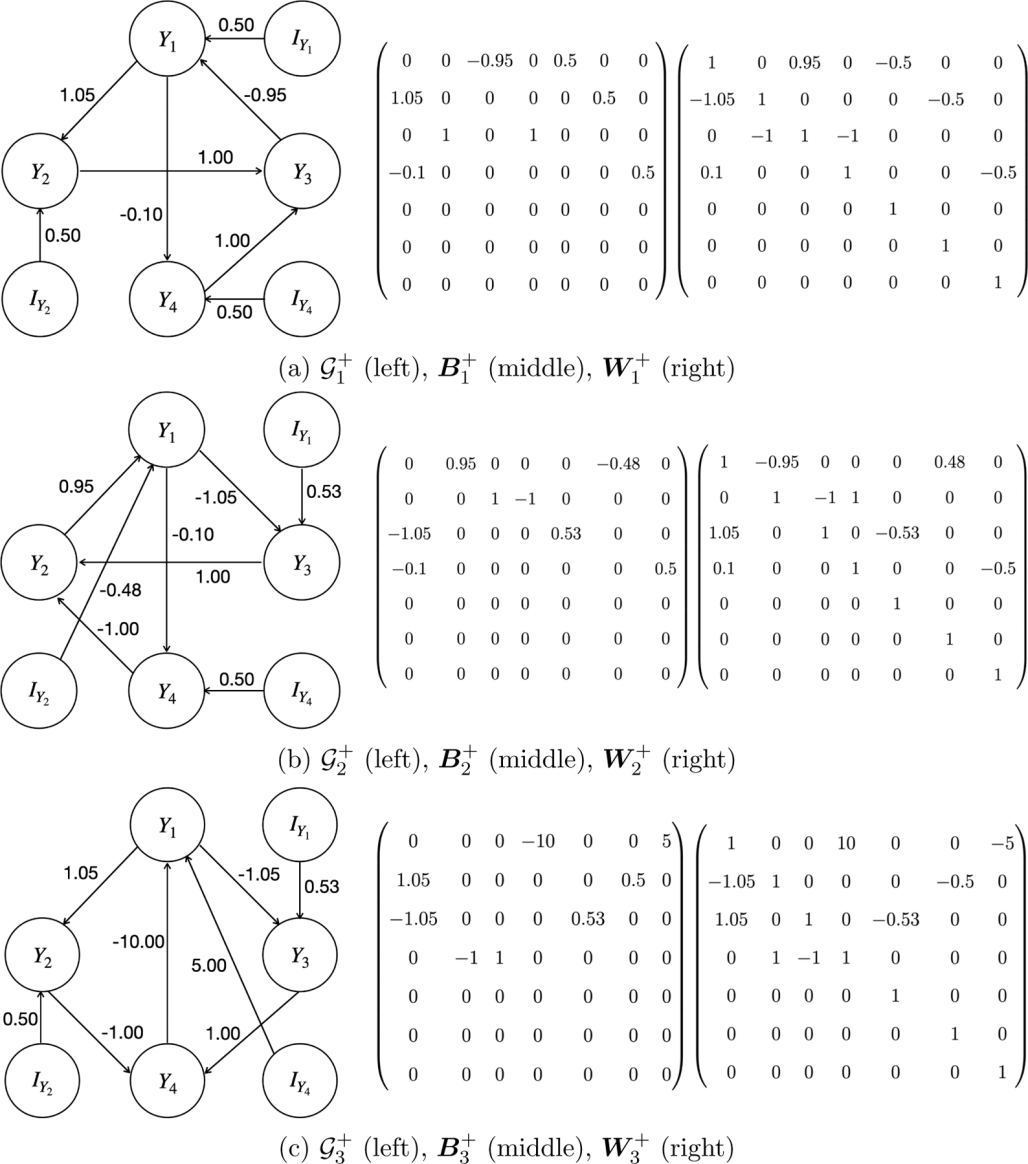
The ICA equivalence class of ${𝒢}_{1}^{+}:\left\{{𝒢}_{1}^{+},{𝒢}_{2}^{+},{𝒢}_{3}^{+}\right\}$, with their corresponding linear coefficient matrices $B_{1}^{+}$, $B_{2}^{+}$, and $B_{3}^{+}$, and unmixing matrices $W_{1}^{+}$, $W_{2}^{+}$, and $W_{3}^{+}$. The last three rows and columns in both the linear coefficient matrices and the unmixing matrices correspond to the instrumental variables $I_{Y_{1}}$, $I_{Y_{2}}$, and $I_{Y_{4}}$. In ${𝒢}_{2}^{+}$ and ${𝒢}_{3}^{+}$, the values 0.95, −1.05, 0.53, −0.48 are approximations of 1/1.05, −1/0.95, 0.5/0.95, −0.5/1.05, respectively.

Theorem 9 entails that the unique causal identifiability of any directed cyclic graph is guaranteed as long as each directed cycle within the graph consists of a variable that has its own instrumental variable. For example, ${𝒢}_{1}$ in Figure 2 consists of two directed cycles ${𝒪}_{1}:Y_{1}\to Y_{2}\to Y_{3}\to Y_{1}$ and ${𝒪}_{2}:Y_{1}\to Y_{4}\to Y_{3}\to Y_{1}$, which are joint with each other. Suppose that $I_{Y_{1}}$, $I_{Y_{2}}$, and $I_{Y_{4}}$ are the instrumental variables for $Y_{1}$, $Y_{2}$, and $Y_{4}$, respectively. Let ${𝒢}_{1}^{+}$ denote the directed graph formed by incorporating these instrumental variables into ${𝒢}_{1}$ (i.e., Figure 3(a)). By Theorem 9, there are two ways to achieve the unique identification of ${𝒢}_{1}^{+}$ within its ICA equivalence class $\left\{{𝒢}_{1}^{+},{𝒢}_{2}^{+},{𝒢}_{3}^{+}\right\}$ (shown in Figure 3). First, we can uniquely identify ${𝒢}_{1}^{+}$ by utilizing the instrumental variable $I_{Y_{1}}$ for $Y_{1}\in {𝒪}_{1}\cap {𝒪}_{2}$. This is because $Y_{1}$ is not the child of $I_{Y_{1}}$ in both ${𝒢}_{2}^{+}$ and ${𝒢}_{3}^{+}$. Second, since $Y_{2}\in {𝒪}_{1}$ is not the child of $I_{Y_{2}}$ in ${𝒢}_{2}^{+}$ and $Y_{4}\in {𝒪}_{2}$ is not the child of $I_{Y_{4}}$ in ${𝒢}_{3}^{+}$, ${𝒢}_{1}^{+}$ can also be uniquely identified with the help of the collection of instrumental variables $\left\{I_{Y_{2}},I_{Y_{4}}\right\}$. However, either $I_{Y_{2}}$ or $I_{Y_{4}}$ alone is not sufficient for the unique identification of ${𝒢}_{1}^{+}$. Note that the unique identification of ${𝒢}_{1}$ is then derived from the unique identification of ${𝒢}_{1}^{+}$.

**Remark 10** *To achieve unique causal identification, as stated in Theorem 9, prior knowledge of instrumental variables is essential. This information allows us to exclude certain members from the ICA equivalence class, which is a critical step that goes beyond the existing causal identification result in* Lacerda et al. (2008). *For instance, consider the ICA equivalence class shown in* Figure 3, *denoted as* $\left\{{𝒢}_{1}^{+},{𝒢}_{2}^{+},{𝒢}_{3}^{+}\right\}$. *If we know that* $I_{Y_{1}}$ *is an instrumental variable for* $Y_{1}$, *we can uniquely identify* ${𝒢}_{1}^{+}$. *In contrast, if we ascertain that $I_{Y_{1}}$ is an instrumental variable for* $Y_{3}$, *we can exclude* ${𝒢}_{1}^{+}$ *from consideration, narrowing down the ICA equivalence class to* $\left\{{𝒢}_{2}^{+},{𝒢}_{3}^{+}\right\}$. *In summary, the key point to uniquely identifying a directed graph from its ICA equivalence class lies in determining a sufficient set of instrumental variables based on prior knowledge*.

**Remark 11** *Theorem 9 provides a sufficient condition for unique causal identifiability, which may require a large number of instrumental variables if there are many directed cycles. However, even with an insufficient number of instrumental variables, we can still reduce the number of equivalent graphs. For example, consider the ICA equivalence class shown in* Figure 3, *denoted as* $\left\{{𝒢}_{1}^{+},{𝒢}_{2}^{+},{𝒢}_{3}^{+}\right\}$. *Suppose we only know that* $I_{Y_{2}}$ *is an instrumental variable for* $Y_{2}$. *Although this information is insufficient for achieving unique causal identifiability, we can exclude* ${𝒢}_{2}^{+}$ *from consideration, thereby narrowing the ICA equivalence class to* $\left\{{𝒢}_{1}^{+},{𝒢}_{3}^{+}\right\}$. *Similarly, if we only know that* $I_{Y_{4}}$ *is an instrumental variable for* $Y_{4}$, *we can exclude* ${𝒢}_{3}^{+}$ *from consideration, thereby reducing the ICA equivalence class to* $\left\{{𝒢}_{1}^{+},{𝒢}_{2}^{+}\right\}$.

**Remark 12** *It is possible to reduce the number of instrumental variables required for unique identification in Theorem 9 by leveraging the stability condition in certain cases. For example, since the SCM associated with* ${𝒢}_{3}^{+}$ *in* Figure 3 *is unstable, we can uniquely identify* ${𝒢}_{1}^{+}$ *from its ICA equivalence class by only using* $Y_{2}$’*s instrumental variable* $I_{Y_{2}}$, *which is impossible without the stability condition. In contrast, even with the stability condition, we are still not able to uniquely identify* ${𝒢}_{1}^{+}$ *by only using the instrumental variable* $I_{Y_{4}}$ *for* $Y_{4}$.

We now outline our identification strategy leading to Theorem 9. First, we introduce the definition of *irreducible* row-permutations, which plays a central role in the proof. Then we summarize two key ingredients in the proof of the identification theory, both built upon irreducible row-permutations, in Lemma 14.

**Definition 13 (Irreducible Row-Permutation)** *A row-permutation* ϕ:R→R *is said to be irreducible if there does not exist another row-permutation* ψ:T→T,T⊂R, *such that* ψ(T)=ϕ(T)*, where* ϕ(T) *is the image of the map *ϕ *restricted to domain *T.

For example, the row-permutation ϕ:{1,2,3}→{1,2,3}, where ϕ(1)=3, ϕ(2)=1, and ϕ(3)=2, is irreducible. However, the row-permutation ϕ:{1,2,3,4,5}→{1,2,3,4,5}, where ϕ(1)=3, ϕ(2)=4, ϕ(3)=5, ϕ(4)=2, and ϕ(5)=1, is not irreducible, since there exist two other row-permutations $\psi_{1}:\{1,3,5\}\to \{1,3,5\}$, where $\psi_{1}(1)=3$, $\psi_{1}(3)=5$, and $\psi_{1}(5)=1$, and $\psi_{2}:\{2,4\}\to \{2,4\}$, where $\psi_{2}(2)=4$ and $\psi_{2}(4)=2$.

**Lemma 14** *If an irreducible row-permutation *$\phi :R\to R,R=\left\{r_{1},\ldots ,r_{K}\right\}$, *applied to the unmixing matrix *W *associated with a directed graph* 𝒢 *is admissible, then*

- *there is a directed cycle* 𝒪 *in* 𝒢 *formed by* $\left\{Y_{r_{1}},\ldots ,Y_{r_{K}}\right\}$*, and* ϕ *reverses the direction of* 𝒪. *Without loss of generality, assume that* $𝒪:Y_{r_{1}}\leftarrow Y_{r_{2}}\leftarrow \cdots \leftarrow Y_{r_{K}}\leftarrow Y_{r_{1}}$*, then in the directed graph* ${𝒢}^{\prime }$ *associated with the resulting unmixing matrix* $W^{\prime }$, *we have* ${𝒪}^{\prime }:Y_{r_{1}}\to Y_{r_{2}}\to \cdots \to Y_{r_{K}}\to Y_{r_{1}}$;
- ϕ *changes the edge* $pa_{𝒢\setminus 𝒪}\left(Y_{r_{k}}\right)\to Y_{r_{k}}$ *in* 𝒢 *to the edge* $pa_{𝒢\setminus 𝒪}\left(Y_{r_{k}}\right)\to pa_{𝒪}\left(Y_{r_{k}}\right)$ *in* ${𝒢}^{\prime }$*, for* k=1,…,K, *where* $pa_{𝒪}\left(Y_{r_{k}}\right)$ *denotes the unique parent of* $Y_{r_{k}}$ *that lies inside the directed cycle* 𝒪, *and* $pa_{𝒢\setminus 𝒪}\left(Y_{r_{k}}\right)=\left\{Y\in 𝒱|\left(Y\to Y_{r_{k}}\right)\in ℰ\setminus {ℰ}_{𝒪}\right\}$ *where* ${ℰ}_{𝒪}$ *is the set of edges in* 𝒪, i.e., $pa_{𝒢\setminus 𝒪}\left(Y_{r_{k}}\right)$ *denotes all parents of* $Y_{r_{k}}$ *such that the edge* $pa_{𝒢\setminus 𝒪}\left(Y_{r_{k}}\right)\to Y_{r_{k}}$ *lies outside the directed cycle* 𝒪.

**Remark 15** *Note that $pa_{𝒢\setminus 𝒪}\left(Y_{r_{k}}\right)$ defined in Lemma 14(ii) may be empty and if not, there may be more than one such parent. For example,* ${𝒢}_{2}$ *in* Figure 2 *can be obtained by reversing* ${𝒪}_{1}:Y_{1}\to Y_{2}\to Y_{3}\to Y_{1}$ *in* ${𝒢}_{1}$*, and then changing the edge* $Y_{4}\to Y_{3}$ *to* $Y_{4}\to Y_{2}$. *In this case, we have* $pa_{{𝒢}_{1}\setminus {𝒪}_{1}}\left(Y_{3}\right)=\left\{Y_{4}\right\}$, *whereas ${pa}_{{𝒢}_{1}\setminus {𝒪}_{1}}\left(Y_{1}\right)=pa_{{𝒢}_{1}\setminus {𝒪}_{1}}\left(Y_{2}\right)=\emptyset$*. *Now suppose that we add an instrumental variable* $I_{Y_{3}}$ *for* $Y_{3}$ *in* ${𝒢}_{1}$, *then* $pa_{{𝒢}_{1}\setminus {𝒪}_{1}}\left(Y_{3}\right)=\left\{Y_{4},I_{Y_{3}}\right\}$. *Additionally, the definition of* $pa_{𝒢\setminus 𝒪}\left(Y_{r_{k}}\right)$ *only requires that the edge* $pa_{𝒢\setminus 𝒪}\left(Y_{r_{k}}\right)\to Y_{r_{k}}$ *lies outside the directed cycle* 𝒪, *meaning that the vertices* $pa_{𝒢\setminus 𝒪}\left(Y_{r_{k}}\right)$ *themselves can be either inside or outside the cycle. For example, in* Figure 2 *we have* ${pa}_{{𝒢}_{1}\setminus {𝒪}_{1}}\left(Y_{3}\right)=\left\{Y_{4}\right\}$, *where vertex* $Y_{4}$ *lies outside directed cycle* ${𝒪}_{1}$. *Now suppose that we add an edge* $Y_{1}\to Y_{3}$ *in* ${𝒢}_{1}$, *then we have* $pa_{{𝒢}_{1}\setminus {𝒪}_{1}}\left(Y_{3}\right)=\left\{Y_{1},Y_{4}\right\}$ *since edge* $Y_{1}\to Y_{3}$ *lies outside* ${𝒪}_{1}$, *even though vertex* $Y_{1}$ *itself lies inside* ${𝒪}_{1}$.

Lemma 14 indicates that applying an admissible irreducible row-permutation to the unmixing matrix is equivalent to performing the following two steps to the corresponding directed graph 𝒢: (i) reversing a directed cycle $𝒪:\left\{Y_{r_{1}},\ldots ,Y_{r_{K}}\right\}$ in 𝒢 and (ii) changing the child of ${\text{pa}}_{𝒢\setminus 𝒪}\left(Y_{r_{k}}\right)$ from $Y_{r_{k}}$ to ${\text{pa}}_{𝒪}\left(Y_{r_{k}}\right)$. Note that the proof of Theorem 4 in Lacerda et al. (2008) employed an argument that is identical to our Lemma 14(i). This argument leads to the unique identification of any directed graph that only consists of disjoint cycles under the stability condition. However, this argument alone is insufficient to uniquely identify a directed graph that possibly contains joint cycles, even when considering the stability condition. In fact, this is precisely the theoretical gap that we aim to address in this work.

Next, we will show in Proposition 16 that any admissible row-permutation can be decomposed into a collection of disjoint admissible irreducible row-permutations.

**Proposition 16** *For any admissible row-permutation* ϕ:R→R, *there exists a collection of* D≥1 *admissible irreducible row-permutations ${\left\{\phi_{d}:R_{d}\to R_{d}\right\}}_{d=1}^{D}$, where* $\cup_{d=1}^{D}R_{d}=R,R_{d}\cap R_{d^{\prime }}=\emptyset$ *for* $1\leq d\neq d^{\prime }\leq D$, *such that applying* ϕ *or sequentially applying $\phi_{1},\ldots ,\phi_{D}$ to any unmixing matrix yields the same row-permuted unmixing matrix*.

Therefore, by Proposition 16, we can generalize the results from Lemma 14 regarding admissible irreducible row-permutations to any admissible row-permutations. This extension leads to the characterization of the ICA equivalence class of any directed graph 𝒢 as outlined in the following Lemma 17.

**Lemma 17 (Characterization of the ICA Equivalence Class)** *For any directed graph *𝒢* with *N* directed cycles *${𝒪}_{1},\ldots ,{𝒪}_{N}$ *(possibly joint with each other), all the directed graphs *${𝒢}^{\prime }$ *in its ICA equivalence class can be obtained by performing the following two steps:*

- *reversing* $N^{⋆}$ *of its disjoint cycles* ${\tilde{𝒪}}_{1},\ldots ,{\tilde{𝒪}}_{N^{⋆}}$, *where* ${\tilde{𝒪}}_{n}\cap {\tilde{𝒪}}_{n^{\prime }}=\emptyset$ *for* $1\leq n\neq n^{\prime }\leq N^{⋆},\left\{{\tilde{𝒪}}_{1},\ldots ,{\tilde{𝒪}}_{N^{⋆}}\right\}\subseteq \left\{{𝒪}_{1},\ldots ,{𝒪}_{N}\right\}$, *and* $1\leq N^{⋆}\leq N$;
- *changing the edge* $pa_{𝒢\setminus {\tilde{𝒪}}_{n^{⋆}}}\left(Y_{r_{k_{n^{⋆}}}}\right)\to Y_{r_{k_{n^{⋆}}}}$ *in* 𝒢 *to the edge* $pa_{𝒢\setminus {\tilde{𝒪}}_{n^{⋆}}}\left(Y_{r_{k_{n^{⋆}}}}\right)\to pa_{{\tilde{𝒪}}_{n^{⋆}}}\left(Y_{r_{k^{⋆}}}\right)$ *in* ${𝒢}^{\prime }$, *for* $k_{n^{⋆}}=1,\ldots ,K_{n^{⋆}}$, *where* ${\tilde{𝒪}}_{n^{⋆}}=\left\{Y_{r_{1}},\ldots ,Y_{r_{K_{n^{⋆}}}}\right\}$ *and* $n^{⋆}=1,\ldots ,N^{⋆}$.

*Additionally, applying steps (i) and (ii) to any disjoint cycles in any directed graph *𝒢 *will result in a directed graph *${𝒢}^{\prime }$ *that remains in the same ICA equivalence class as *𝒢.

For example, ${𝒢}_{2}^{+}$ in Figure 3 can be obtained by reversing ${𝒪}_{1}:Y_{1}\to Y_{2}\to Y_{3}\to Y_{1}$ in ${𝒢}_{1}^{+}$, and then changing the edges $Y_{4}\to Y_{3}$, $I_{Y_{1}}\to Y_{1}$, and $I_{Y_{2}}\to Y_{2}$ to $Y_{4}\to Y_{2}$, $I_{Y_{1}}\to Y_{3}$, and $I_{Y_{2}}\to Y_{1}$, respectively. Similarly, ${𝒢}_{3}^{+}$ in Figure 3 can be obtained by reversing ${𝒪}_{2}:Y_{1}\to Y_{4}\to Y_{3}\to Y_{1}$ in ${𝒢}_{1}^{+}$, and then changing the edges $Y_{2}\to Y_{3}$, $I_{Y_{1}}\to Y_{1}$, and $I_{Y_{4}}\to Y_{4}$ to $Y_{2}\to Y_{4}$, $I_{Y_{1}}\to Y_{3}$, and $I_{Y_{4}}\to Y_{1}$, respectively. Note that since ${𝒪}_{1}\cap {𝒪}_{2}=\left\{Y_{1},Y_{3}\right\}\neq \emptyset$ (i.e., ${𝒪}_{1}$ and ${𝒪}_{2}$ are joint with each other), there are no admissible row-permutations that can reverse both ${𝒪}_{1}$ and ${𝒪}_{2}$ in ${𝒢}_{1}^{+}$. Consequently, ${𝒢}_{2}^{+}$ and ${𝒢}_{3}^{+}$ are the only two other directed graphs in the ICA equivalence class of ${𝒢}_{1}^{+}$.

We now use the instrumental variable approach to achieve the unique identification of any directed graph 𝒢 with N directed cycles ${𝒪}_{1},\ldots ,{𝒪}_{N}$ (possibly joint with each other) in its ICA equivalence class. Suppose that for each directed cycle ${𝒪}_{n}$ in 𝒢, 1≤n≤N, there exists a variable $Y_{n}$ has its own instrumental variable $I_{Y_{n}}$. Let ${𝒢}^{+}$ denote the directed graph formed by incorporating these instrumental variables $ℐ=\left\{I_{Y_{1}},\ldots ,I_{Y_{N}}\right\}$ into 𝒢. By the definition of the instrumental variable (i.e., Definition 2), if ℐ introduces additional cycles in ${𝒢}^{+}$, then these cycles will *only* involve variables within ℐ and will remain disjoint from ${𝒪}_{1},\ldots ,{𝒪}_{N}$. By Lemma 17, the reversal of disjoint cycles is a necessary condition for two directed graphs to belong to the same ICA equivalence class. Therefore, there exists a one-to-many mapping from the ICA equivalence class of 𝒢 to the ICA equivalence class of ${𝒢}^{+}$ as they share common cycles. In particular, if ℐ introduces additional cycles, each graph in the ICA equivalence class of 𝒢 will correspond to multiple directed graphs in the ICA equivalence class of ${𝒢}^{+}$, and the latter only differ from each other in the part that *only* involves ℐ. On the other hand, the unique identification of 𝒢 is independent of the part that *only* involves ℐ (see details in Appendix A.1). Therefore, the unique identification of 𝒢 can be derived from the unique identification of ${𝒢}^{+}$.

Consider any directed cyclic graph ${\left({𝒢}^{+}\right)}^{\prime }$ in the ICA equivalence class of ${𝒢}^{+}$ obtained by performing the two steps described in Lemma 17. Note that by the definition of instrumental variable (i.e., Definition 2), $I_{Y_{n^{⋆}}}\in {𝒢}^{+}\setminus {\tilde{𝒪}}_{n^{⋆}}$ is a special case of ${\text{pa}}_{{𝒢}^{+}\setminus {\tilde{𝒪}}_{n^{⋆}}}\left(Y_{n^{⋆}}\right)$, where $Y_{n^{⋆}}\in {\tilde{𝒪}}_{n^{⋆}}$ is the only child of $I_{Y_{n^{⋆}}}$ in 𝒢, for $n^{⋆}=1,\ldots ,N^{⋆}$. According to Lemma 17(ii), the edge $I_{Y_{n^{⋆}}}\to Y_{n^{⋆}}$ in ${𝒢}^{+}$ will be changed to $I_{Y_{n^{⋆}}}\to {\text{pa}}_{{\tilde{𝒪}}_{n^{⋆}}}\left(Y_{n^{⋆}}\right)$ in ${\left({𝒢}^{+}\right)}^{\prime }$. In other words, the only child of $I_{Y_{n^{⋆}}}$ will be different in 𝒢 and ${𝒢}^{\prime }$ (i.e., $Y_{n^{⋆}}\neq {\text{pa}}_{{\tilde{𝒪}}_{n^{⋆}}}\left(Y_{n^{⋆}}\right)$ due to no self-loops), and thus $I_{Y_{n^{⋆}}}$ will not be an instrumental variable for $Y_{n^{⋆}}$ in ${\left({𝒢}^{+}\right)}^{\prime }$. Therefore, we can identify ${𝒢}^{+}$ from ${\left({𝒢}^{+}\right)}^{\prime }$ by utilizing a collection of instrumental variables $I_{Y_{n^{⋆}}}$ for $Y_{n^{⋆}}\in {\tilde{𝒪}}_{n^{⋆}}$, where $n^{⋆}=1,\ldots ,N^{⋆}$. We finish the proof of Theorem 9 by noting that the above argument can be applied to any ${\left({𝒢}^{+}\right)}^{\prime }$ within the ICA equivalence class of ${𝒢}^{+}$.

In summary, in the proof of Theorem 9, we first establish an equivalent form of applying an admissible irreducible row-permutation to an unmixing matrix and conducting two operations on the directed graph associated with the unmixing matrix in Lemma 14, then extend this result from admissible irreducible row-permutations to admissible row-permutations by illustrating their connection in Proposition 16. This leads to the characterization of the ICA equivalence class, as outlined in Lemma 17. We then establish a one-to-many mapping from the ICA equivalence class of any directed cyclic graph 𝒢 to the ICA equivalence class of ${𝒢}^{+}$, which is the augmented graph obtained by incorporating all instrumental variables into 𝒢. The proof is concluded by demonstrating the unique identification of 𝒢 based on the unique identification of ${𝒢}^{+}$, utilizing a collection of instrumental variables.

Lastly, in the following corollary of Theorem 9, we prove the unique causal identifiability of the proposed model (1).

**Corollary 18** *Let* ${𝒢}_{j}=\left({𝒱}_{j},{ℰ}_{j}\right)$* denote the directed cyclic graph such that *${𝒱}_{j}=\left\{Y_{j1},\ldots ,Y_{jQ}\right\}$, *and *$\left(Y_{jq}\leftarrow Y_{jp}\right)\in {ℰ}_{j}$* if and only if* $\beta_{0qp}\neq 0,1\leq p,q\leq Q$. *Suppose that Assumptions 1–2 hold, then the causal structure of the proposed model *(1)* is uniquely identifiable if for any cycle *${𝒪}_{j}$ *in *${𝒢}_{j}$,* there exists a variable *$Y_{jq}\in {𝒪}_{j}$* has its own instrumental variable *$I_{Y_{jq}}$.

There are two possible sources of the instrumental variable $I_{Y_{jq}}$ for the longitudinal health outcome $Y_{jq}$ at each time step j in the proposed model (1). The first source is the covariate $X_{j-\ell ,s},\ell \geq 0$, e.g., $I_{Y_{j1}}=X_{j-1,1}$ and $I_{Y_{j4}}=X_{j2}$ as shown in Figure 1. Note that $X_{j1}$ can not be used as an instrumental variable for $Y_{j1}$ since it has another child $Y_{j3}$ in ${𝒢}_{j}$. In practice, suitable covariates that satisfy the condition of instrumental variables may or may not exist. Therefore, for models that only consider instantaneous causal relationships (e.g., the SCM (4)), verifying unique causal identifiability becomes challenging when such covariates do not exist. Fortunately, we can find instrumental variables in the proposed model (1) by taking advantage of the longitudinal data. Specifically, the second source is the previous measurement of the longitudinal health outcome $Y_{j-\ell ,q},\ell >0$, e.g., $I_{Y_{j3}}=Y_{j-1,3}$ and $I_{Y_{j4}}=Y_{j-2,4}$ as shown in Figure 1. This is because in longitudinal data, the causal influence of a variable $Y_{jq}$ from its previous measurement $Y_{j-\ell ,q},\ell >0$, typically exhibits a slower rate of decay compared to the causal effects originating from previous measurements of other variables $Y_{j-\ell ,p},\ell >0,p\neq q$. As a result, it is highly plausible that an instrumental variable can be identified at some time lag ℓ>0 for each variable, i.e., $I_{Y_{jq}}=Y_{j-\ell ,q}$.

## 4. Bayesian Structural Learning

We have shown that the proposed model is identifiable from observational data. Therefore, structural learning can be carried out with any appropriate estimation procedure. In this work, we adopt a Bayesian approach due to two reasons. First, it yields a computationally efficient inference procedure through posterior computation with uncertainty quantification. Second, it provides a flexible framework to incorporate prior knowledge and imposes sparsity through prior distributions for better interpretability of the inferred causal graph.

Specifically, we assign spike-and-slab priors (Ishwaran and Rao, 2005) on both $\beta_{\ell qp}$ and $\alpha_{\ell qs}$ in Equation (1) to select a sparse set of the causes. We describe the prior for $\beta_{\ell qp}$, and the prior for $\alpha_{\ell qs}$ is analogously defined. We assume that $\beta_{\ell qp}\sim 𝒩\left(0,\gamma_{\ell qp}\nu_{\ell qp}\right)$ with $\nu_{\ell qp}\sim \text{Inverse-Gamma}\left(a_{\nu },b_{\nu }\right)$ and $\gamma_{\ell qp}\sim \rho \delta_{1}\left(\gamma_{\ell qp}\right)+(1-\rho )\delta_{\nu_{0}}\left(\gamma_{\ell qp}\right)$, where $\delta_{x}(\cdot )$ denotes the Dirac measure at x. The hyper-parameter $\nu_{0}$ is a very small, pre-specified value. In particular, if $\gamma_{\ell qp}=1$ (slab), $\beta_{\ell qp}$ is non-zero, which suggests that there exists a causal effect of $Y_{j-\ell ,p}$ on $Y_{jq}$; if $\gamma_{\ell qp}=\nu_{0}$ (spike), $\beta_{\ell qp}$ is almost negligible and can be safely treated as zero (i.e., $\beta_{\ell qp}\approx 0$), which in turn implies that there is no significant causal effect of $Y_{j-\ell ,p}$ on $Y_{jq}$. We assume $\rho \sim \text{Beta}\left(a_{\rho },b_{\rho }\right)$ following the idea of Scott and Berger (2010).

Furthermore, we assume that the non-Gaussian error $e_{jq}$ follows a Laplace distribution (Choi and Hobert, 2013), i.e., $e_{jq}\sim \text{Laplace}\left(0,2\sigma_{q}\right)$, which can be viewed as a continuous scale mixture of normal distributions. Specifically, let $e_{jq}$ and $\tau_{jq}$ be pairs of independent random variables such that $e_{jq}|\tau_{jq}\sim 𝒩\left(0,\sigma_{q}^{2}/\tau_{jq}\right)$, and $\tau_{jq}\sim \text{Inverse-Gamma}(1,1/8)$, then marginally $e_{jq}\sim \text{Laplace}\left(0,2\sigma_{q}\right)$. In addition, we assign $\mu_{q}\sim 𝒩\left(0,\sigma_{\mu }^{2}\right)$ and $\sigma_{q}^{2}\sim \text{Inverse-Gamma}\left(a_{\sigma },b_{\sigma }\right)$, which result in closed-form full conditionals for ease of posterior computation. We carry out posterior inference using a standard Markov chain Monte Carlo (MCMC) algorithm, the details of which are included in Appendix B.

Lastly, by Corollary 18, the unique identifiability of the proposed model (1) relies on the presence of a sufficient set of instrumental variables. Therefore, a post-hoc validation step becomes essential to ensure the learned causal graph 𝒢 achieves unique identifiability. When such a sufficient set cannot be identified, the output of Bayesian structural learning consists of all directed graphs in the ICA equivalence class of 𝒢 associated with stable SCMs (since we impose the stability condition on the instantaneous causal effects $B_{0}$ in the MCMC algorithm, as described in Step 1.1 in Appendix B). In contrast, when a sufficient set of instrumental variables is available, the unique causal identifiability of 𝒢 within its ICA equivalence class is guaranteed.

## 5. Simulation Study

In this section, we conducted a series of simulation studies to empirically verify our causal identification theory established in Section 3, and evaluate the performance of the Bayesian structural learning algorithm proposed in Section 4.

### 5.1 Simulation Scenario I

**Figure 4:**
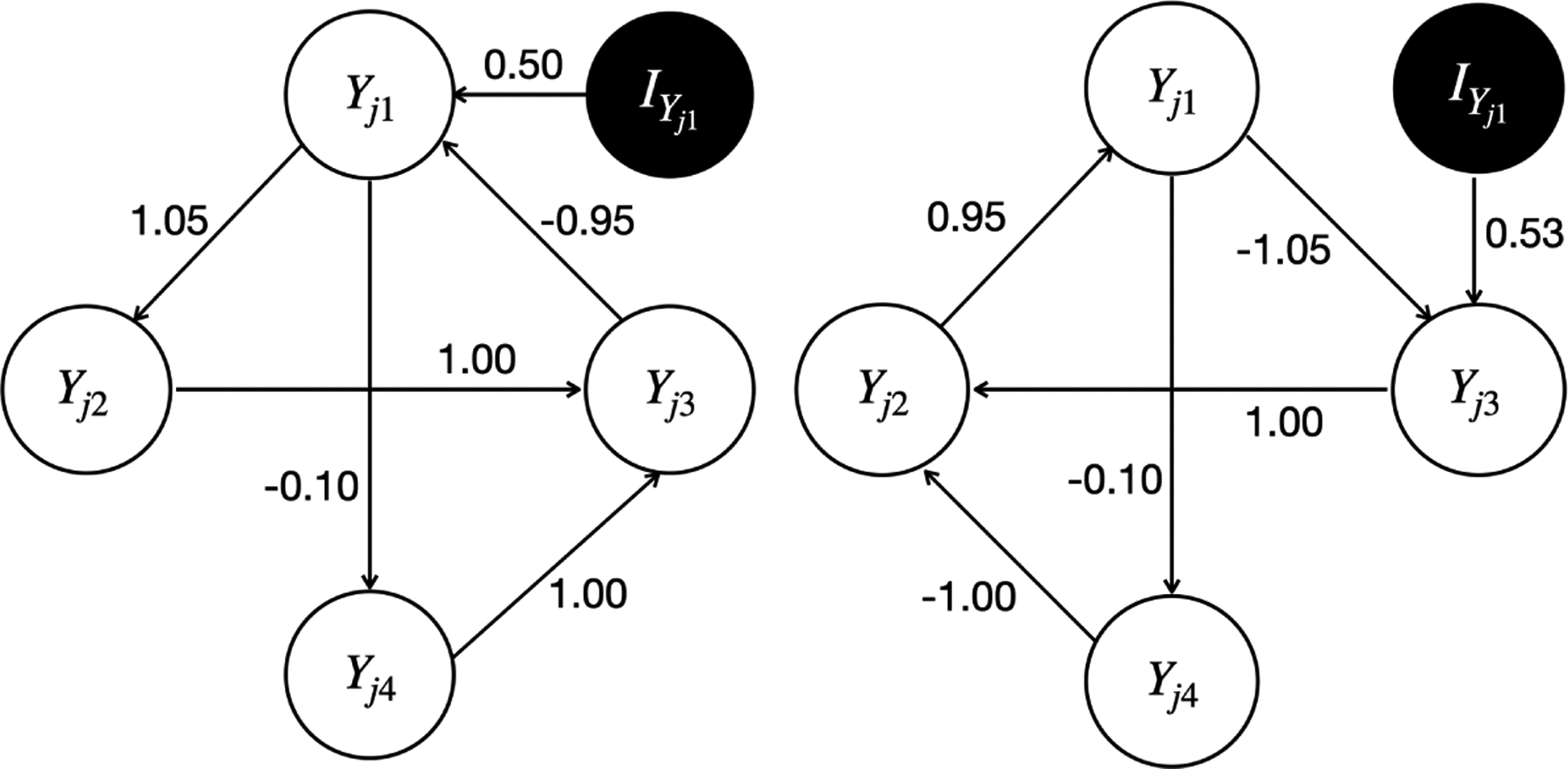
The simulated true causal graph 𝒢 (left) and the only graph ${𝒢}^{\prime }$ (right) associated with a stable SCM in its ICA equivalence class in simulation scenario I. The instrumental variable $I_{Y_{j1}}$ for $Y_{j1}$ is highlighted by the black circle.

This scenario was designed to empirically verify our causal identification theory. The simulated true causal graph was set to be 𝒢 in the left panel of Figure 4 with one instrumental variable $I_{Y_{j1}}$ for $Y_{j1}$. The right panel of Figure 4 plots the only graph ${𝒢}^{\prime }$ in the ICA equivalence class of 𝒢 for which the corresponding SCM is stable. We considered two types of instrumental variables: $I_{Y_{j1}}=X_{j1}\sim N(0,1)$, and $I_{Y_{j1}}=Y_{j-1,1}$. We set the simulated true values for the intercepts to be $\mu_{q}=0$, and the variances of the Laplace errors to be 1, yielding $\sigma_{q}^{2}=1/8$, for q=1,…,Q, where Q=4. Assume that there were 200, 500, and 1, 000 individuals, each of which had $J_{i}=5$ longitudinal observations, yielding a total sample size of 1, 000, 2, 500, and 5, 000. Then we generated the simulated true $Y_{ijq}$ using the following data-generating process,

(5) $$\left\{\begin{array}{l} Y_{ij1}=\mu_{1}-0.95\times Y_{ij3}+0.5\times I_{Y_{ij1}}+e_{ij1},\\ Y_{ij2}=\mu_{2}+1.05\times Y_{ij1}+e_{ij2},\\ Y_{ij3}=\mu_{3}+1\times Y_{ij2}+1\times Y_{ij4}+e_{ij3},\\ Y_{ij4}=\mu_{4}-0.1\times Y_{ij1}+e_{ij4}. \end{array}\right.$$

The data-generating process in this study is specific to scenarios where certain conditions are met, such as the existence of multiple directed graphs with joint cycles within the same ICA equivalence class, each corresponding to a stable SCM. We believe that this focus is necessary to fully demonstrate the strengths of the proposed model.

We applied the proposed Bayesian structural learning algorithm to the simulated datasets with the following hyper-parameter values: $\nu_{0}=2.5e-4$, $a_{\nu }=5$, $b_{\nu }=50$, $a_{\rho }=b_{\rho }=0.5$, $\sigma_{\mu }^{2}=100$, and $a_{\sigma }=b_{\sigma }=1$. For each configuration of the simulated dataset, we repeated the experiment with 100 replications. We ran 5,000 MCMC iterations with an initial burnin of 2,500 iterations and a thinning factor of 5 for each analysis. For the number of time lags in the proposed model (1), we fixed $L_{y}=L_{x}=0$ when $I_{Y_{j1}}=X_{j1}$, and set $L_{y}=1$ and $L_{x}=0$ when $I_{Y_{j1}}=Y_{j-1,1}$. To determine whether the estimated coefficients are zeros or non-zeros, we used the median probability model criteria (Barbieri and Berger, 2004). If $P\left(\gamma_{\ell qp}=1\right)>0.5$ calculated from the post-burn-in MCMC samples, we included an edge $Y_{j-\ell ,p}\to Y_{jq}$. The same rule was used for determining the existence of $X_{j-\ell ,s}\to Y_{jq}$.

For comparison, we considered three alternative methods. The first method is LiNG-D (Lacerda et al., 2008), which is an ICA-based causal discovery approach for cross-sectional data. While LiNG-D accounts for directed cycles, it does not incorporate covariates into its analysis. The other two methods are state-of-the-art time-series causal discovery techniques called VAR-LiNGAM (Hyvärinen et al., 2010) and PCMCI^+^ (Runge, 2020). VAR-LiNGAM is a two-step method that first estimates time-lagged causalities using VAR (Swanson and Granger, 1997) and then estimates instantaneous causalities by applying LiNGAM algorithm (Shimizu et al., 2006) on the residuals of the first step. PCMCI^+^ is a constraint-based approach that exploits conditional independencies to first build a skeleton of the causal graph, and then orient the skeleton according to a set of rules that define constraints on admissible orientations. Although VAR-LiNGAM and PCMCI^+^ are capable of detecting both time-lagged and instantaneous causal relationships, they do not handle directed cycles. VAR-LiNGAM also does not consider covariates.

We implemented LiNG-D, VAR-LiNGAM, and PCMCI^+^ using python packages py-tetrad, causal-learn, and tigramite, respectively. We set the hyper-parameters threshold_b = 0.1 and threshold_w = 0.1 as their default values for LiNG-D, and set the maximal time lag to lags = 2 for VAR-LiNGAM. For PCMCI^+^, we used RobustParCorr to test the conditional independence for all continuous data, and RegressionCI for mixed data. In addition, we used the J-PCMCI^+^ algorithm (Günther et al., 2023) to accommodate covariates, set the maximal time lag to $\tau_{\text{max}}=2$, and determined the significance level $pc_{\alpha }$ to be the optimal one in the set {0.01, 0.025, 0.05}. Since all the alternative methods do not account for individual-level information (carried by index i in the proposed model), we adopted the following data-generating process for fair comparison. For each simulated dataset used for the proposed model, we excluded its individual pattern by generating a corresponding dataset with the same sample size of $\sum_{i}J_{i}$ for each variable $Y_{jq},j=1,\ldots ,\sum_{i}J_{i},q=1,\ldots ,Q$.

**Figure 5:**
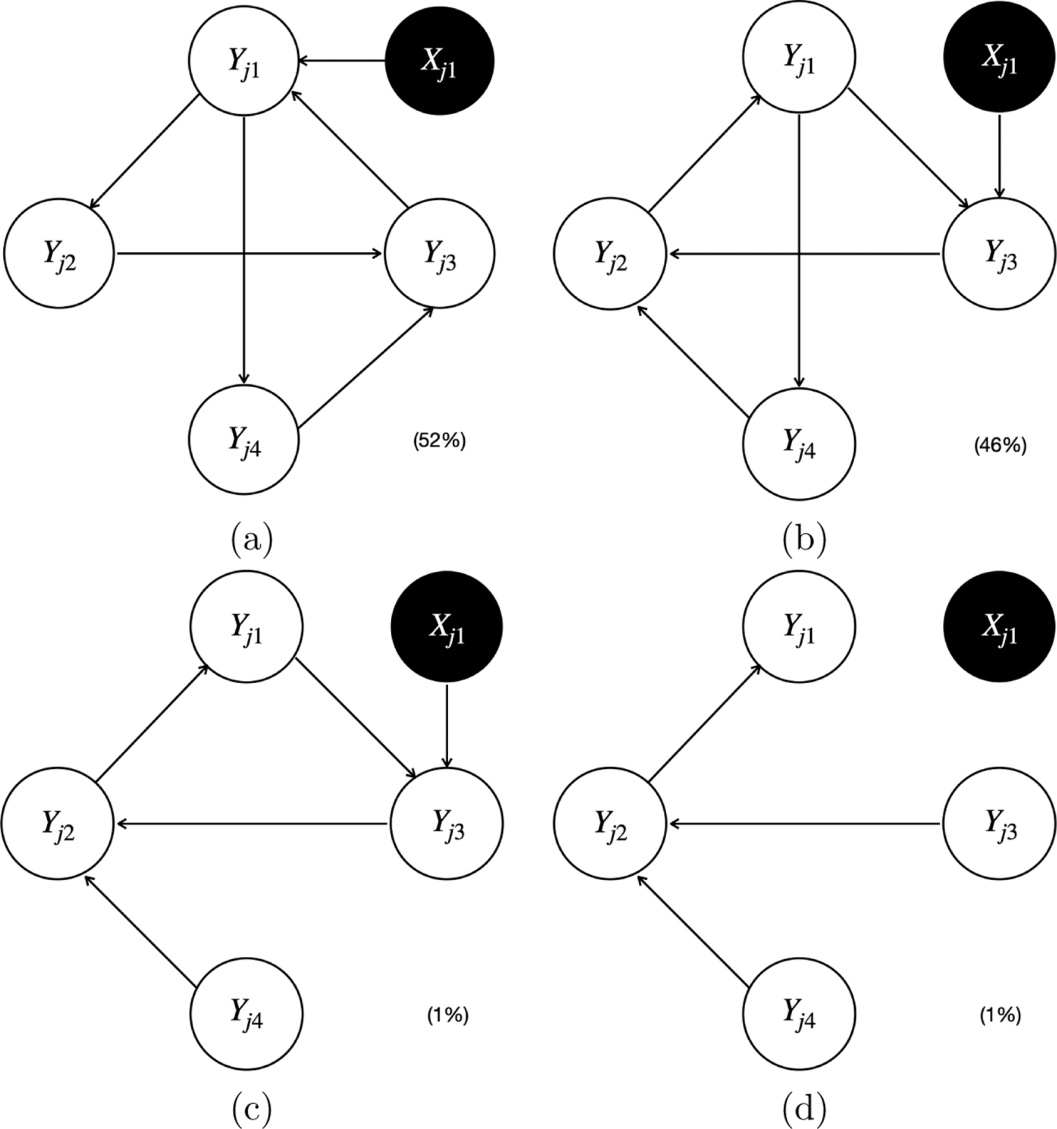
All four individual causal graphs identified by the proposed model in simulation scenario I with a sample size of 5,000, using $X_{j1}$ as the instrumental variable $I_{Y_{j1}}$ (highlighted by the black circles). The percentage within the parenthesis indicates the relative frequency of detecting the corresponding causal graph across 100 replications.

**Figure 6:**
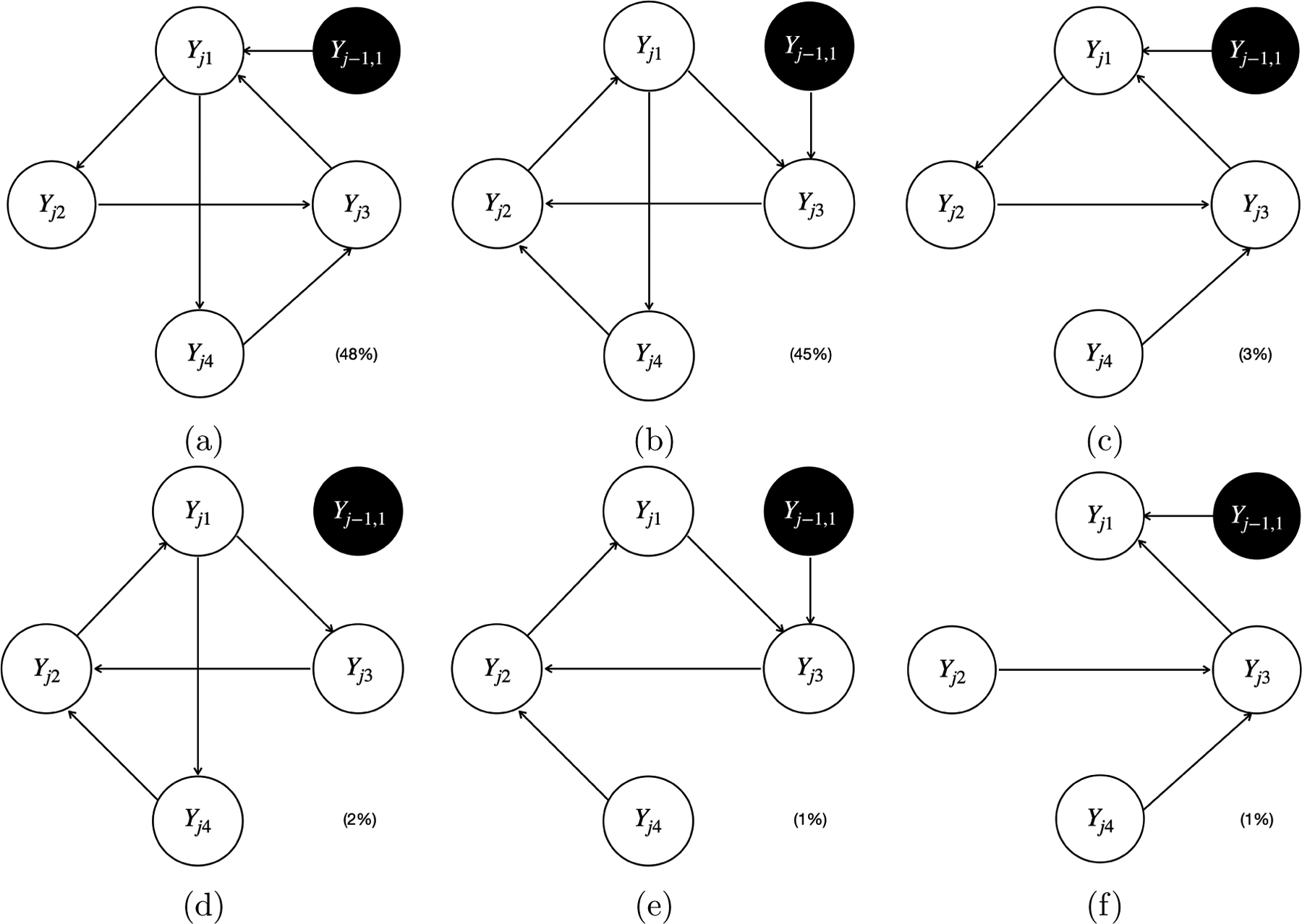
All six individual causal graphs identified by the proposed model in simulation scenario I with a sample size of 5,000, using $Y_{j-1,1}$ as the instrumental variable $I_{Y_{j1}}$ (highlighted by the black circles). The percentage within the parenthesis indicates the relative frequency of detecting the corresponding causal graph across 100 replications.

We now present the simulation results for scenario I. Figure 5 and Figure 6 plot all the individual causal graphs identified by the proposed model when $X_{j1}$ and $Y_{j-1,1}$ were used as the instrumental variable $I_{Y_{j1}}$ (highlighted by the black circles) respectively, with a sample size of 5,000. The percentage within the parenthesis indicates the relative frequency of detecting the corresponding causal graph across 100 replications.

The estimated causal graphs under the proposed model have two distinct modes ${\overset{ˆ}{𝒢}}_{\text{Proposed}}^{\left(1\right)}$ (i.e., Figure 5(a) and Figure 6(a)) and ${\hat{𝒢}}_{\text{Proposed}}^{\left(2\right)}$ (i.e., Figure 5(b) and Figure 6(b)), where the first one corresponds to the simulated true graph 𝒢, and the second one corresponds to the only graph ${𝒢}^{\prime }$ associated with a stable SCM in the ICA equivalence class of 𝒢. Note that Figure 5(c)&(d) are slight variations of Figure 5(b), Figure 6(c)&(f) are slight variations of Figure 6(a), and Figure 6(d)&(e) are slight variations of Figure 6(b). These slight variations in certain missing edges (e.g., $Y_{j1}\to Y_{j4}$ in Figure 5(c) compared to Figure 5(b)) are possibly due to finite sample size and MCMC iterations. The existence of two distinct modes ${\hat{𝒢}}_{\text{Proposed}}^{\left(1\right)}$ and ${\hat{𝒢}}_{\text{Proposed}}^{\left(2\right)}$ indicates the observation equivalence between 𝒢 and ${𝒢}^{\prime }$. Therefore, it is impossible to distinguish 𝒢 from ${𝒢}^{\prime }$ without the help of instrumental variables. If we can not identify a sufficient set of instrumental variables, the output of our proposed Bayesian structural learning algorithm will consist of the ICA equivalence class $\left\{𝒢,{𝒢}^{\prime }\right\}$. However, by Corollary 18, the proposed model is uniquely identifiable, since there exists an instrumental variable $I_{Y_{j1}}$ for $Y_{j1}\in {𝒪}_{j1}\cap {𝒪}_{j2}$, where ${𝒪}_{j1}:Y_{j1}\to Y_{j2}\to Y_{j3}\to Y_{j1}$ and ${𝒪}_{j2}:Y_{j1}\to Y_{j4}\to Y_{j3}\to Y_{j1}$. Specifically, we can uniquely identify 𝒢 within its ICA equivalence class $\left\{𝒢,{𝒢}^{\prime }\right\}$ by noting that the child of $I_{Y_{j1}}$ is $Y_{j1}$ in 𝒢, whereas in ${𝒢}^{\prime }$, it is $Y_{j3}$. Importantly, this unique identification holds for either $I_{Y_{j1}}=X_{j1}$ or $I_{Y_{j1}}=Y_{j-1,1}$, which empirically verified our causal identification theory.

**Figure 7:**
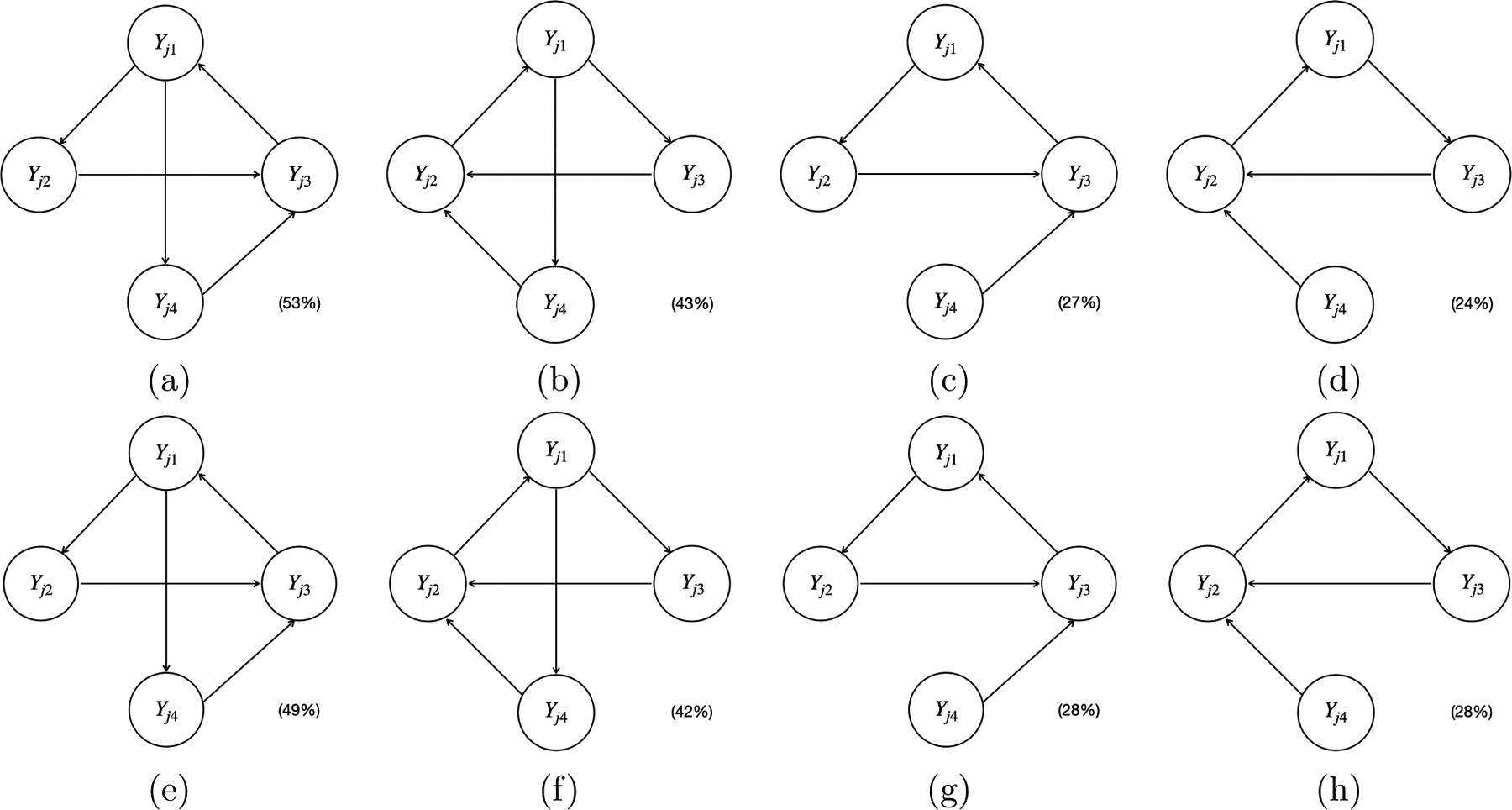
The estimated causal graphs under LiNG-D with a sample size of 5,000, using $X_{j1}$ (Panel(a)-(d)) and $Y_{j-1,1}$ (Panel(e)-(h)) as the instrumental variable $I_{Y_{j1}}$ (highlighted by the black circles) in simulation scenario I. The percentage within the parenthesis indicates the relative frequency of detecting the corresponding causal graph across 100 replications.

**Figure 8:**
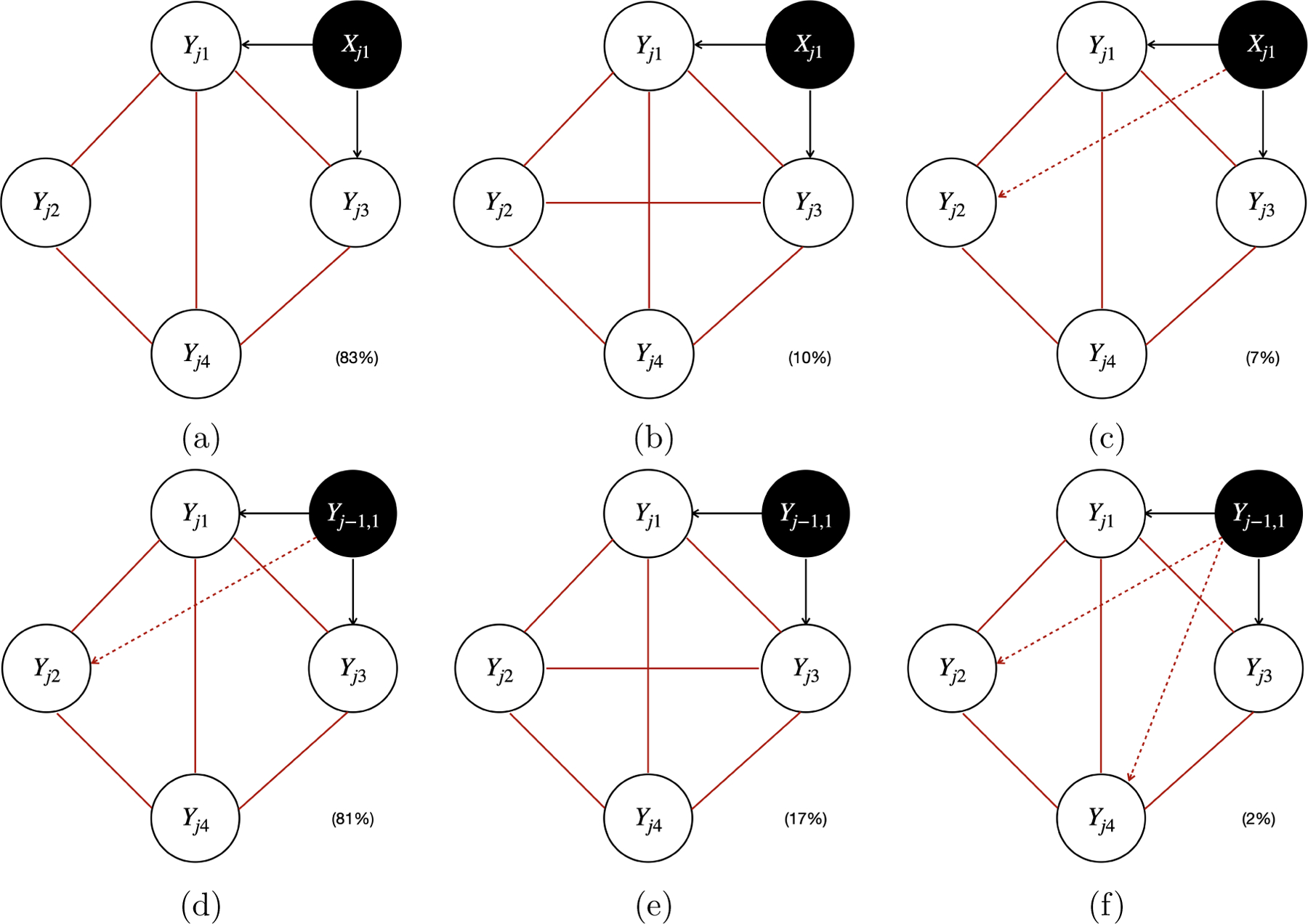
The estimated causal graphs under PCMCI^+^ with a sample size of 5,000, using $X_{j1}$ (Panel(a)-(c)) and $Y_{j-1,1}$ (Panel(d)-(f)) as the instrumental variable $I_{Y_{j1}}$ (highlighted by the black circles) in scenario I. The solid red lines and dashed red arrows indicate the unoriented and spurious causal links, respectively. The percentage within the parenthesis indicates the relative frequency of detecting the corresponding graph across 100 replications.

**Figure 9:**
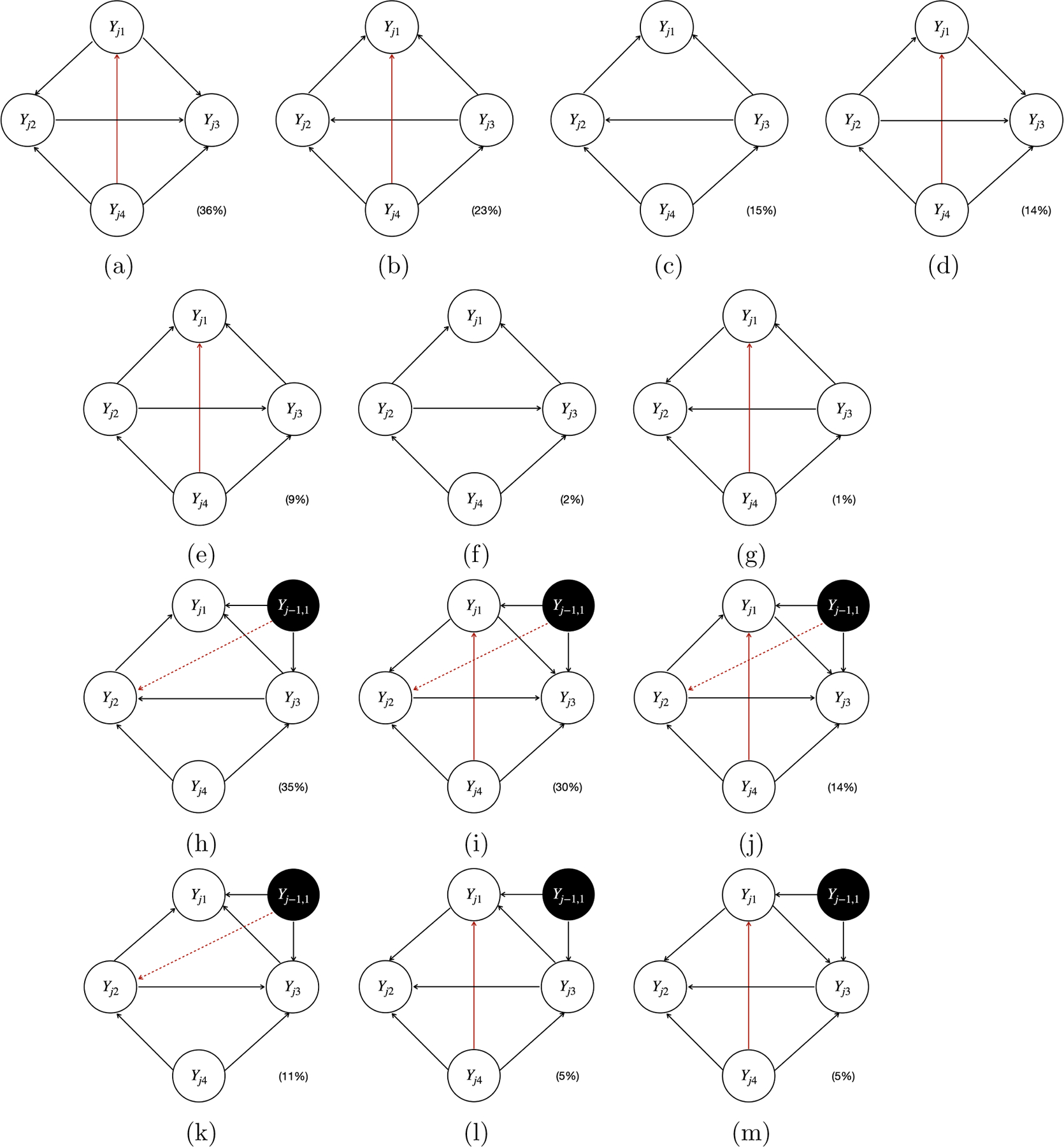
The estimated causal graphs under VAR-LiNGAM with a sample size of 5,000, using $X_{j1}$ (Panel(a)-(g)) and $Y_{j-1,1}$ (Panel(h)-(m)) as the instrumental variable $I_{Y_{j1}}$ (highlighted by the black circles) in scenario I. The solid and dashed red arrows indicate the reversed and spurious causal links, respectively. The percentage within the parenthesis indicates the relative frequency of detecting the corresponding graph across 100 replications.

Furthermore, Figure 7, Figure 8, and Figure 9 plot the estimated causal graphs under the alternative methods (i.e., LiNG-D, PCMCI^+^, and VAR-LiNGAM) when $X_{j1}$ and $Y_{j-1,1}$ were used as the instrumental variable $I_{Y_{j1}}$ (highlighted by the black circles) with a sample size of 5,000. The solid red lines indicate the unoriented causal links. The solid and dashed red arrows indicate reversed and spurious causal links, respectively.

Depending on the specific estimation procedures, the equivalence classes of the estimated causal graphs reported by different methods displayed distinct patterns. Specifically, the proposed model, PCMCI^+^, and VAR-LiNGAM output a single causal graph for each experiment, leading to the sum of the percentages across all graphs being equal to 100% (e.g., 52%+46%+1%+1%=100% for the proposed model shown in Figure 5, 83%+10%+7%=100% for PCMCI^+^ shown in Figure 8(a)–(c), and 36%+23%+15%+14%+9%+2%+1%=100% for VAR-LiNGAM shown in Figure 9(a)–(g)). LiNG-D output either a single causal graph or two equivalent causal graphs for each experiment, resulting in a sum of percentages across both modes exceeding 100% (e.g., 53%+43%+27%+24%>100% shown in Figure 7(a)–(d)).

LiNG-D identified two distinct modes of the causal graphs ${\overset{ˆ}{𝒢}}_{\text{LiNG-D}}^{\left(1\right)}$ (i.e., Figure 7(a)&(e)) and ${\hat{𝒢}}_{\text{LiNG-D}}^{\left(2\right)}$ (i.e., Figure 7(b)&(f)) among 100 replications. Note that Figure 7(c), (d), (g), and (h) are slight variations of Figure 7(a), (b), (e), and (f), respectively, all of which missed the edge $Y_{j1}\to Y_{j4}$, possibly due to finite sample size. Despite recovering the ICA equivalence class $\left\{𝒢,{𝒢}^{\prime }\right\}$, LiNG-D was unable to differentiate between 𝒢 and ${𝒢}^{\prime }$. This is because LiNG-D does not exploit covariates or time-lagged variables as instrumental variables for unique causal identification as in the proposed model. In contrast, both VAR-LiNGAM and PCMCI^+^ did not identify the ICA equivalence class $\left\{𝒢,{𝒢}^{\prime }\right\}$. Specifically, due to relying on the assumption of acyclicity, VAR-LiNGAM identified a Markov equivalence class comprising acyclic graphs that resemble the ICA equivalence class of cyclic graphs. For example, in order to prevent the formation of cycles, VAR-LiNGAM reversed the edge from $Y_{j1}\to Y_{j4}$ to $Y_{j1}\leftarrow Y_{j4}$. PCMCI^+^ detected all the edges appearing in both 𝒢 and ${𝒢}^{\prime }$. Since PCMCI^+^ relies on the assumption of acyclicity, all the edges among $Y_{j}$’s remained unoriented. When PCMCI^+^ detected an unoriented causal relationship between $Y_{jq}$ and $Y_{jp}$, two possible scenarios arose: (i) the estimated graphs under PCMCI^+^ with either $Y_{jq}\leftarrow Y_{jp}$ or $Y_{jq}\to Y_{jp}$ were observationally equivalent; (ii) conflicting orientations $Y_{jq}\leftarrow Y_{jp}$ and $Y_{jq}\to Y_{jp}$ were suggested by different orientation rules, possibly due to finite sample size or violations of the assumption of acyclicity. VAR-LiNGAM and PCMCI^+^ also detected a spurious causal link between $Y_{j-1,1}$ and $Y_{j2}$ that does not appear in either 𝒢 or ${𝒢}^{\prime }$.

**Figure S2:**
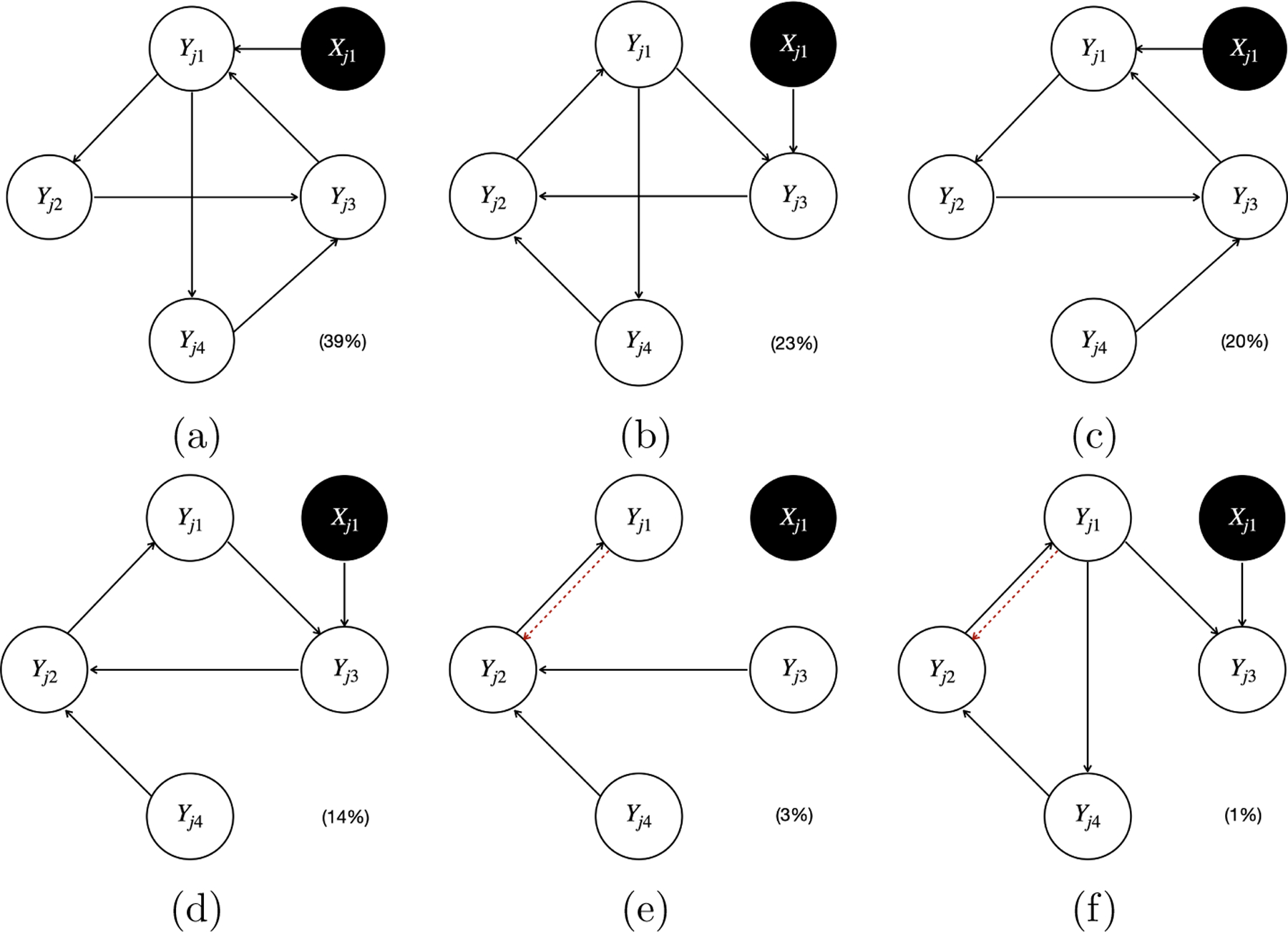
The estimated causal graphs under the proposed model with a sample size of 2,500, using $X_{j1}$ as the instrumental variable $I_{Y_{j1}}$ in simulation scenario I.

**Figure S3:**
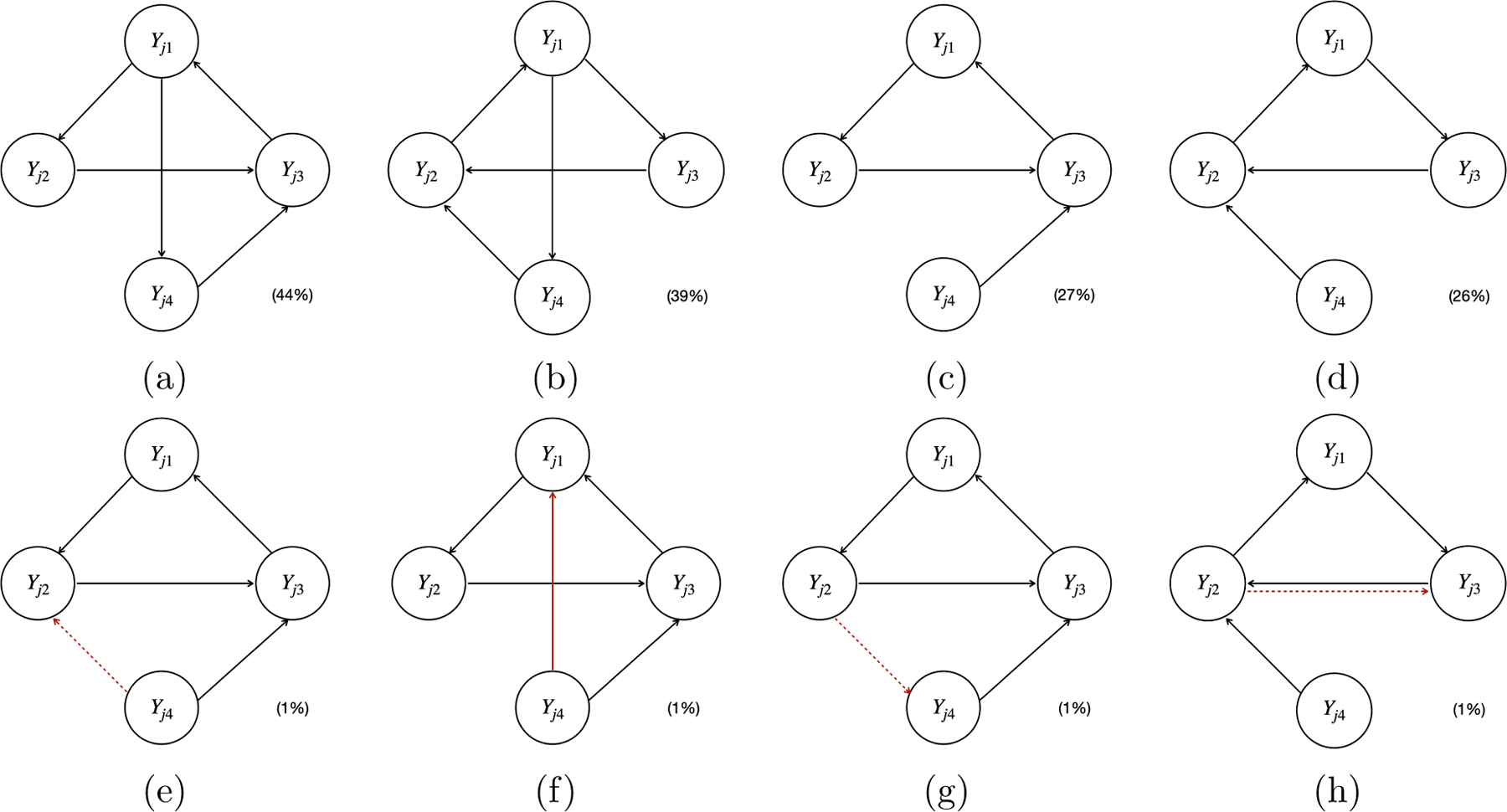
The estimated causal graphs under LiNG-D with a sample size of 2,500, using $X_{j1}$ as the instrumental variable $I_{Y_{j1}}$ in simulation scenario I.

**Figure S4:**
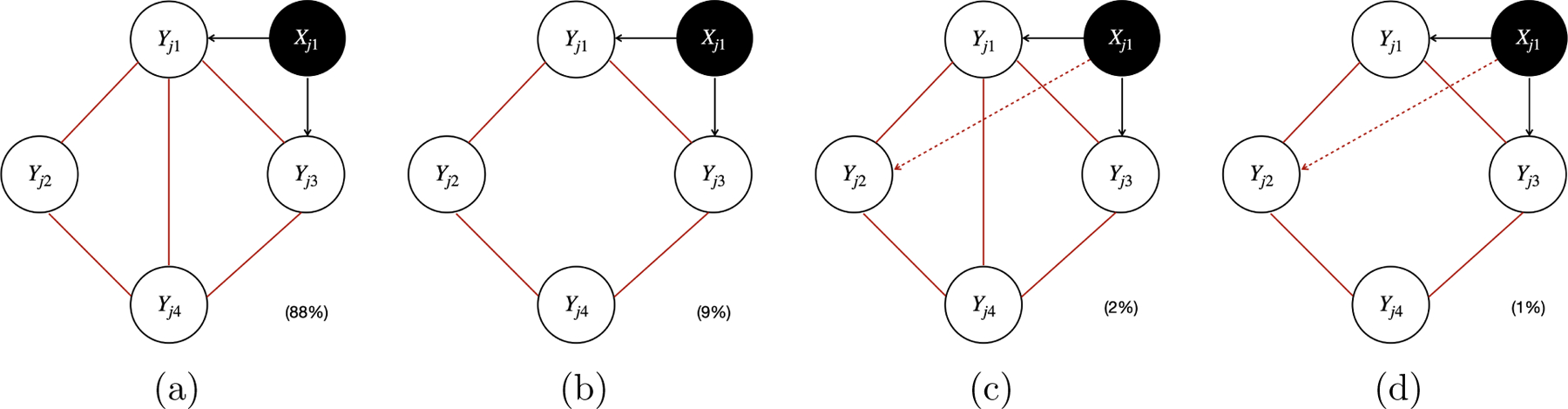
The estimated causal graphs under PCMCI^+^ with a sample size of 2,500, using $X_{j1}$ as the instrumental variable $I_{Y_{j1}}$ in simulation scenario I.

**Figure S5:**
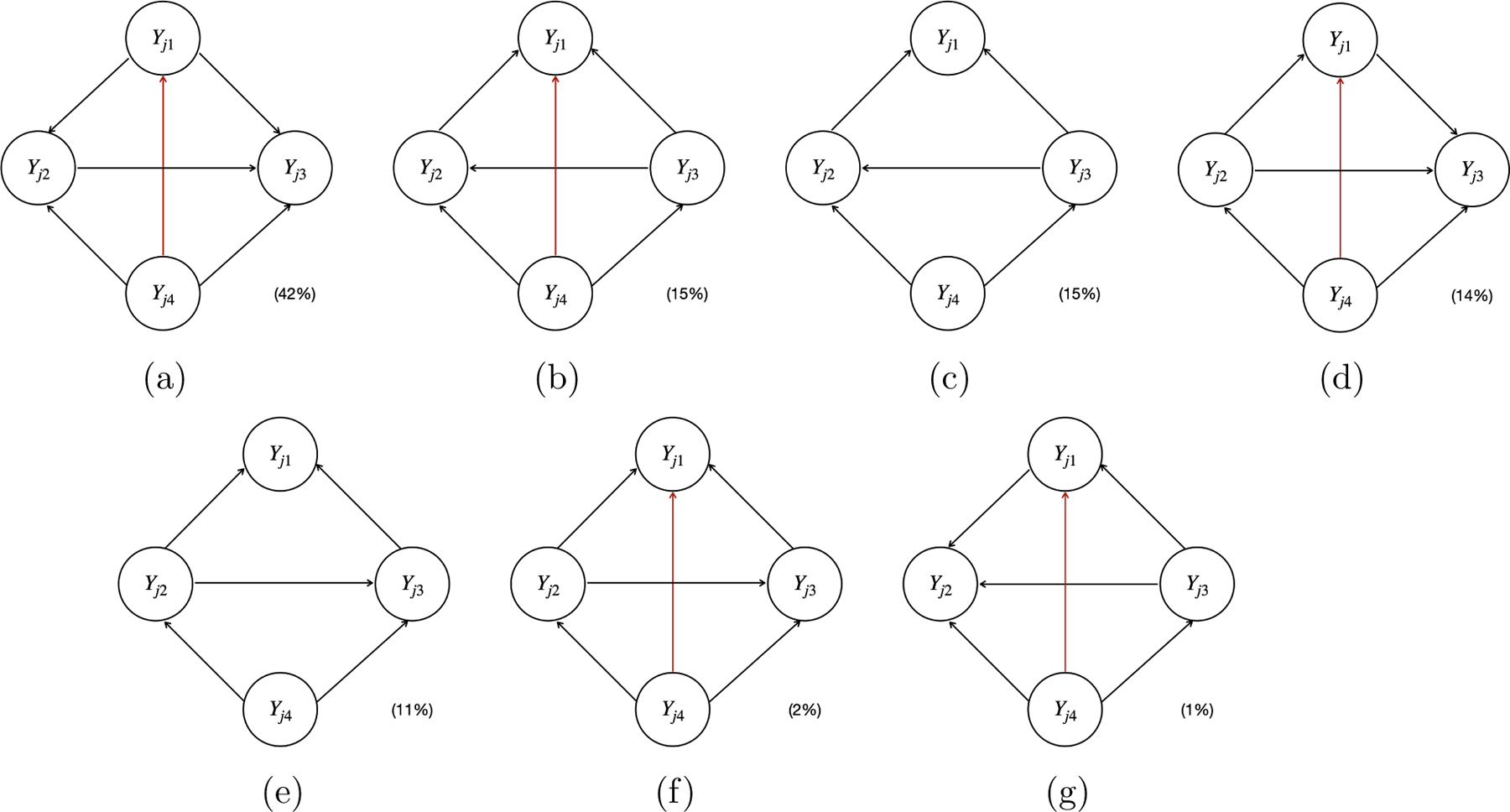
The estimated causal graphs under VAR-LiNGAM with a sample size of 2,500, using $X_{j1}$ as the instrumental variable $I_{Y_{j1}}$ in simulation scenario I.

**Figure S6:**
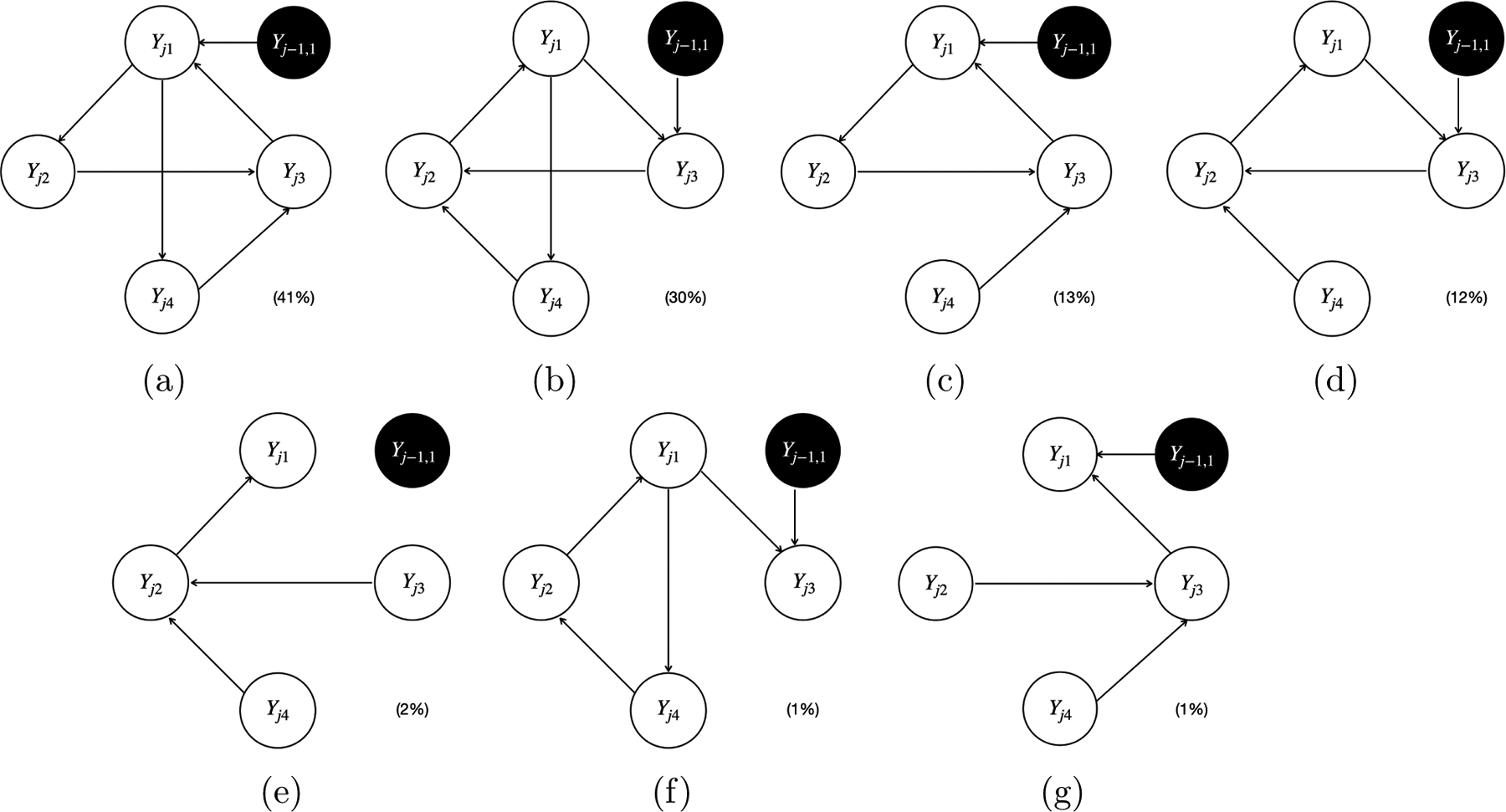
The estimated causal graphs under the proposed model with a sample size of 2,500, using $Y_{j-1,1}$ as the instrumental variable $I_{Y_{j1}}$ in simulation scenario I.

**Figure S7:**
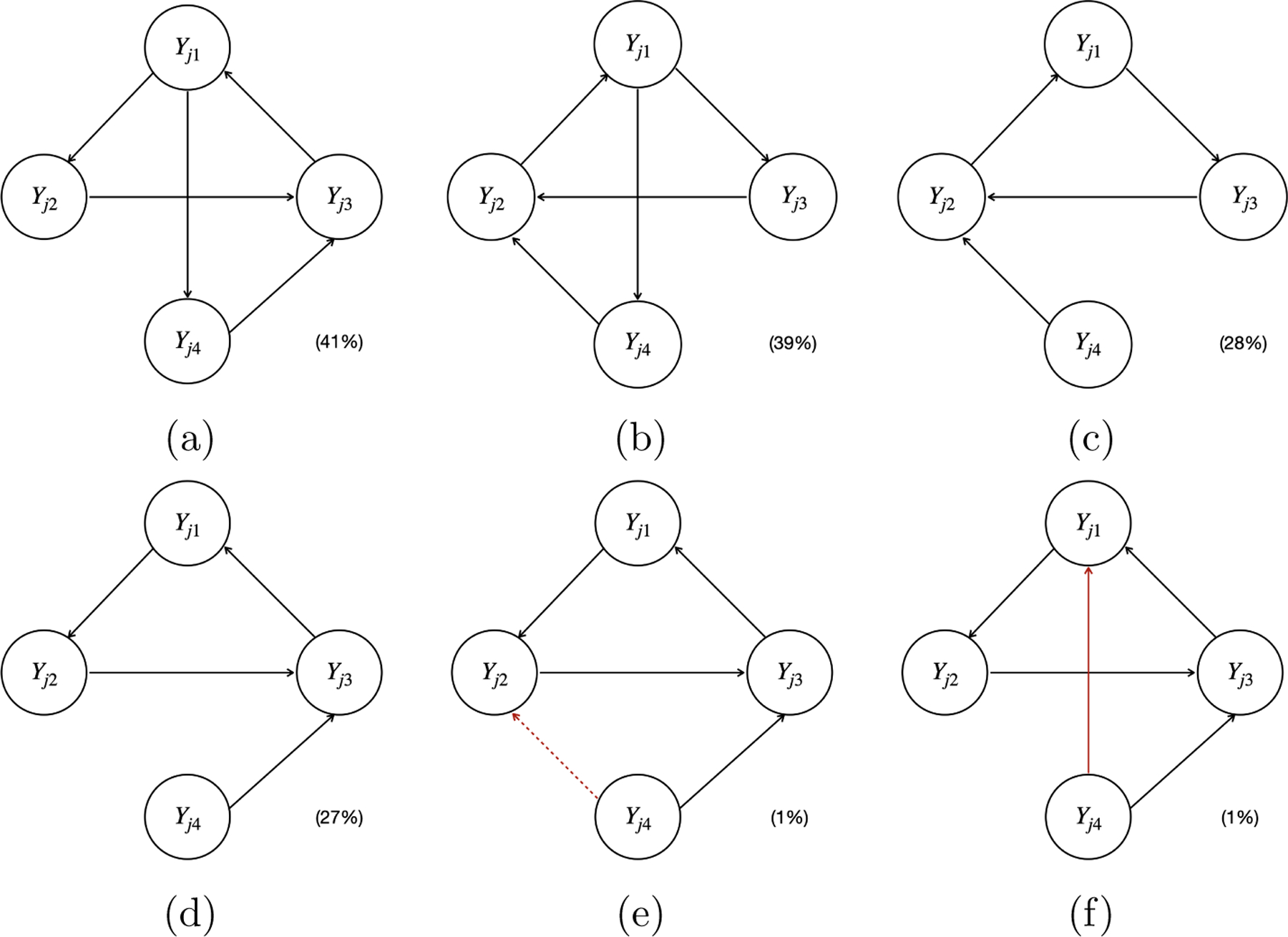
The estimated causal graphs under LiNG-D with a sample size of 2,500, using $Y_{j-1,1}$ as the instrumental variable $I_{Y_{j1}}$ in simulation scenario I.

**Figure S8:**
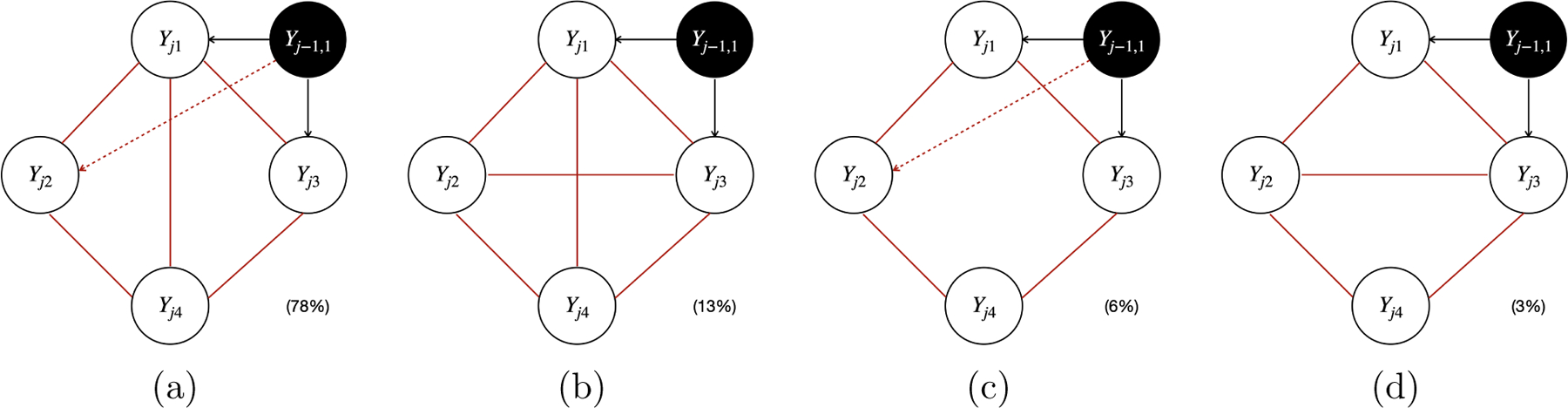
The estimated causal graphs under PCMCI^+^ with a sample size of 2,500, using $Y_{j-1,1}$ as the instrumental variable $I_{Y_{j1}}$ in simulation scenario I.

**Figure S9:**
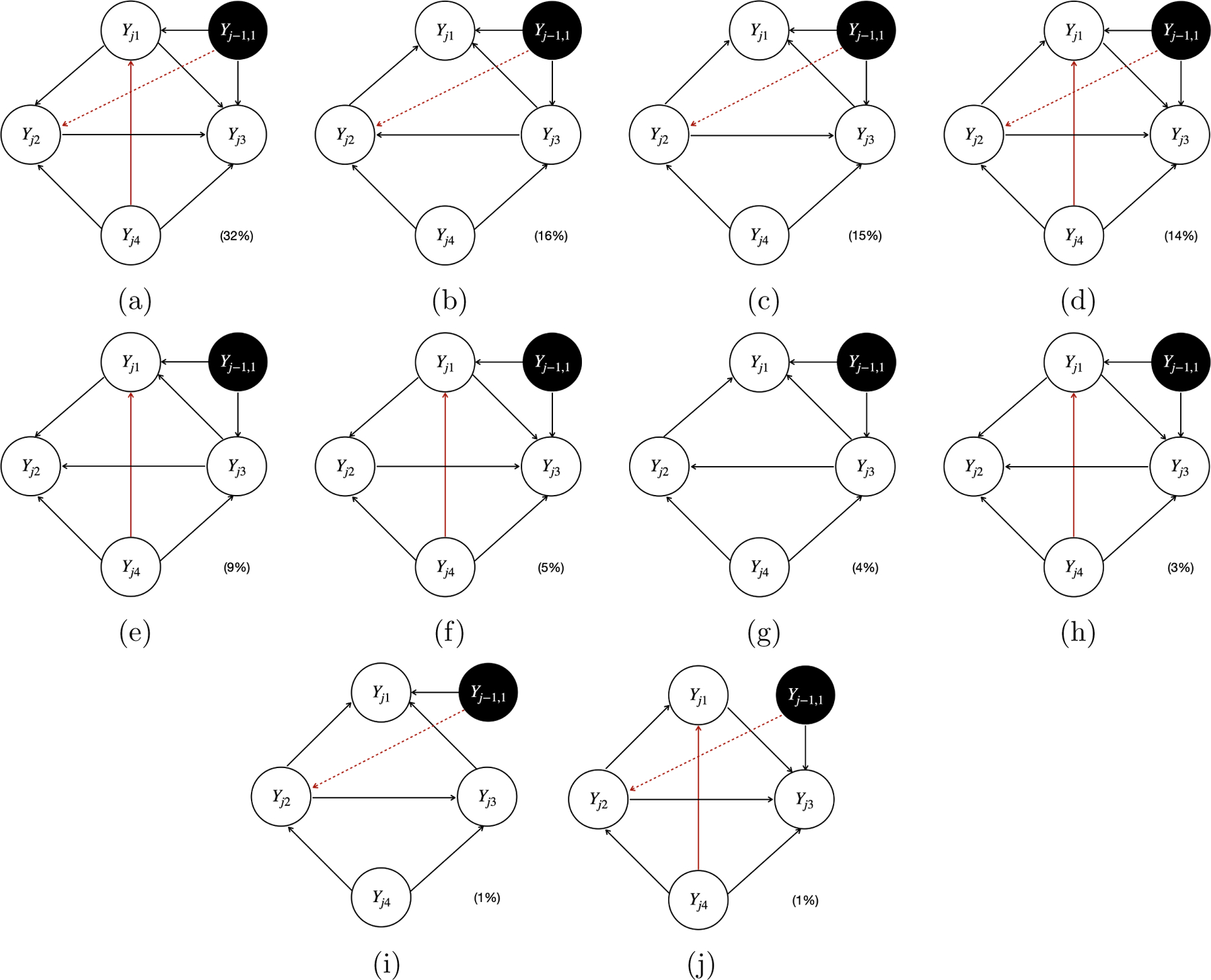
The estimated causal graphs under VAR-LiNGAM with a sample size of 2,500, using $Y_{j-1,1}$ as the instrumental variable $I_{Y_{j1}}$ in simulation scenario I.

**Figure S10:**
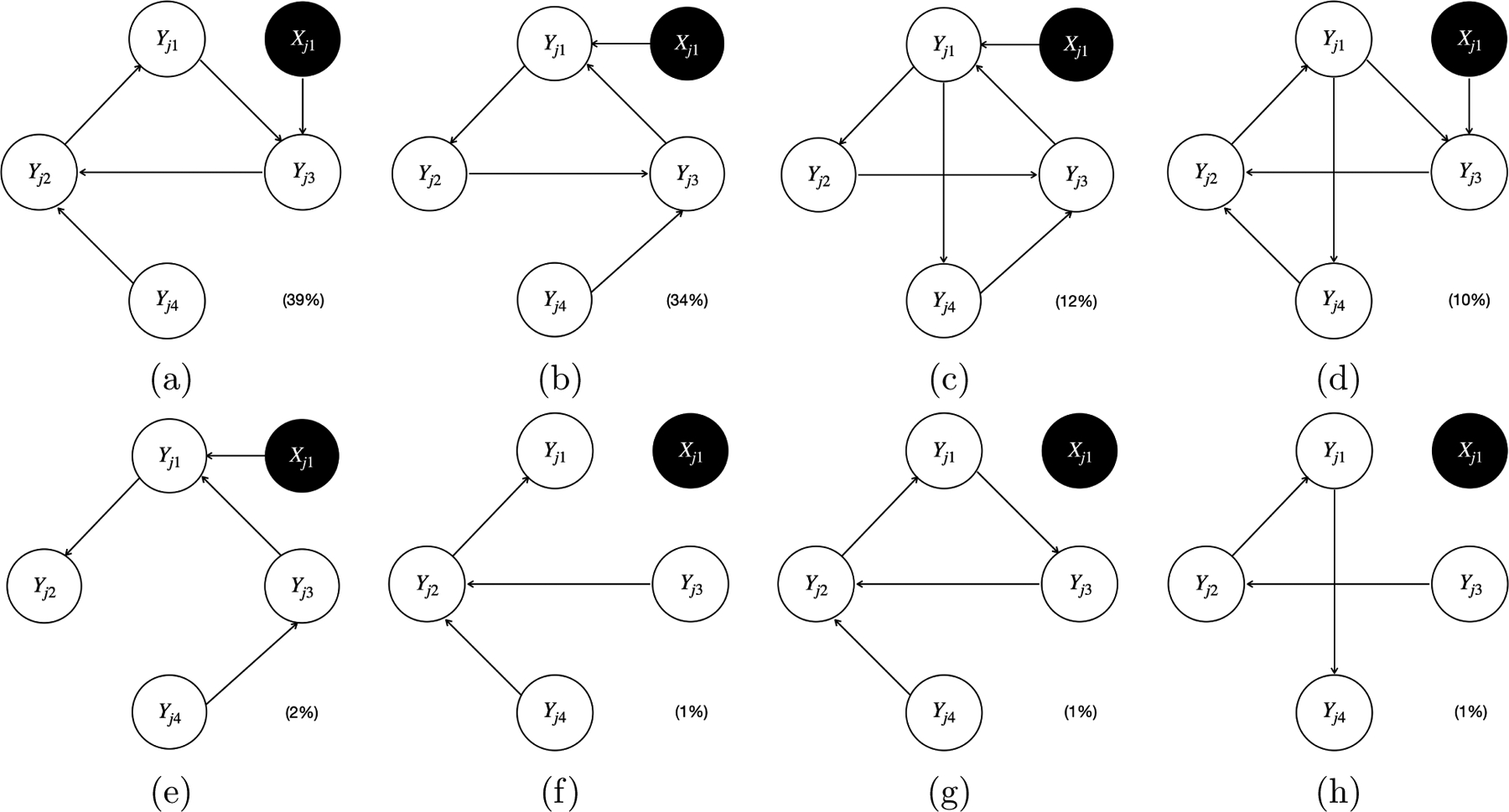
The estimated causal graphs under the proposed model with a sample size of 1,000, using $X_{j1}$ as the instrumental variable $I_{Y_{j1}}$ in simulation scenario I.

**Figure S11:**
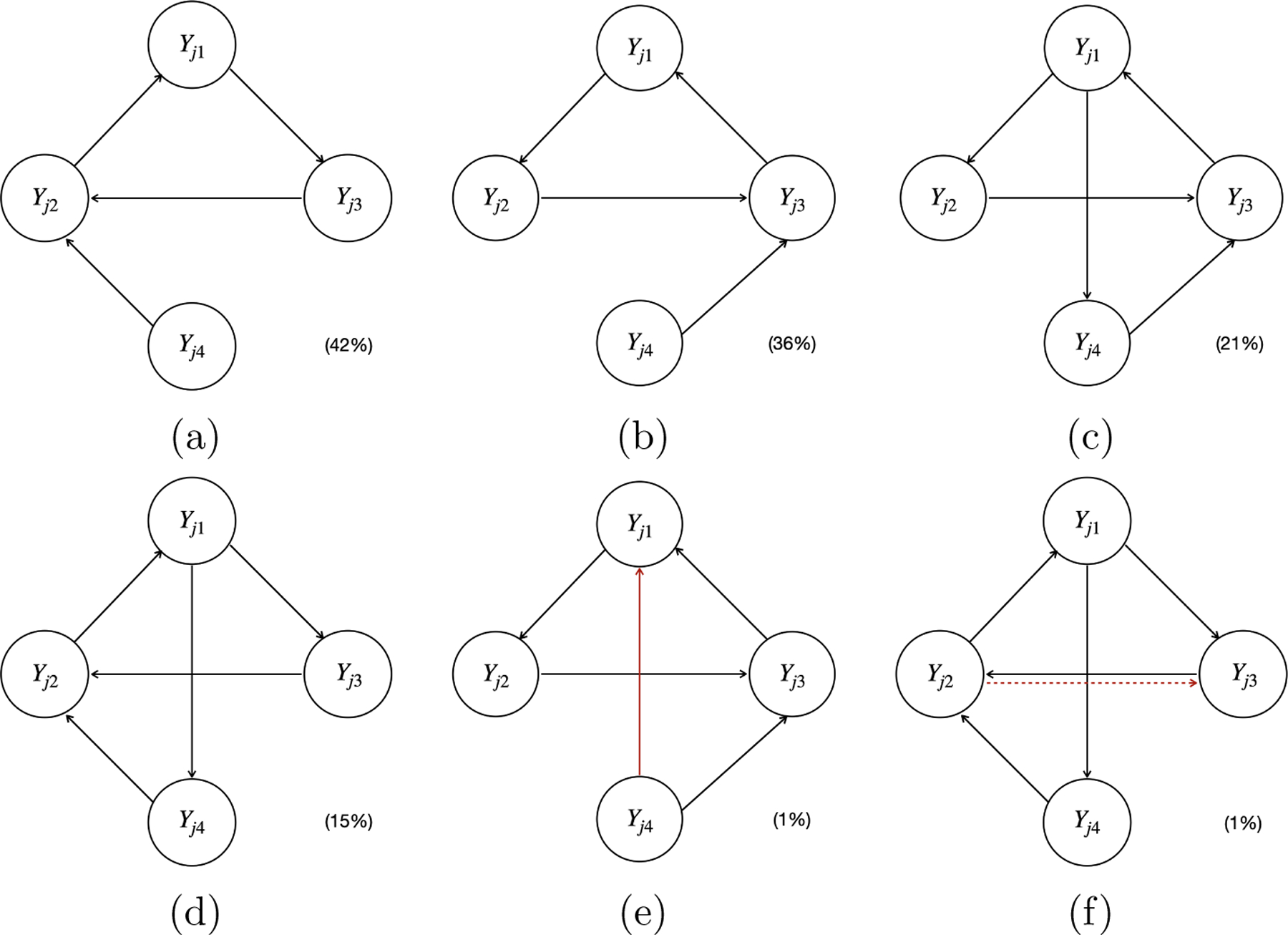
The estimated causal graphs under LiNG-D with a sample size of 1,000, using $X_{j1}$ as the instrumental variable $I_{Y_{j1}}$ in simulation scenario I.

**Figure S12:**
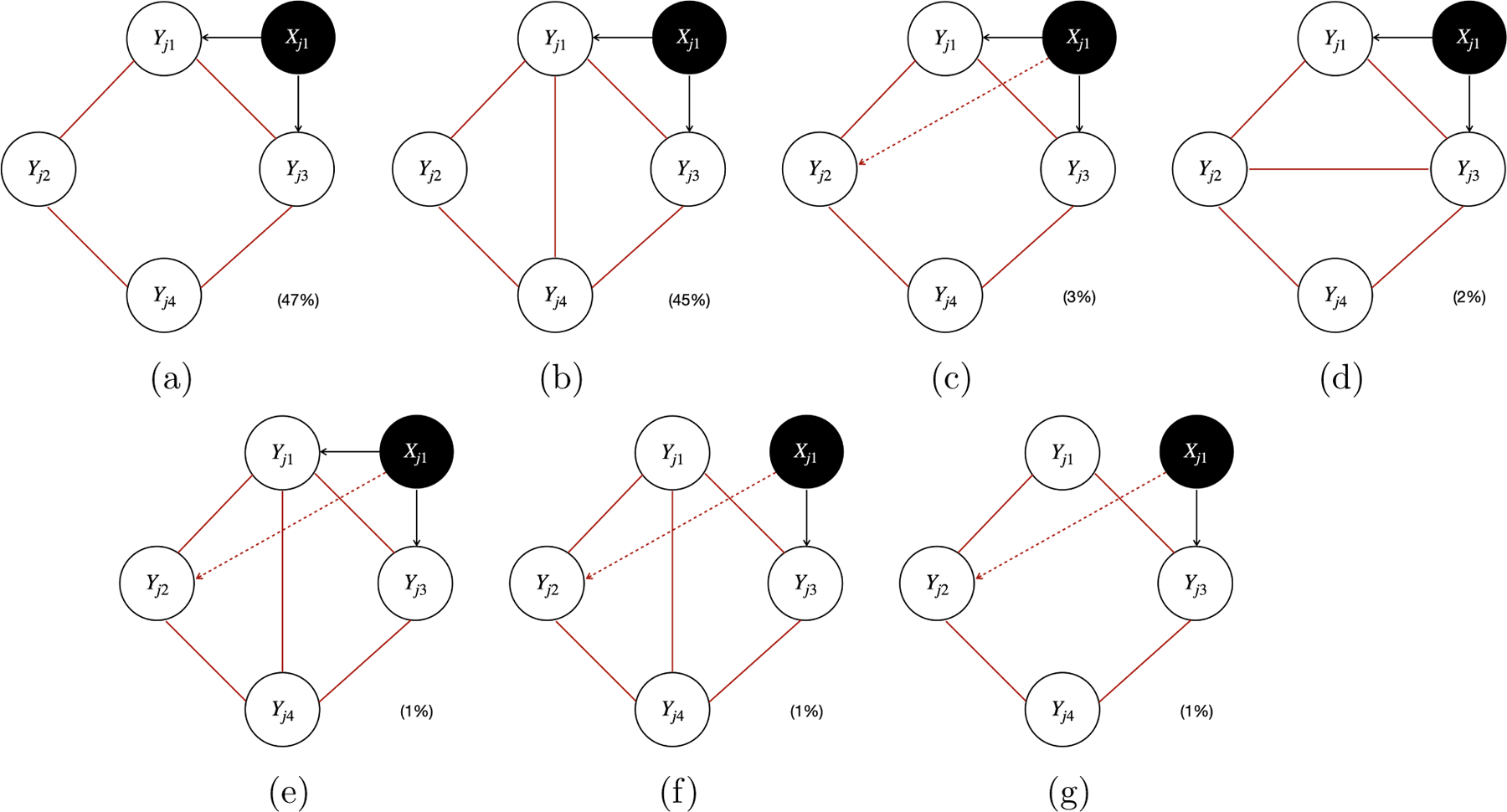
The estimated causal graphs under PCMCI^+^ with a sample size of 1,000, using $X_{j1}$ as the instrumental variable $I_{Y_{j1}}$ in simulation scenario I.

**Figure S13:**
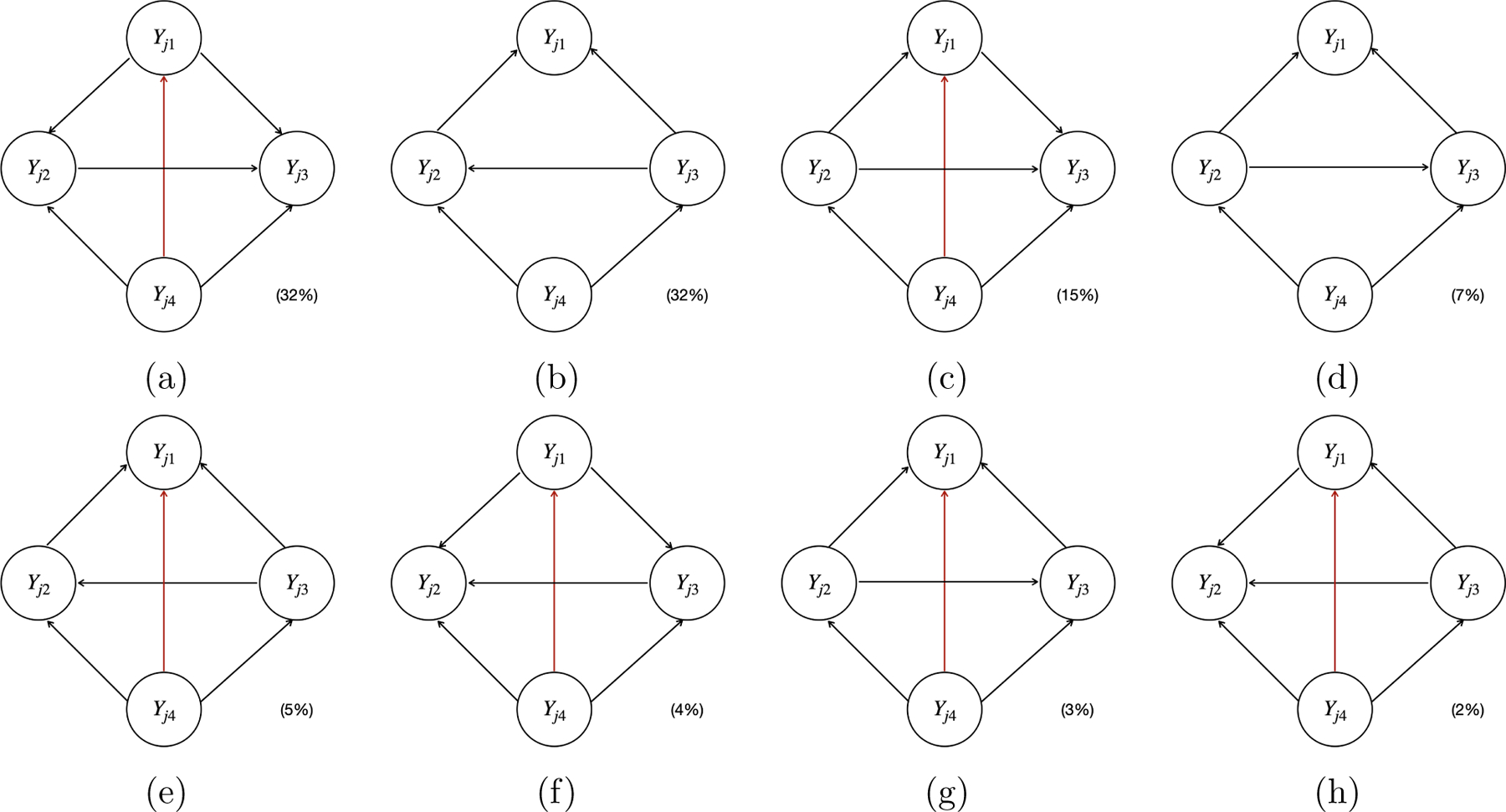
The estimated causal graphs under VAR-LiNGAM with a sample size of 1,000, using $X_{j1}$ as the instrumental variable $I_{Y_{j1}}$ in simulation scenario I.

**Figure S14:**
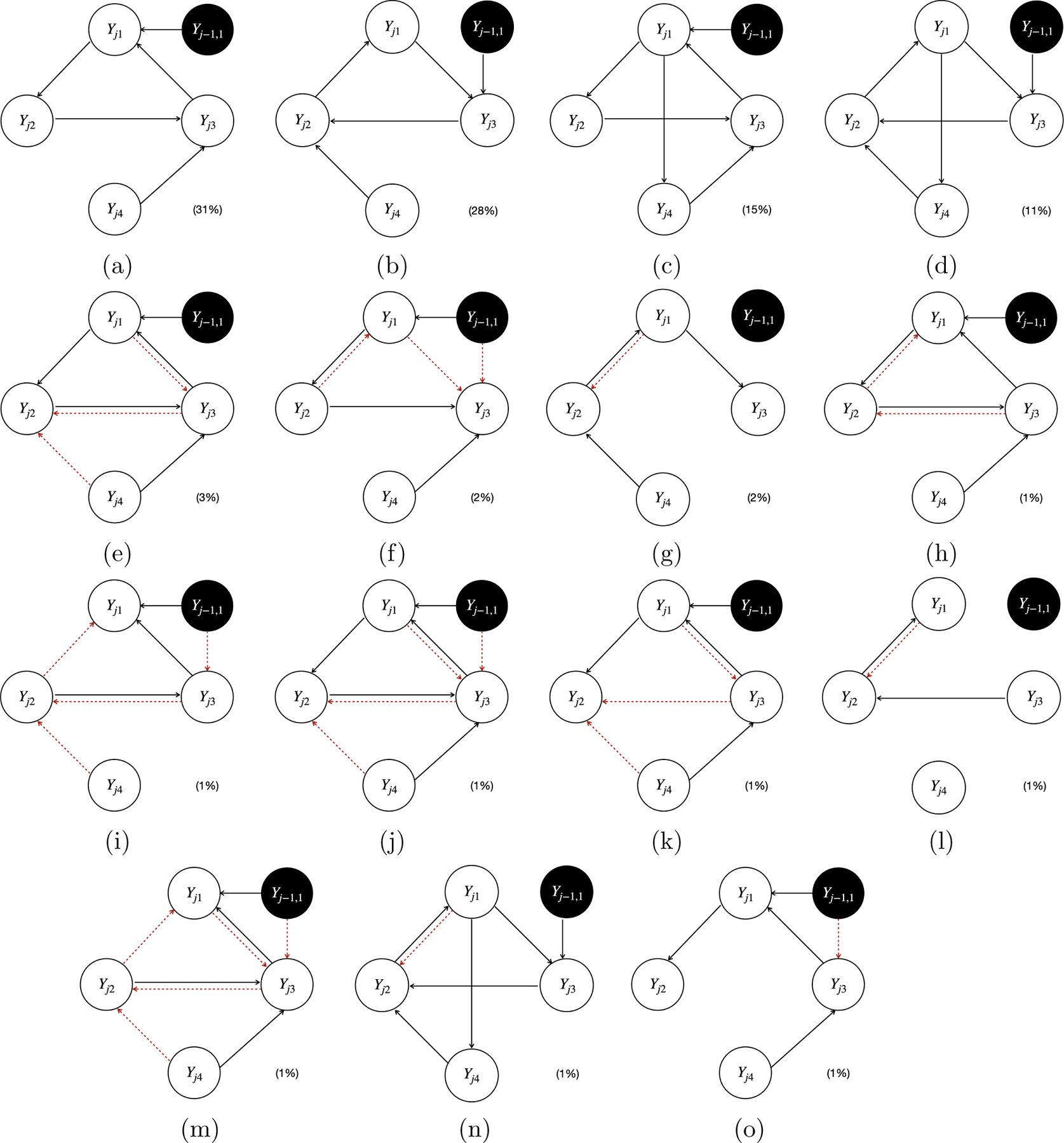
The estimated causal graphs under the proposed model with a sample size of 1,000, using $Y_{j-1,1}$ as the instrumental variable $I_{Y_{j1}}$ in simulation scenario I.

**Figure S15:**
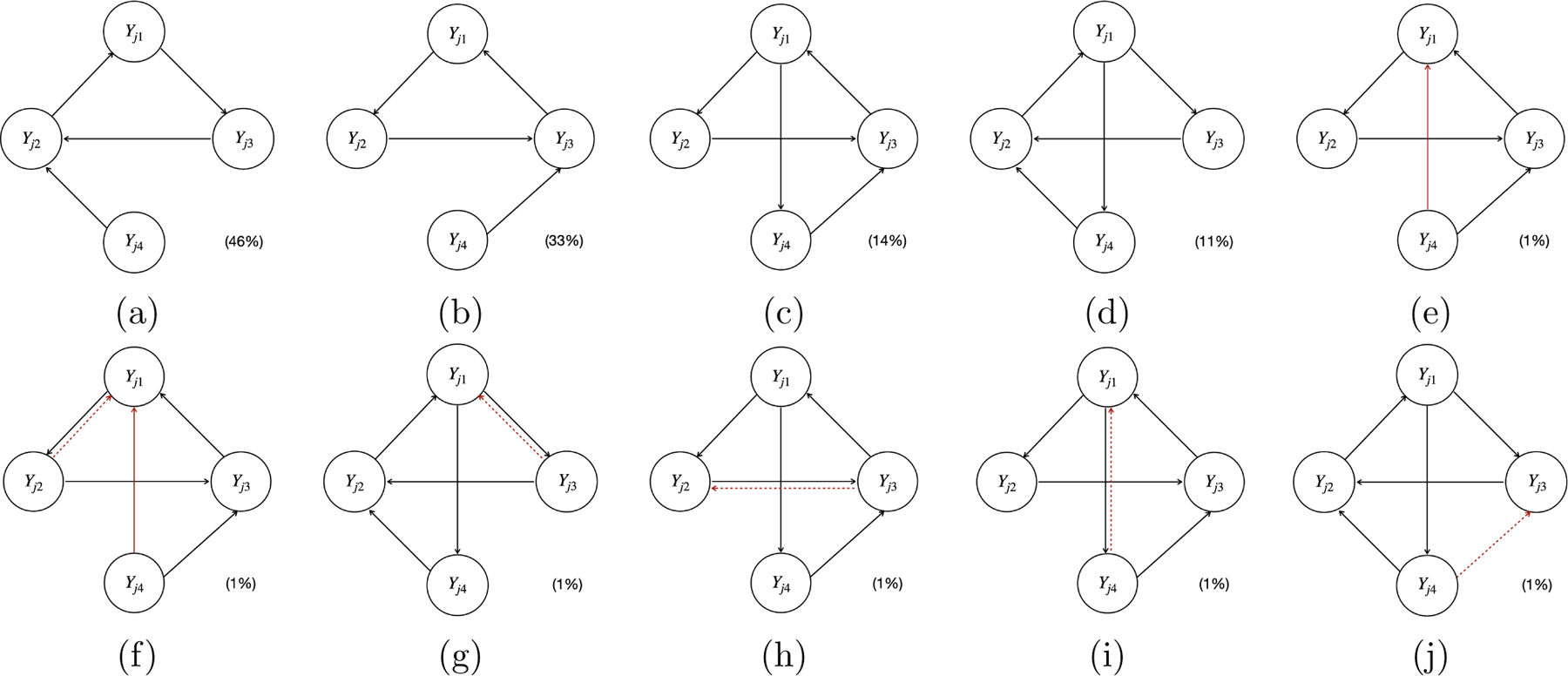
The estimated causal graphs under LiNG-D with a sample size of 1,000, using $Y_{j-1,1}$ as the instrumental variable $I_{Y_{j1}}$ in simulation scenario I.

**Figure S16:**
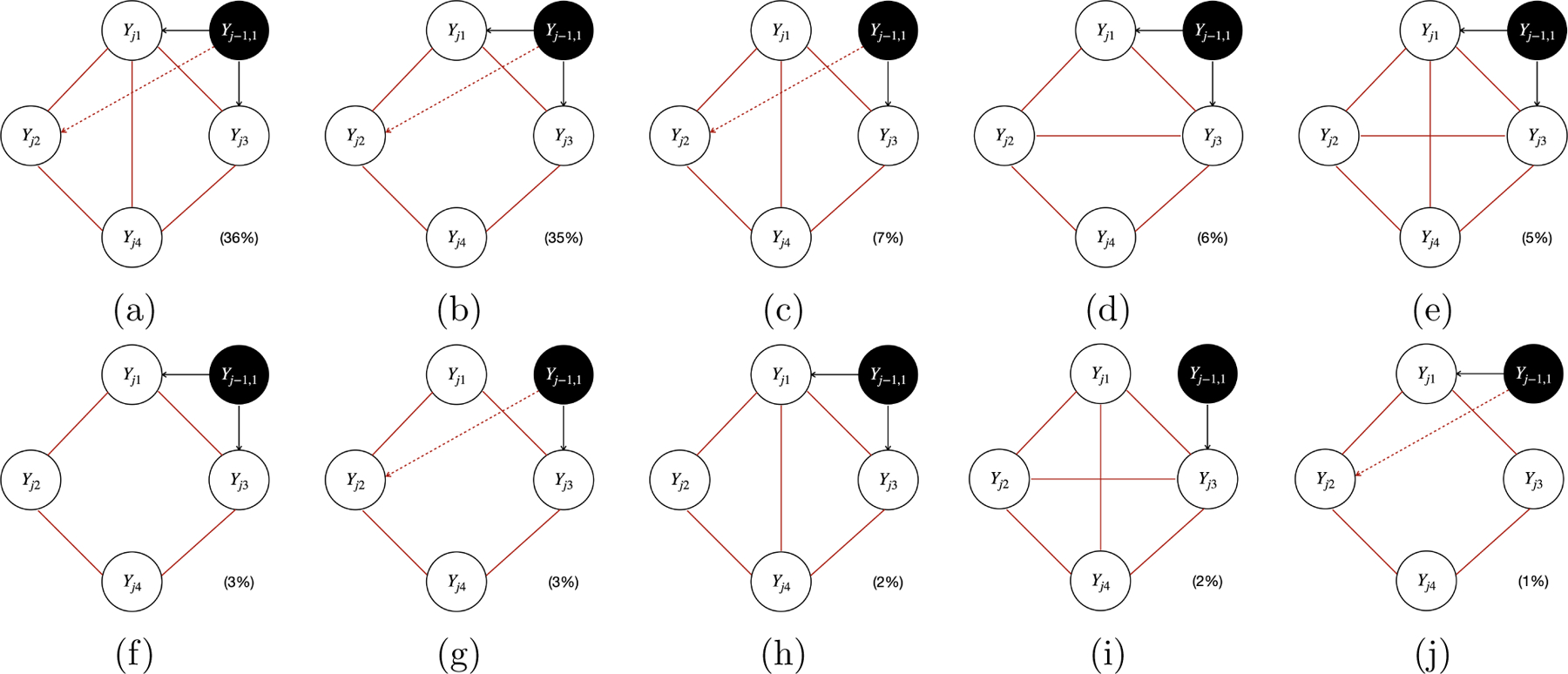
The estimated causal graphs under PCMCI^+^ with a sample size of 1,000, using $Y_{j-1,1}$ as the instrumental variable $I_{Y_{j1}}$ in simulation scenario I.

**Figure S17:**
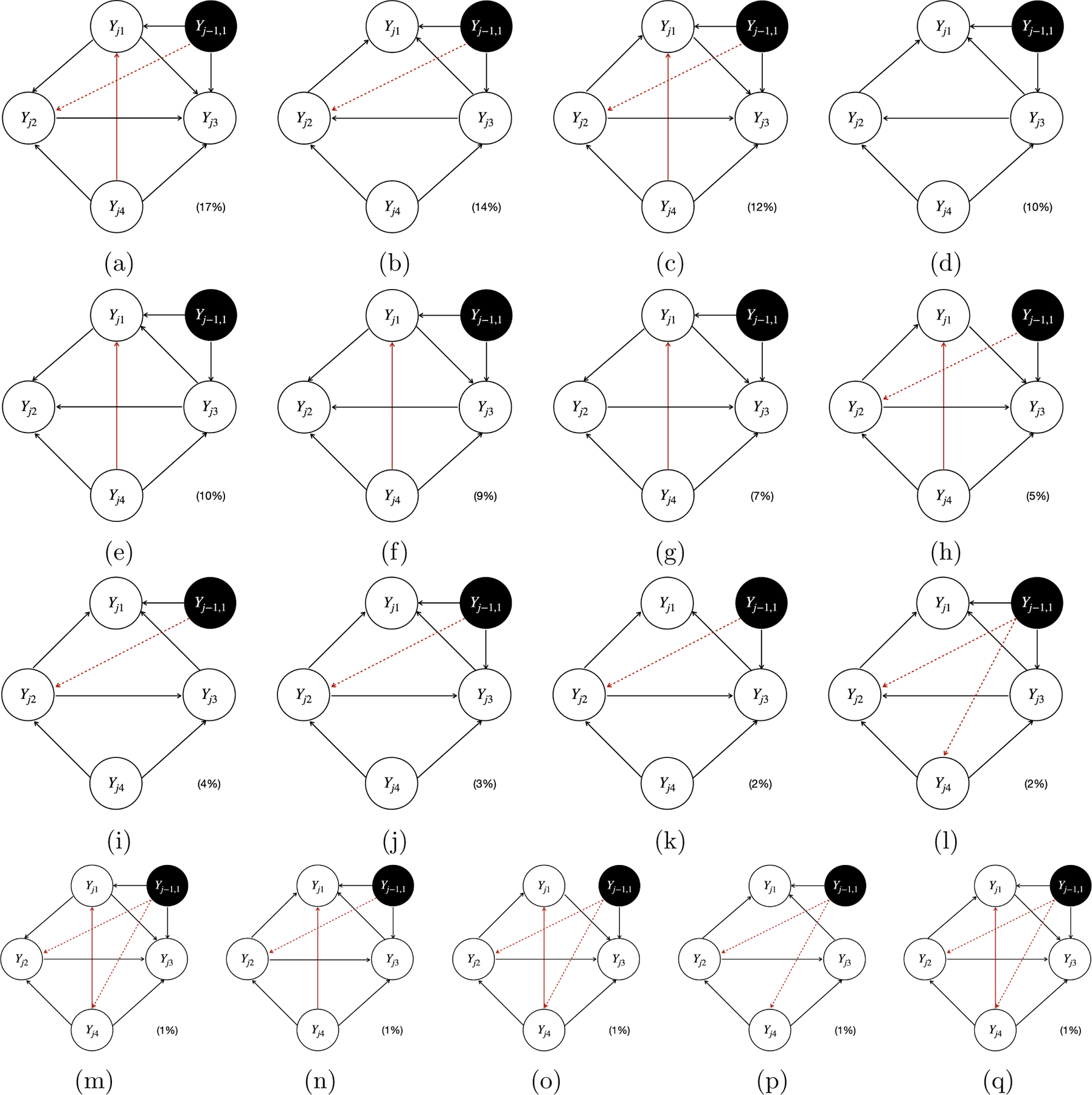
The estimated causal graphs under VAR-LiNGAM with a sample size of 1,000, using $Y_{j-1,1}$ as the instrumental variable $I_{Y_{j1}}$ in simulation scenario I.

We included the simulation results under scenario I with the sample size being 2,500 and 1,000 in Appendix Figures S2–S17. We observed similar simulation results for identifying the ICA equivalence classes as those obtained when the sample size was 5,000. To compare the performance of all methods in detecting individual edges, we reported their marginal detection probability, defined as the relative frequency of detecting a specific edge, summing over all individual causal graphs across 100 replications. As the sample size increased, the estimation performance of the proposed model, LiNG-D, and PCMCI^+^ improved. For example, when using $X_{j1}$ as the instrument variable for $Y_{j1}$, the probability of detecting the edge between $Y_{j1}$ and $Y_{j4}$ was 23% = 12% + 10% + 1%, 37% = 21% + 15% + 1%, and 47% = 45% + 1% + 1% under the proposed model, LiNG-D, and PCMCI^+^, respectively, with a sample size of 1,000; however, the corresponding probability was 98% = 52% + 46%, 96% = 53% + 43%, and 100% = 83% + 10% + 7% when sample size was 5,000.

The proposed model did not uniformly outperform alternative methods. For example, when using $Y_{j-1,1}$ as the instrumental variable for $Y_{j1}$ with a sample size of 1,000, 2,500, and 5,000, the probability of detecting the edge between $Y_{j1}$ and $Y_{j4}$ under the proposed model was 27% = 15% + 11% + 1%, 72% = 41% + 30% + 1%, and 95% = 48% + 45% + 2%, respectively, whereas PCMCI^+^ achieved a true positive rate of 52% = 36% + 7% + 5% + 2% + 2%, 91% = 78% + 13%, and 100% = 81% + 17% + 2%, respectively. When compared to LiNG-D, the proposed model demonstrated a higher false positive rate when using $Y_{j-1,1}$ as the instrumental variable with a sample size of 1,000 (see Appendix Figures S14–S15).

Lastly, to further showcase the ability of the proposed Bayesian learning algorithm to robustly and accurately identify ICA equivalence classes, we conducted additional simulation studies using a different simulated true graph from the one discussed in this Section. Detailed simulation results, highlighting the superior performance of our method compared to alternative methods (i.e., LiNG-D, VAR-LiNGAM, and PCMCI^+^), are provided in Appendix C.1. To empirically verify the causal identification theory under the proposed model with a varying number of visits $J_{i}$, we conducted additional simulation studies. Detailed descriptions of the setups and results, demonstrating that our causal identification theory applies effectively to general cases with varying $J_{i}$, are provided in Appendix C.2.

### 5.2 Simulation Scenario II

**Figure 10:**
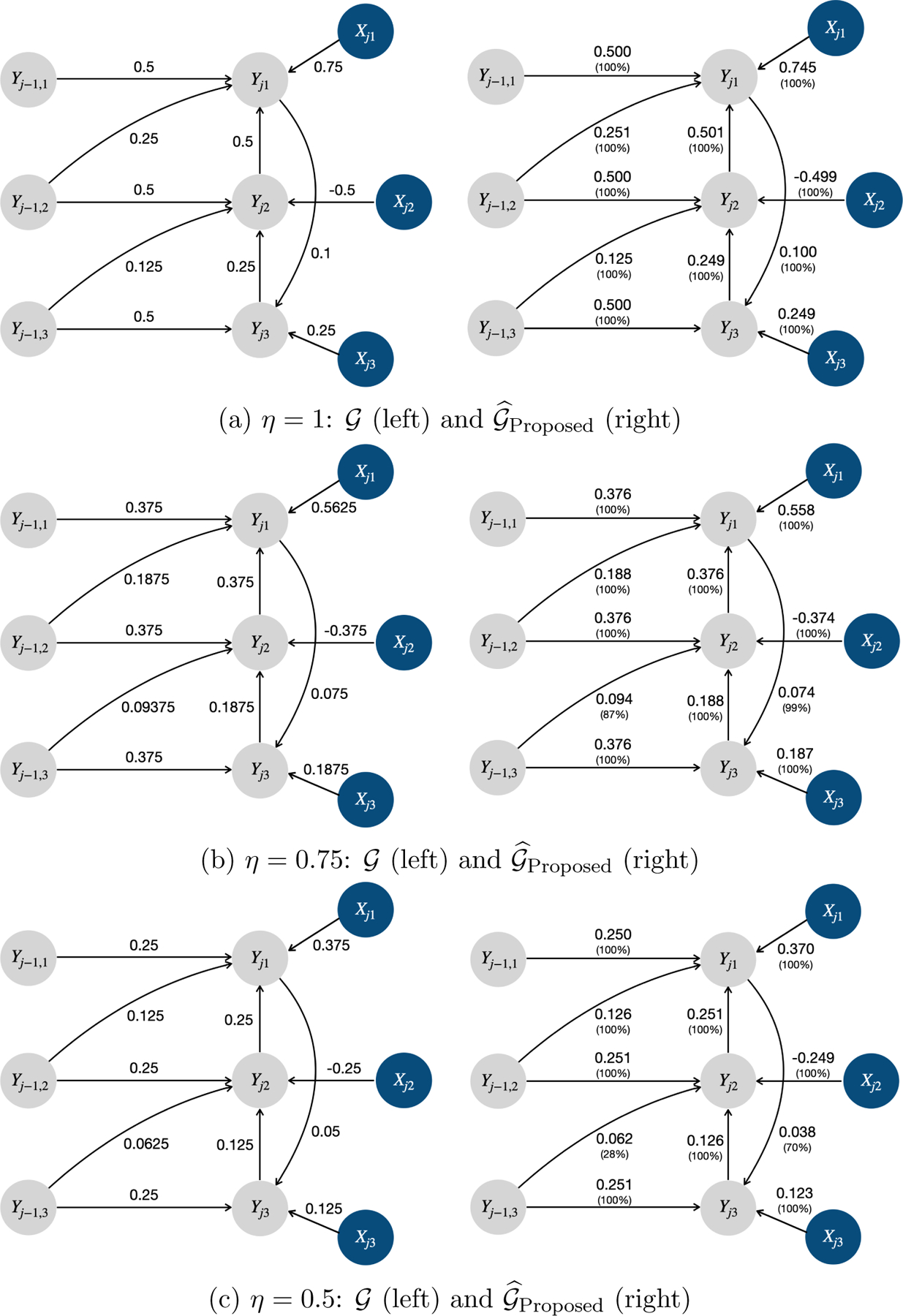
Simulated true causal graphs, and the estimated causal graphs under the proposed model in scenario II with η={0.5,0.75,1}. The grey and blue circles represent the longitudinal health outcomes and covariates, respectively.

In this scenario, we evaluated the performance of the proposed model by generating synthetic datasets that mimic our motivating HIV dataset. Assume that there were 200 individuals with Q=3 longitudinal health outcomes and S=3 covariates including one binary covariate and two continuous covariates, i.e., $X_{ij}=\left(X_{ij1},X_{ij2},X_{ij3}\right)$, where $X_{ij1}$’s were generated from Bernoulli(0.6), and $X_{ij2}$, $X_{ij3}$’s were generated from independent standard normal distributions, i=1,…,200. The number of longitudinal observations $J_{i}$ for each individual i was randomly sampled from our motivating HIV dataset without replacement, resulting in the number ranging from 3 to 46, and a total sample size of 3, 684. We set the simulated true values for the intercepts to be $\mu_{1}=1$, $\mu_{2}=-1$, $\mu_{3}=0$, and the variances of the Laplace errors to be 1, yielding $\sigma_{q}^{2}=1/8$ for q=1,…,Q. Then we generated the simulated true $Y_{ijq}$ using the following data-generating process,

(6) $$\left\{\begin{array}{l} Y_{ij1}=\mu_{1}+0.5\eta \times Y_{ij2}+0.5\eta \times Y_{i,j-1,1}+0.25\eta \times Y_{i,j-1,2}+0.75\eta \times X_{ij1}+e_{ij1},\\ Y_{ij2}=\mu_{2}+0.25\eta \times Y_{ij3}+0.5\eta \times Y_{i,j-1,2}+0.125\eta \times Y_{i,j-1,3}-0.5\eta \times X_{ij2}+e_{ij2},\\ Y_{ij3}=\mu_{3}+0.1\eta \times Y_{ij1}+0.5\eta \times Y_{i,j-1,3}+0.25\eta \times X_{ij3}+e_{ij3}, \end{array}\right.$$

where η∈{0.5,0.75,1} controls the causal effect size. The left panels of Figure 10(a,b,c) plot the simulated true causal graph 𝒢 with η=1, 0.75, and 0.5. The true number of time lags are $L_{y}^{\text{truth}}=1$ and $L_{x}^{\text{truth}}=0$ for longitudinal health outcomes and covariates, respectively. Note that $X_{j1}$ and $Y_{j-1,1}$ are instrumental variables for $Y_{j1}$, and $X_{j2}$ and $X_{j3}$ are instrumental variables for $Y_{j2}$ and $Y_{j3}$, respectively.

We applied the proposed Bayesian structural learning algorithm to the simulated datasets with the following hyper-parameter values: $\nu_{0}=5e-5\times \eta$ for η∈{0.5,0.75,1}, $a_{\nu }=5$, $b_{\nu }=50$, $a_{\rho }=b_{\rho }=0.5$, $\sigma_{\mu }^{2}=100$, and $a_{\sigma }=b_{\sigma }=1$. Considering the simulated true graph 𝒢 in this scenario with a substantial number of time-lagged and instantaneous causal relationships, we determined the number of time lags $L_{y}$ and $L_{x}$ in the proposed model from the data following the idea from Pamfil et al. (2020). They observed a significant decrease in the largest absolute value of the estimated coefficients $B_{\ell }$ toward zero as ℓ increased from $L_{y}^{\text{truth}}$ to $L_{y}^{\text{truth}}+1$. This phenomenon provided a basis for determining the number of time lags $L_{y}$. The same rule was applied to select $L_{x}$ based on $A_{\ell }$. We performed 100 repeated experiments for each configuration of the simulated dataset with the same MCMC setup as in scenario I. To determine the presence of a directed edge in the estimated causal graph, we used the same median probability model criteria as in scenario I.

For comparison, we again applied PCMCI^+^, LiNG-D, and VAR-LiNGAM using the same setup as in scenario I. In addition, to illustrate the advantages of the proposed model in capturing both time-lagged and instantaneous causal relationships, we considered two special cases of the proposed model and the learning algorithm for comparison. The first model is the (cross-sectional) structural causal model (SCM, Bollen 1989), which only estimates the instantaneous causal effects by setting the time-lagged causal effects to zero in the proposed model. The second model is the vector autoregressive model (VAR, Swanson and Granger 1997), which solely estimates the time-lagged causal effects by fixing the instantaneous causal effects at zero in the proposed model.

**Figure S18:**
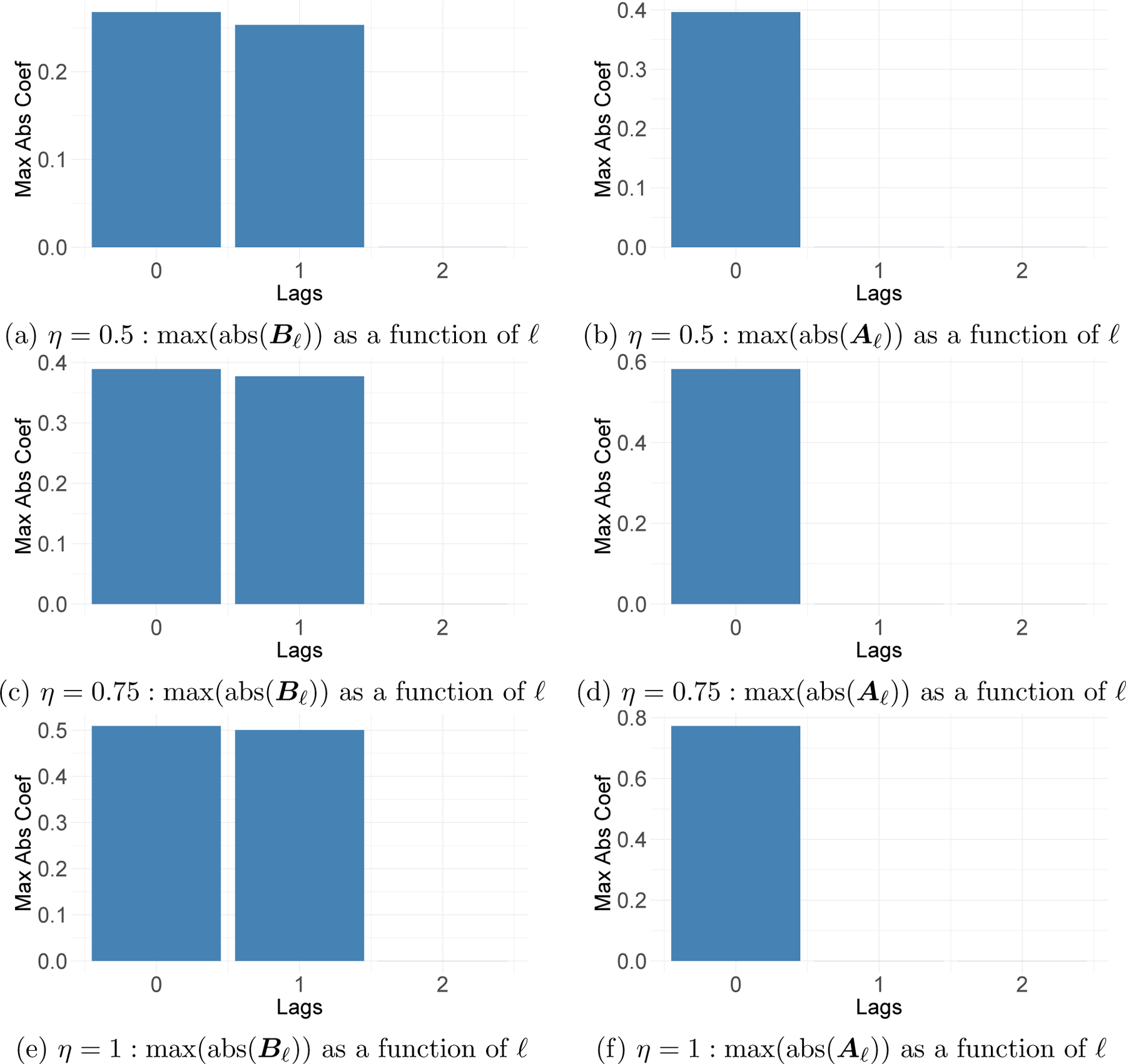
Largest absolute values of the estimated coefficients $B_{\ell }$ and $A_{\ell }$ for one randomly selected simulated dataset from 100 experiments in the simulation scenario II. The simulated true number of time lags are $L_{y}^{\text{truth}}=1$ and $L_{x}^{\text{truth}}=0$. To select the number of lags, note that $\text{max}\left(\text{abs}\left(B_{\ell }\right)\right)$ and $\text{max}\left(\text{abs}\left(A_{\ell }\right)\right)$ decrease significantly towards zero from $\ell =L_{y}^{\text{truth}}$ to $\ell =L_{y}^{\text{truth}}+1$, and from $\ell =L_{x}^{\text{truth}}$ to $\ell =L_{x}^{\text{truth}}+1$, respectively.

We now report the simulation results for scenario II. As shown in Appendix Figure S18, the proposed model successfully identified $L_{y}=1$ and $L_{x}=0$. Figure 10(a,b,c) summarize the estimated causal graphs under the proposed model when η=1, 0.75, and 0.5, including the estimated causal effects averaged over 100 replications and the probabilities for detecting the corresponding edges. We found that the proposed model not only perfectly recovered the simulated true causal graph 𝒢, but also estimated both the time-lagged and instantaneous causal effects with high accuracy.

**Figure 11:**
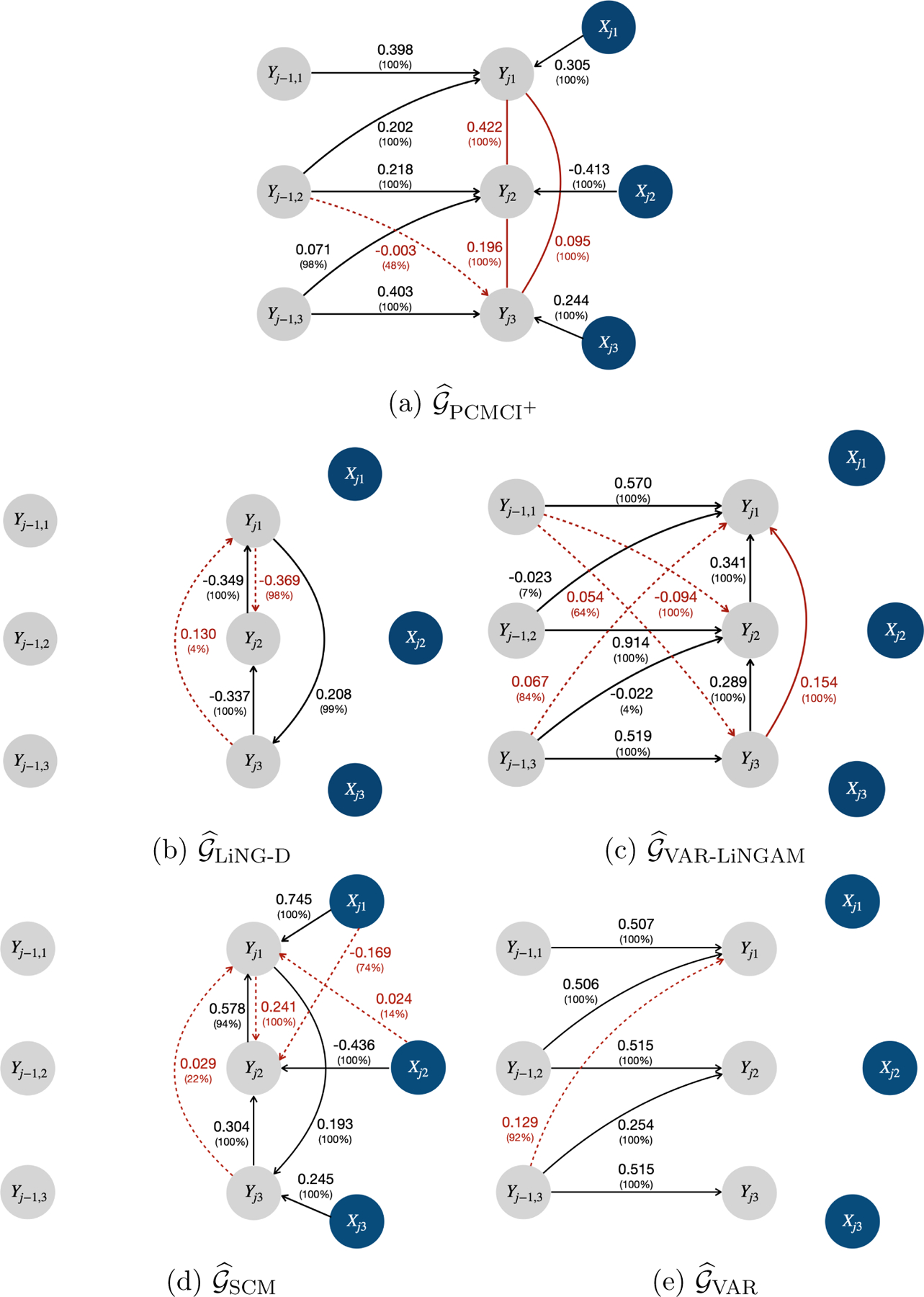
The estimated causal graphs under alternative methods (i.e., PCMCI^+^, LiNG-D, VAR-LiNGAM, SCM, and VAR) in scenario II with η=1. The grey and blue circles represent the longitudinal health outcomes and covariates, respectively. The solid red lines indicate the unoriented causal links. The solid and dashed red arrows indicate reversed and spurious causal links, respectively.

Moreover, Figure 11 displays the estimated causal graphs under the alternative methods PCMCI^+^, LiNG-D, VAR-LiNGAM, SCM, and VAR when η=1. Due to relying on the assumption of acyclicity, PCMCI^+^ detected all directed edges in the instantaneous directed cycle $Y_{j1}\leftarrow Y_{j2}\leftarrow Y_{j3}\leftarrow Y_{j1}$ without orientation, and VAR-LiNGAM reversed the direction of the edge $Y_{j1}\to Y_{j3}$ to avoid the formation of cycles. While LiNG-D successfully identified the instantaneous cycle, it did not capture any time-lagged causal relationships. In addition, all the alternative methods detected spurious causal links. Therefore, the proposed model outperformed all the alternatives in terms of recovering the underlying causal structure.

**Figure S19:**
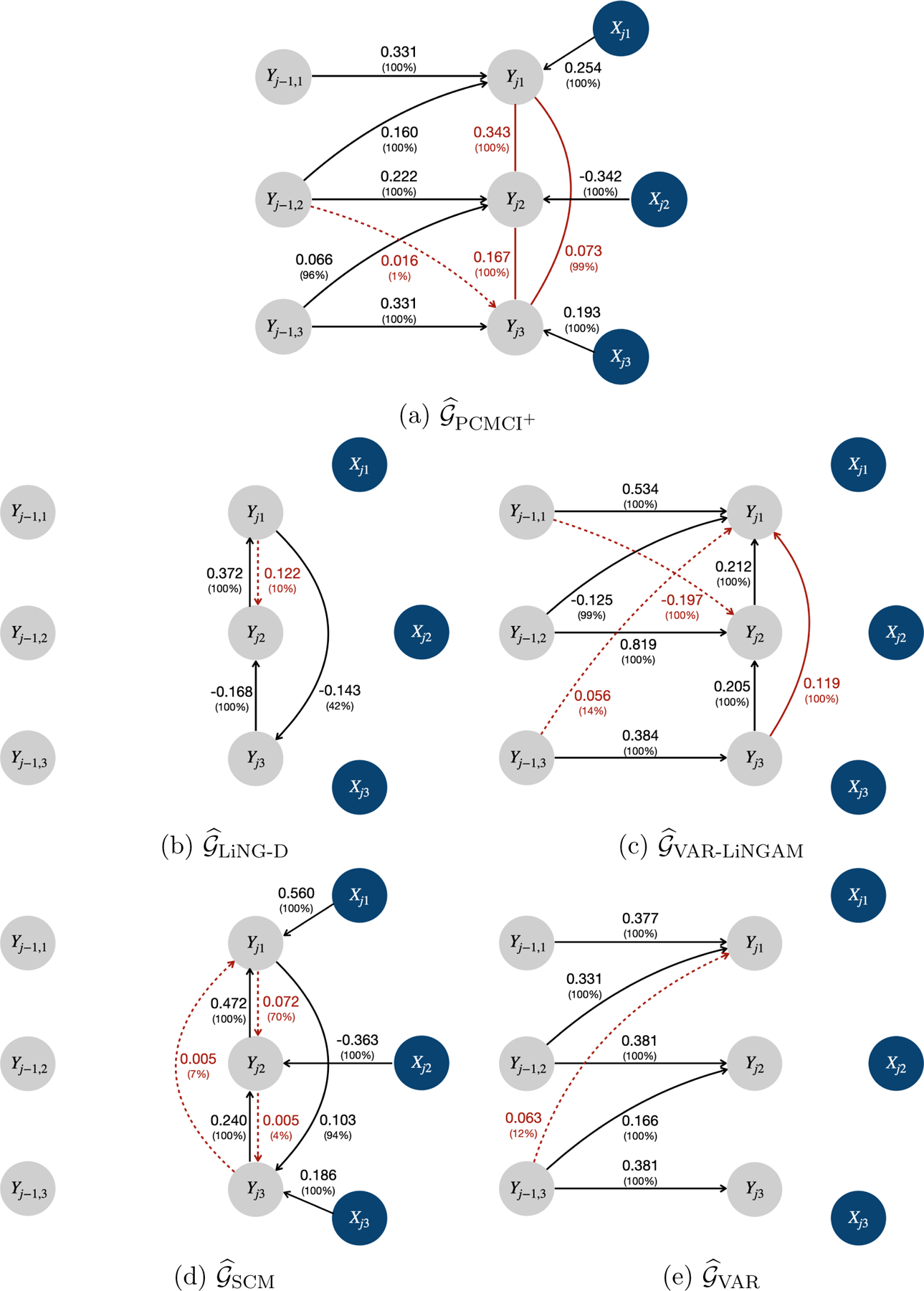
The estimated causal graphs under alternative methods (PCMCI^+^, LiNG-D, VAR-LiNGAM, SCM, and VAR) in scenario II with η=0.75.

**Figure S20:**
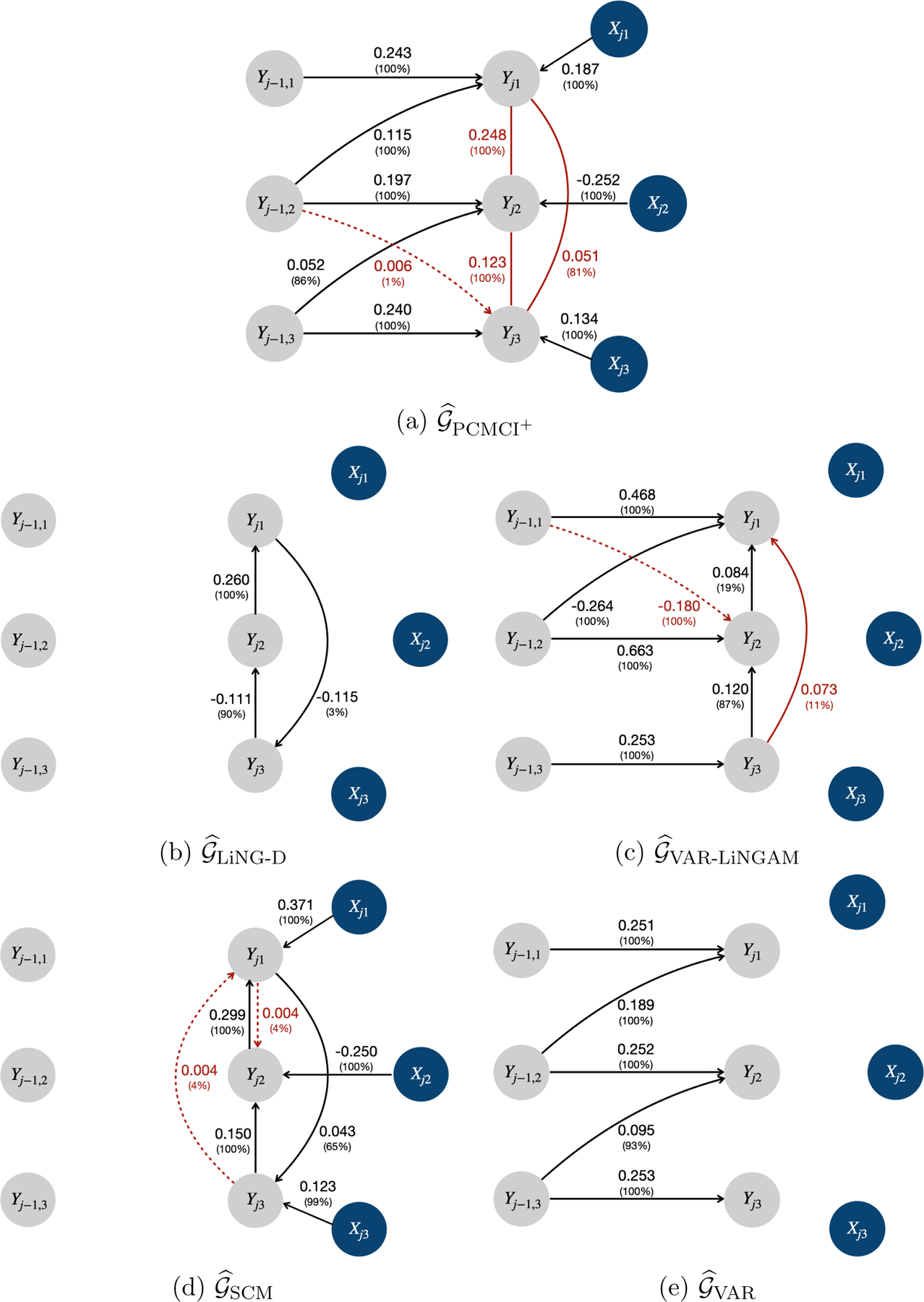
The estimated causal graphs under alternative methods (PCMCI^+^, LiNG-D, VAR-LiNGAM, SCM, and VAR) in scenario II with η=0.5.

Lastly, we provide simulation results for alternative methods under scenario II with the causal effect size being η=0.75 and 0.5 in Appendix Figure S19 and S20, respectively. When the effect size was small, all methods exhibited a lower true positive rate for detecting edges with small causal effects. For example, the probability of detecting the edge between $Y_{j1}$ and $Y_{j3}$ was 70%, 81%, 3%, 11%, and 65% under ${\hat{𝒢}}_{\text{Proposed}}$, ${\hat{𝒢}}_{{\text{PCMCI}}^{+}}$, ${\hat{𝒢}}_{\text{LiNG-D}}$, ${\hat{𝒢}}_{\text{VAR-LiNGAM}}$ and ${\hat{𝒢}}_{\text{SCM}}$ with η=0.5, respectively; while the probability of detecting the edge between $Y_{j1}$ and $Y_{j3}$ was almost 100% for all the methods with η=1. Note that when the effect size was small, PCMCI^+^ had a slightly higher true positive rate than the proposed model. In addition, all the alternative methods exhibited a lower false positive rate with a small effect size. For instance, the probability of detecting $Y_{j-1,2}\to Y_{j3}$ was 48%, 1%, and 1% by ${\hat{𝒢}}_{{\text{PCMCI}}^{+}}$ when η=1, 0.75 and 0.5, respectively; the probability of detecting $Y_{j1}\to Y_{j2}$ was 98%, 10%, and 0% by ${\overset{ˆ}{𝒢}}_{\text{LiNG-D}}$ when η=1, 0.75 and 0.5, respectively; the probability of detecting $Y_{j-1,3}\to Y_{j1}$ was 84%, 14%, and 0% by ${\hat{𝒢}}_{\text{VAR-LiNGAM}}$ when η=1, 0.75 and 0.5, respectively; the probability of detecting $Y_{j1}\to Y_{j2}$ was 100%, 70%, and 4% by ${\hat{𝒢}}_{\text{SCM}}$ when η=1, 0.75 and 0.5, respectively; the relative frequency of detecting $Y_{j-1,3}\to Y_{j1}$ was 92%, 12%, and 0% by ${\hat{𝒢}}_{\text{VAR}}$ when η=1, 0.75 and 0.5, respectively.

## 6. Application: WIHS Data Analysis

The Women’s Interagency HIV Study (WIHS, Adimora et al. 2018) is a large prospective, observational, multicenter study designed to investigate the impact of HIV infection on multimorbidity in women with HIV or at risk for HIV in the United States. Semi-annually, participants follow up with their physicians for data collection, including assessments of sociodemographic, clinical, and behavioral characteristics. For the present analysis, we included all women from the Washington, D.C. site with at least two visits, yielding a total of 298 individuals. We were interested in investigating the causal relationships among the following Q=8 longitudinal health outcomes that HIV physicians commonly account for when making treatment decisions in clinical practice: depression scores evaluated through the Center for Epidemiological Studies Depression Scale (Radloff, 1977) spanning somatic symptoms (e.g., sleep and appetite difficulties), negative affect (e.g., loneliness and sadness), lack of positive affect (e.g., hopelessness), and interpersonal symptoms (e.g., people are unfriendly); viral load of HIV RNA, CD4 count, estimated glomerular filtration rate (eGFR; a kidney function indicator), and body mass index (BMI). To verify the assumption of non-Gaussian noise, we conducted the Shapiro–Wilk test (Shaphiro and Wilk, 1965), where all the health outcomes rejected the null hypothesis. We also extracted risk factors including age, race, diabetes, smoking status, marital status, and education level as covariates.

**Figure S21:**
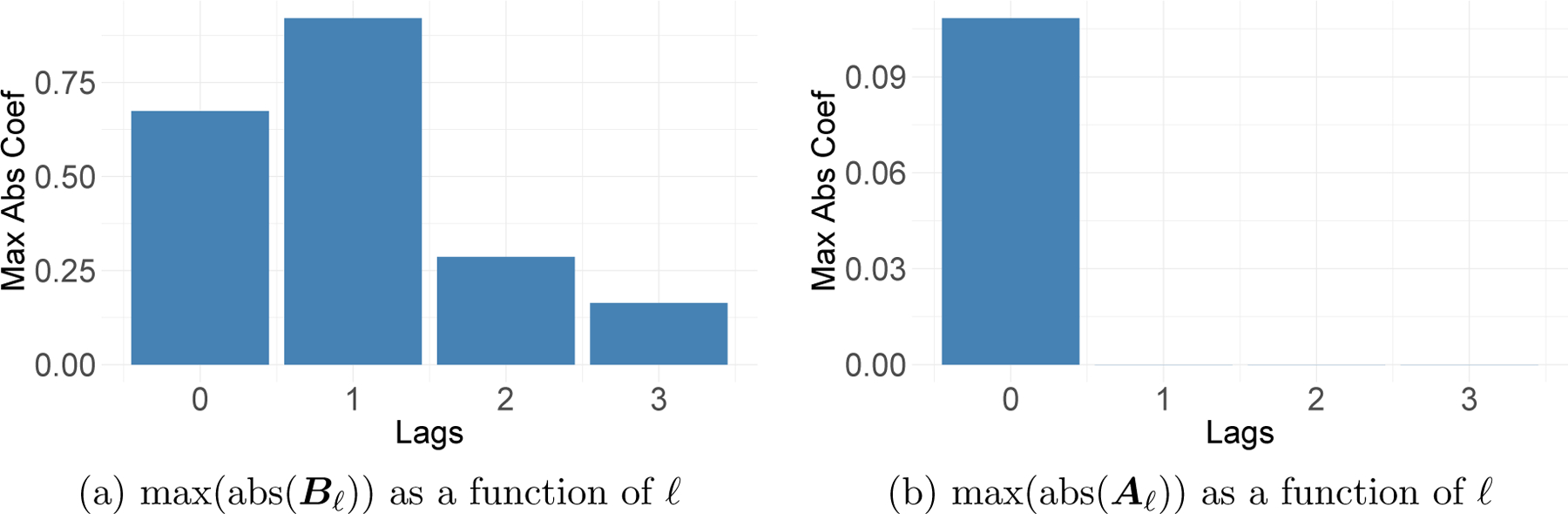
Largest absolute values of the estimated coefficients $B_{\ell }$ and $A_{\ell }$ in the WIHS data analysis. The number of time lags were selected to be $L_{y}=1$ and $L_{x}=0$, since $\text{max}\left(\text{abs}\left(B_{\ell }\right)\right)$ and $\text{max}\left(\text{abs}\left(A_{\ell }\right)\right)$ decrease significantly towards zero from $\ell =L_{y}$ to $\ell =L_{y}+1$, and from $\ell =L_{x}$ to $\ell =L_{x}+1$, respectively.

We applied the proposed model to the WIHS dataset. We slightly modified the spike-and-slab prior on $\beta_{\ell qp}$ by assuming that (1) $\beta_{\ell qp}=0,\ell >0$ if $\beta_{0qp}=0$; and (2) sign($\beta_{\ell qp}$) is the same for all ℓ≥0 for incorporating prior clinical knowledge and better interpretation. The same modification was applied to $\alpha_{\ell qs}$. The details of the prior specification are described in Appendix D. In addition, we selected the number of time lags using the same criteria as in the simulation study. As shown in Appendix Figure S21, $L_{y}=1$ and $L_{x}=0$. We ran 25,000 MCMC iterations after an initial burn-in of 25,000 iterations, and a thinning factor of 50. We also applied PCMCI^+^ to the same WIHS dataset for comparison.

**Figure 12:**
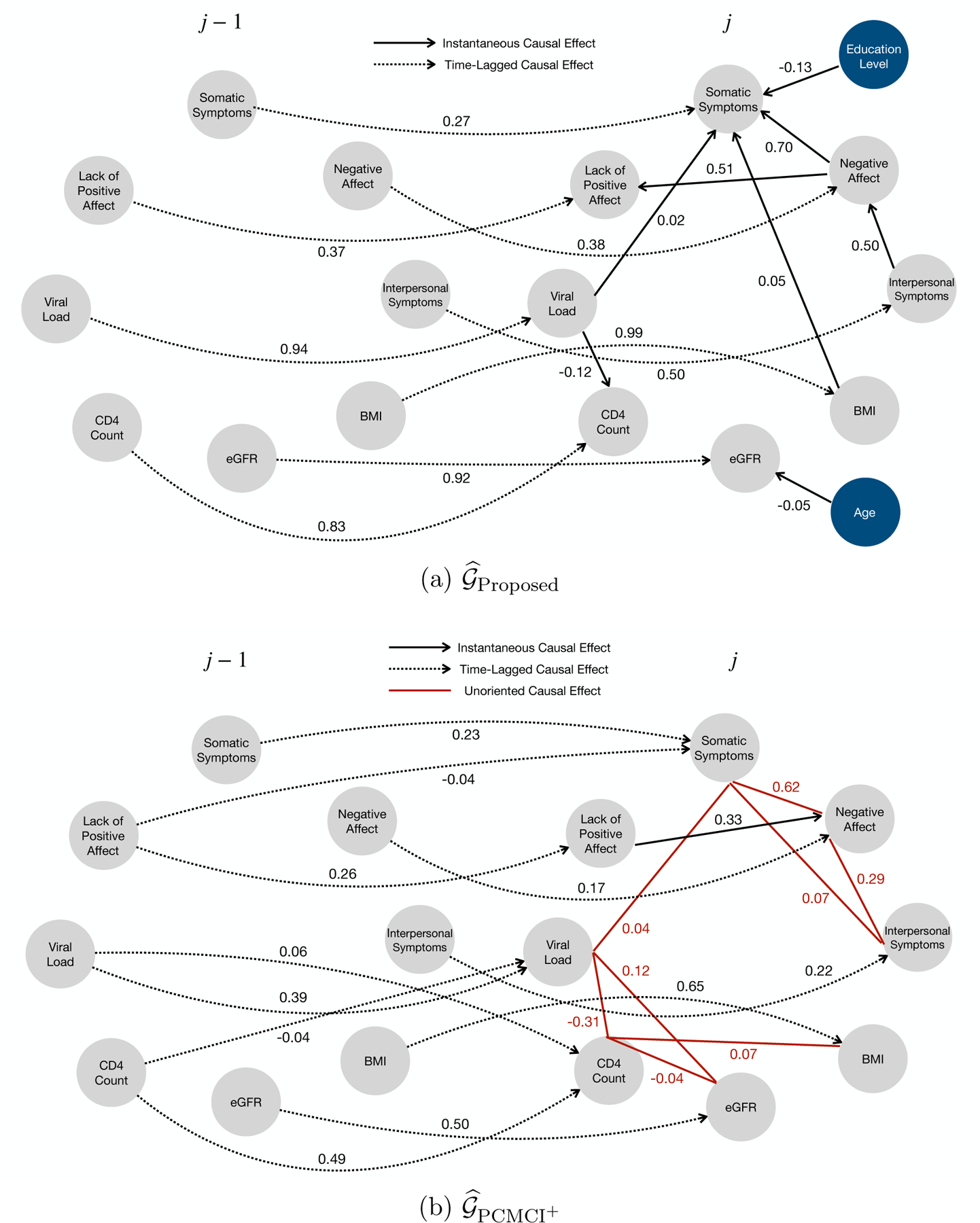
The estimated causal graphs under the proposed model (i.e., ${\hat{𝒢}}_{\text{Proposed}}$) and the alternative method PCMCI^+^ (i.e., ${\hat{𝒢}}_{{\text{PCMCI}}^{+}}$) in the WIHS data analysis. The grey and blue circles represent the longitudinal health outcomes and covariates, respectively. The solid and dashed black lines indicate the instantaneous and time-lagged causal effects, respectively. The solid red lines indicate the unoriented causal effects.

Figure 12(a,b) summarize the estimated causal graphs under the proposed model and the alternative method PCMCI^+^ in the WIHS data analysis. The grey and blue circles represent the longitudinal health outcomes (i.e., $Y_{jq}$) and the covariates (i.e., $X_{js}$), respectively. The solid and dashed black lines indicate the instantaneous (i.e., $Y_{jq}\leftarrow Y_{jp}$ or $Y_{jq}\leftarrow X_{js}$) and time-lagged (i.e., $Y_{jq}\leftarrow Y_{j-1,p}$) causal effects, respectively. The solid red lines indicate the unoriented causal effects detected by PCMCI^+^. To achieve the unique causal identifiability of the proposed model, we utilize the preceding measurement $Y_{j-1,q}$ of each longitudinal health outcome $Y_{jq}$ as an instrumental variable. We will discuss the validity of this approach and highlight the advantages of using preceding measurements as instrumental variables compared to covariates such as age and educational level later.

We first report on the estimation of the instantaneous causal effects among longitudinal health outcomes. As shown in Figure 12(a), most of the instantaneous causal relationships revealed by the proposed model were associated with four depression items (i.e., somatic symptoms, negative affect, lack of positive affect, and interpersonal symptoms), which are important clinical measurements reflecting physical well-being and overall quality of life for people with HIV. For example, negative affect was identified as a direct cause for both somatic symptoms and lack of positive affect. Diener and Emmons (1984) examined both the instantaneous and the time-lagged causal effects between negative affect and lack of positive affect, and found that they were positively correlated in the short time period, but were independent in the long term. Charles and Almeida (2006) reported that people’s prior-day negative affect influenced their current-day somatic symptoms in the positive direction. In addition, non-depression health outcomes such as viral load and BMI were also estimated to be direct causes for somatic symptoms. A high level of viral load and obesity/over-weight are both crucial risk factors for somatic symptoms in people with HIV (Roberts et al., 2000; Jain et al., 2021). Furthermore, instantaneous causal relationships were also detected among non-depression health outcomes. For instance, the proposed model discovered a negative causal effect from viral load to CD4 count, which is well-known in the HIV literature since CD4 cell is the primary target of HIV in human body (Vidya Vijayan et al., 2017).

As shown in Figure 12(b), ${\hat{𝒢}}_{{\text{PCMCI}}^{+}}$ shares similar instantaneous causal graph skeleton among health outcomes with ${\hat{𝒢}}_{\text{Proposed}}$. However, compared to ${\hat{𝒢}}_{\text{Proposed}}$, most of the instantaneous causal relationships detected by PCMCI^+^ were unoriented. For example, the causal relationship between viral load and CD4 count was unoriented in ${\hat{𝒢}}_{{\text{PCMCI}}^{+}}$, which should be from the former to the latter according to known clinical knowledge.

Next, we summarize the estimated instantaneous effects between covariates and health outcomes. As shown in Figure 12(a), we found that a lower educational level was estimated to increase the risk for somatic symptoms, which is consistent with existing knowledge (Sayar et al., 2003). Moreover, with aging, people exhibit progressive decreases in eGFR, which eventually leads to the loss of kidney function (Weinstein and Anderson, 2010).

We then present the estimated time-lagged causal effects among different longitudinal health outcomes. For each health outcome $Y_{jq}$, the only significant time-lagged causal effect was estimated to be from its own preceding measurement (i.e, $Y_{jq}\leftarrow Y_{j-1,q}$ for all q) by the proposed model (shown in Figure 12(a)). This implies that, for each q, $Y_{j-1,q}$ can be used as an instrumental variable for $Y_{jq}$, which guarantees the instantaneous causal identifiability according to Corollary 18. However, determining the suitability of age and education level as instrumental variables for eGFR and somatic symptoms, respectively, poses challenges. Specifically, aging is recognized as a risk factor for kidney function loss but may also impact depressive symptoms in individuals with HIV (Jin et al., 2022). Similarly, education level may be a risk factor for non-somatic depressive symptoms (Lee, 2011). This underscores the robust causal identification capabilities of the proposed model compared to general SCMs.

Furthermore, all of these time-lagged causal relationships in ${\hat{𝒢}}_{\text{Proposed}}$ were also captured by PCMCI^+^ (shown in Figure 12(b)). However, there exist a few additional time-lagged causal relationships between different health outcomes in ${\hat{𝒢}}_{{\text{PCMCI}}^{+}}$. For example, viral load at the last visit (i.e., visit j-1) was estimated to positively influence CD4 count at the current visit (i.e., visit j), which was possibly a spurious causal link due to the following three reasons: (i) it is well-known in the HIV literature that a higher level of viral load decreases the CD4 count in both short-term and long-term (Vidya Vijayan et al., 2017); (ii) viral load was estimated to negatively influence CD4 count in the instantaneous causal graph of ${\hat{𝒢}}_{{\text{PCMCI}}^{+}}$; (iii) the estimated time-lagged causal effect between viral load and CD4 count was negligible (i.e., 0.06), compared to both the instantaneous causal effect between viral load and CD4 count (i.e., −0.31), as well as the time-lagged causal effect between viral load at different visits (i.e., 0.39) in ${\hat{𝒢}}_{{\text{PCMCI}}^{+}}$.

Lastly, we did not detect any cycles in the WIHS data analysis. There are two potential explanations: (i) the underlying true causal relationships among these health outcomes for people with HIV may be best represented by a directed acyclic graph, in which case the proposed method correctly identified the causal structure; or (ii) there may be cycles among these health outcomes, but due to data variability and limited sample size, our method may not be able to detect certain edges with small effect sizes. For example, a hypothetical cyclic causal relationship could exist between viral load and somatic symptoms. However, the effect of viral load on somatic symptoms is very small (i.e., 0.02 as shown in Figure 12(a)). The potential effect of somatic symptoms on viral load may be even smaller, making it undetectable by the proposed method given the limited sample size.

## 7. Conclusion

We developed a novel framework for simultaneously discovering the time-lagged and possibly cyclic instantaneous causalities from longitudinal/time-series observational data. To achieve the unique causal identifiability of the proposed model, we required an instrumental condition for directed graphs with joint cycles, which can be viewed as an extension of the causal identification result of Lacerda et al. (2008) from directed graphs with disjoint cycles to all possible directed graphs. We also proposed a Bayesian structural learning procedure that inferred robust and interpretable causal graphs by selecting a parsimony cause set while adjusting for covariate effects. Through both synthetic and real-world data experiments, we demonstrated the advantages of the proposed model in terms of accurately and robustly recovering the underlying causal mechanisms from longitudinal observational data by comparison with state-of-the-art alternative methods. Importantly, by applying the proposed model to a large-scale longitudinal HIV cohort study, we found interesting and clinically meaningful causal relationships among longitudinal health outcomes for people with HIV.

There are several future extensions. First, the unique causal identifiability of the proposed model relies on the assumption of causal sufficiency, since our causal identification theories are established upon the identifiability results of ICA, where the number of latent sources equals the number of observed variables. To account for unmeasured confounders, one potential approach is to leverage the identifiability results of the overcomplete ICA (Hoyer et al., 2008b; Geiger et al., 2015; Salehkaleybar et al., 2020; Adams et al., 2021), in which the number of latent sources exceeds the number of observed variables. Second, the proposed framework is built upon a linear additive model with non-Gaussian noises. Theoretical analyses for more flexible modeling choices such as the non-linear additive model and the functional model, including the development of new causal identification theories and structural learning methods could be a future direction. Lastly, the causal discovery results in the WIHS data analysis illustrate the potential clinical utility of the proposed framework. In particular, obesity/over-weight was estimated to be a direct cause for depressive symptoms, demonstrating the need of effective weight management for people with HIV (Parra-Rodriguez and O’Halloran, 2023). Therefore, the proposed framework can be used by physicians to develop better combination therapies for commorbidites of HIV, potentially improving the long-term health outcomes and quality of life for people with HIV.

## Figures and Tables

**Figure C1:**
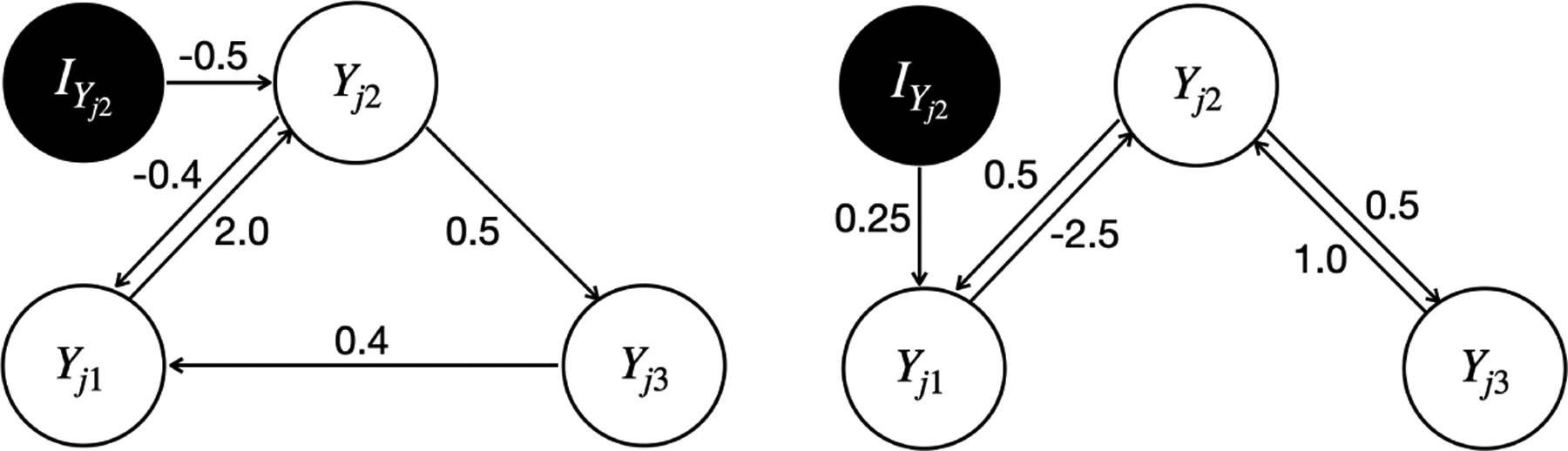
The simulated true causal graph 𝒢 (left) and the only graph ${𝒢}^{\prime }$ (right) associated with a stable SCM in its ICA equivalence class in the additional simulation studies. The instrumental variable $I_{Y_{j2}}$ for $Y_{j2}$ is highlighted by the black circle.

**Figure C2:**
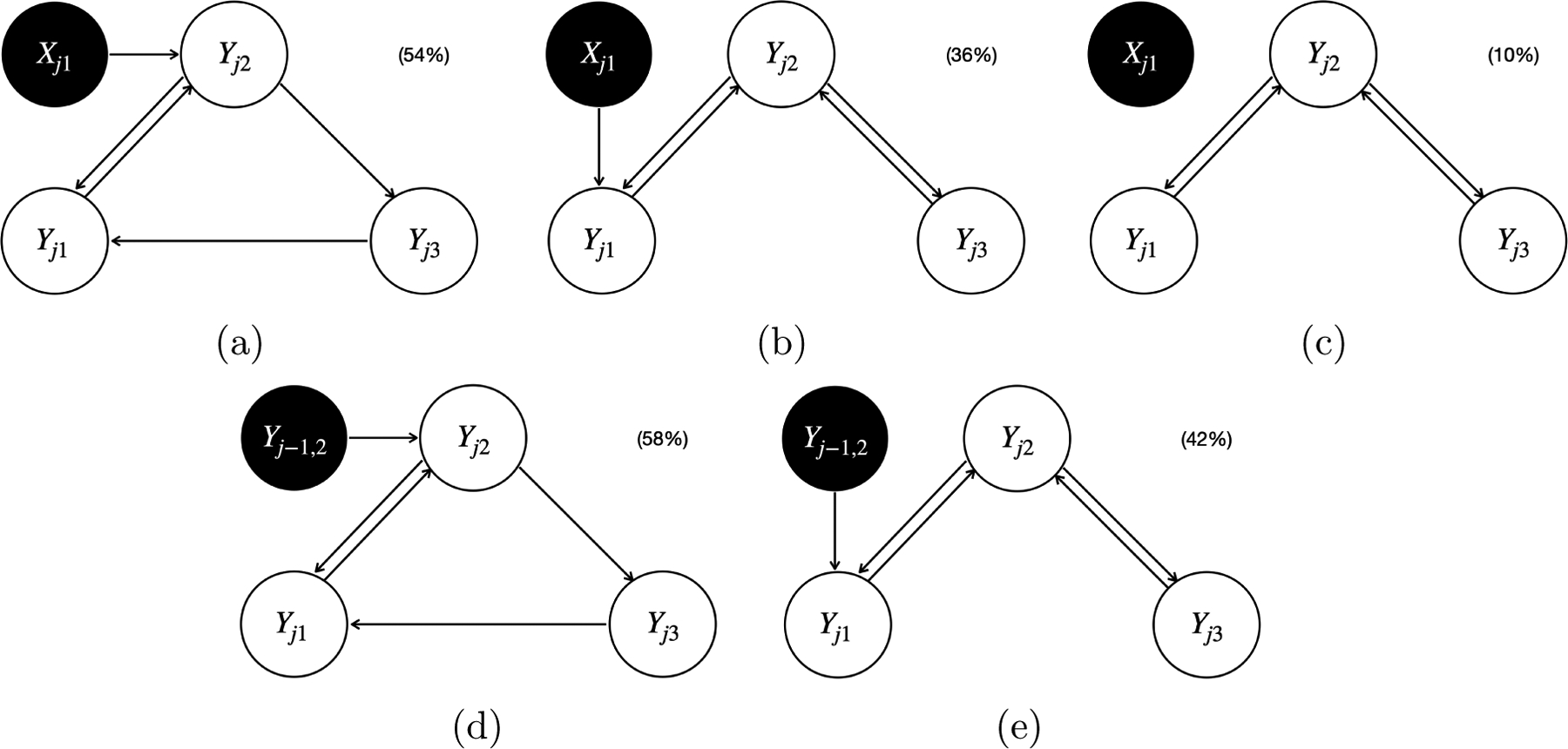
The estimated causal graphs under the proposed model with a sample size of 5,000, using $X_{j1}$ (Panel(a)-(c)) and $Y_{j-1,2}$ (Panel(d)-(e)) as the instrumental variable $I_{Y_{j2}}$ in the additional simulation studies.

**Figure C3:**
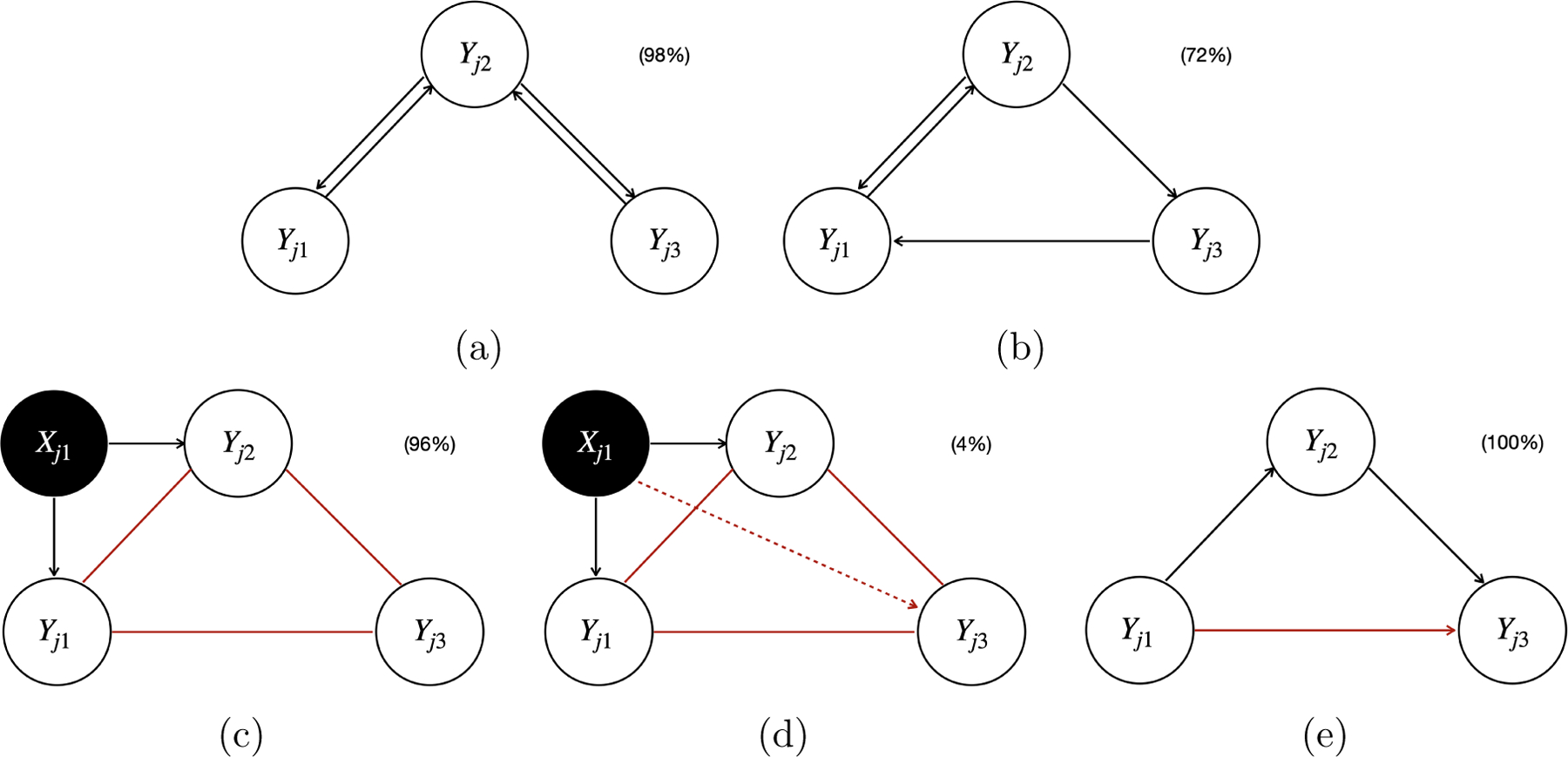
The estimated causal graphs under LiNG-D (Panel(a)-(b)), PCMCI^+^ (Panel(c)-(d)), and VAR-LiNGAM (Panel(e)) with a sample size of 5,000, using $X_{j1}$ as the instrumental variable $I_{Y_{j2}}$ in the additional simulation studies.

**Figure C4:**
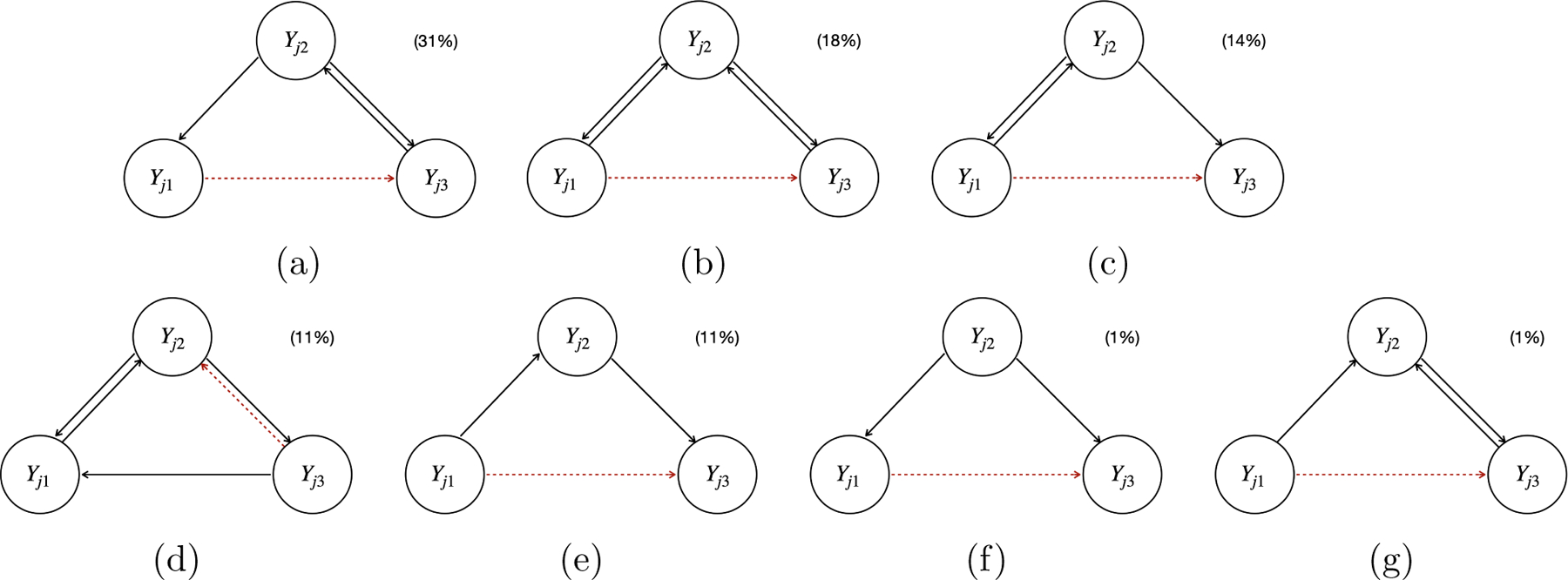
The estimated causal graphs under LiNG-D with a sample size of 5,000, using $Y_{j-1,2}$ as the instrumental variable $I_{Y_{j2}}$ in the additional simulation studies.

**Figure C5:**
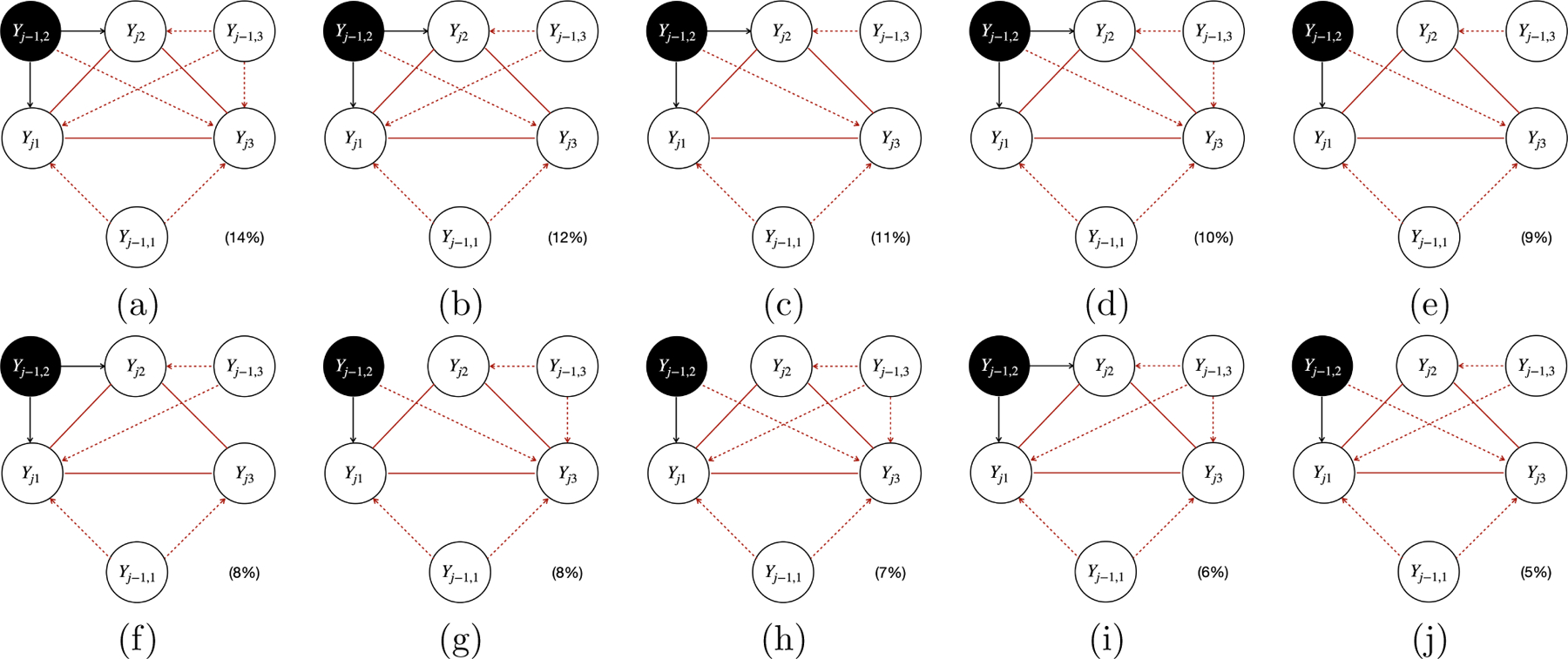
The estimated causal graphs under PCMCI^+^ with a sample size of 5,000, using $Y_{j-1,2}$ as the instrumental variable $I_{Y_{j2}}$ in the additional simulation studies.

**Figure C6:**
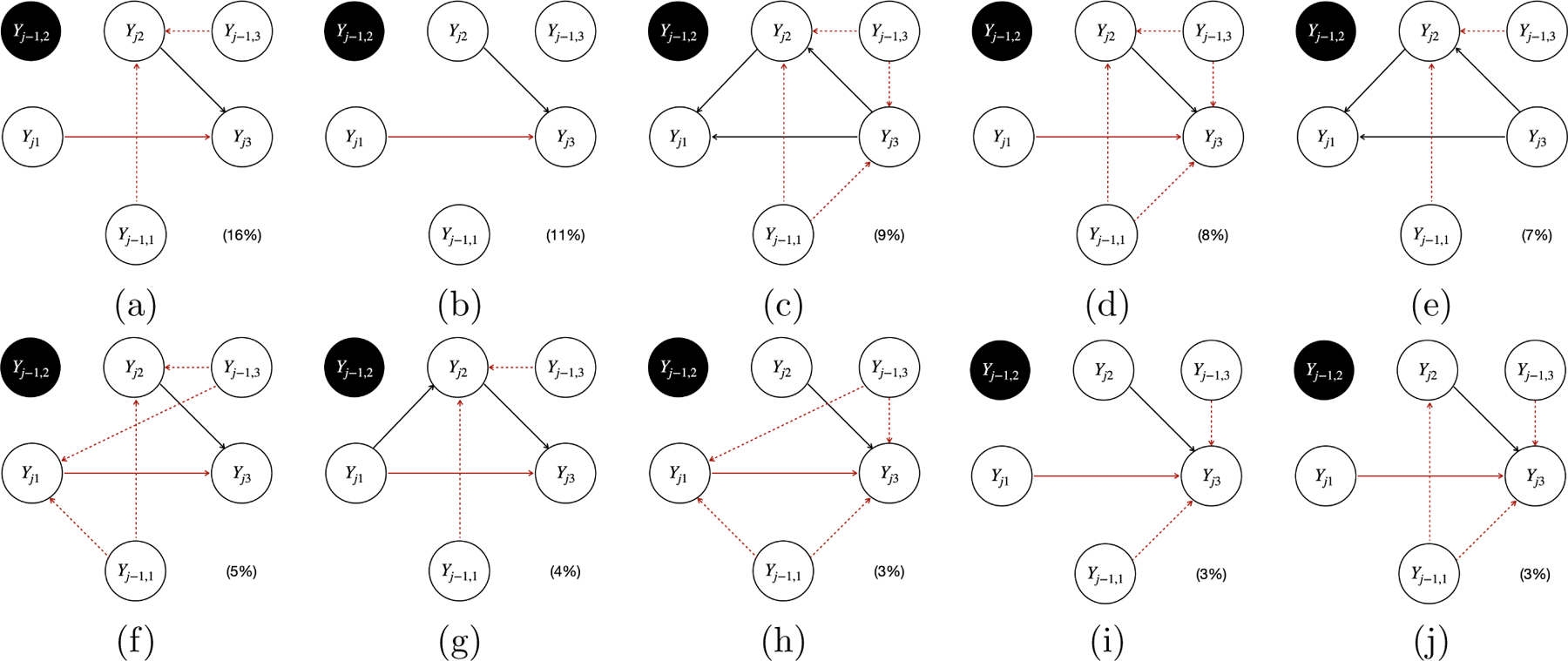
The estimated causal graphs under VAR-LiNGAM with a sample size of 5,000, using $Y_{j-1,2}$ as the instrumental variable $I_{Y_{j2}}$ in the additional simulation studies.

**Figure C7:**
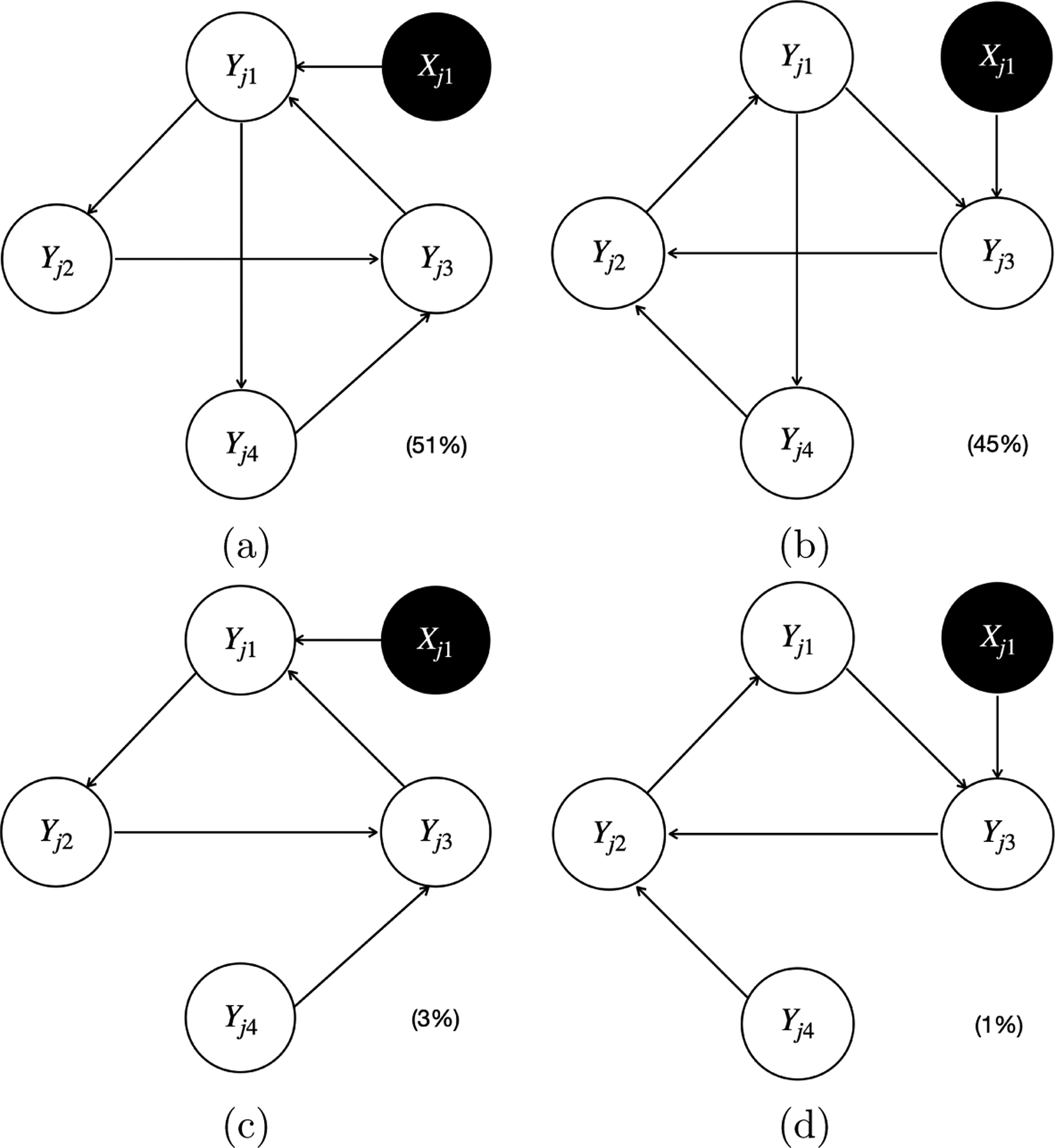
The estimated causal graphs under the proposed model with a varying number of visits $J_{i}$, using $X_{j1}$ as the instrumental variable $I_{Y_{j1}}$.

**Figure C8:**
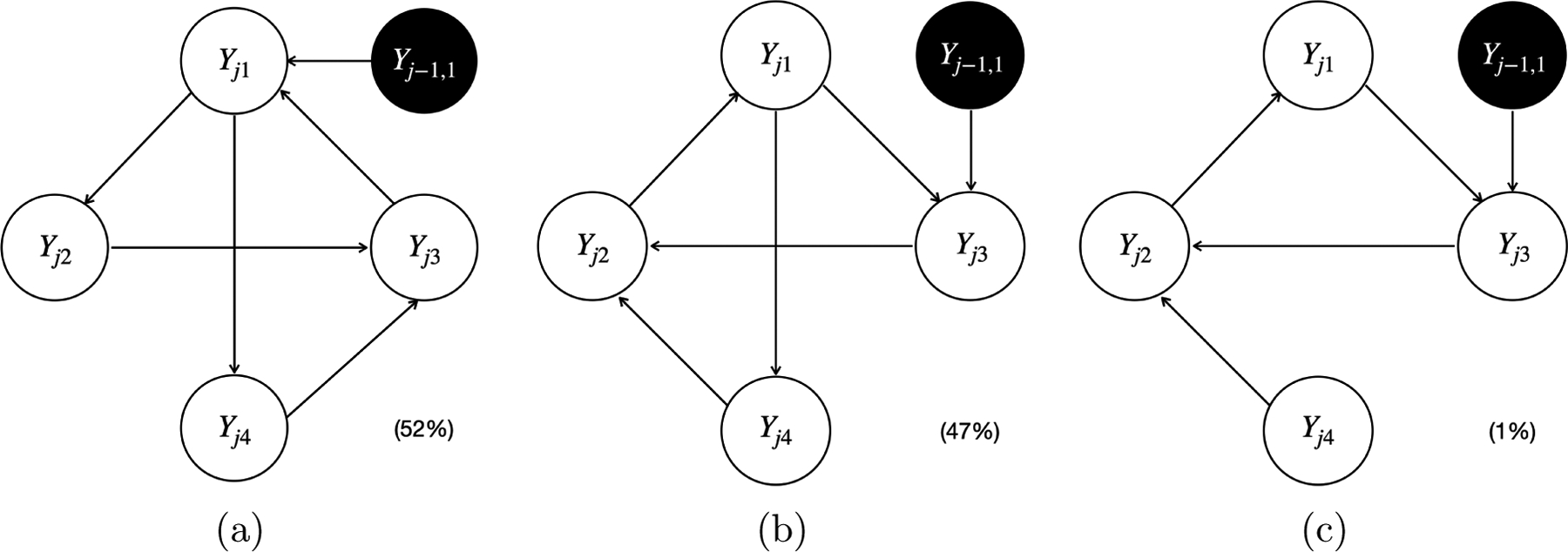
The estimated causal graphs under the proposed model with a varying number of visits $J_{i}$, using $Y_{j-1,1}$ as the instrumental variable $I_{Y_{j1}}$.

## Appendix A. Technical Proofs

### A.1 Proof of Theorem 9

**Proof** Let 𝒢 denote a directed graph with a total number of N directed cycles ${𝒪}_{1},\ldots ,{𝒪}_{N}$, where each pair of the two directed cycles ${𝒪}_{n}$ and ${𝒪}_{n^{\prime }}$ can be joint with each other without complete overlap, i.e., it is possible to have ${𝒪}_{n}\cap {𝒪}_{n^{\prime }}\neq \emptyset$, but not ${𝒪}_{n}\cap {𝒪}_{n^{\prime }}={𝒪}_{n}={𝒪}_{n^{\prime }}$, for $1\leq n\neq n^{\prime }\leq N$. Assume that there exist N variables $Y_{1}\in {𝒪}_{1},\ldots ,Y_{N}\in {𝒪}_{N}$ in 𝒢, each of which has its own instrumental variable $I_{Y_{n}}$, for n=1,…,N. Note that these N variables are not necessarily distinct, i.e., it is possible to have $Y_{n}=Y_{n^{\prime }}$, for $1\leq n\neq n^{\prime }\leq N$.

Let ${𝒢}^{+}$ denote the directed graph formed by incorporating these instrumental variables into 𝒢. By the definition of the instrumental variable (i.e., Definition 2), if the collection of instrumental variables $ℐ=\left\{I_{Y_{1}},\ldots ,I_{Y_{N}}\right\}$ introduces an additional directed cycle ${𝒪}^{+}$ in ${𝒢}^{+}$, then ${𝒪}^{+}$ only involves variables in ℐ, and will be disjoint with ${𝒪}_{1},\ldots ,{𝒪}_{N}$. By Lemma 17, the reversal of disjoint cycles is a necessary condition for two directed graphs to belong to the same ICA equivalence class. Therefore, there exists a one-to-many mapping from the ICA equivalence class of 𝒢 to the ICA equivalence class of ${𝒢}^{+}$. In particular, if ℐ introduces additional directed cycles, each graph in the ICA equivalence class of 𝒢 will correspond to multiple directed graphs in the ICA equivalence class of ${𝒢}^{+}$, and the latter only differ from each other in the part that *only* involves the collection of instrumental variables ℐ. On the other hand, if ℐ does not introduce additional directed cycles, then each graph in the ICA equivalence class of 𝒢 will correspond to only one directed graph in the ICA equivalence class of ${𝒢}^{+}$. We will show later that the unique identification of 𝒢 is independent of the part that *only* involves the collection of instrumental variables ℐ. Therefore, without loss of generality, we assume that ℐ does not introduce additional directed cycles, and then drive the unique identification of 𝒢 from the unique identification of ${𝒢}^{+}$.

Consider any directed cyclic graph ${\left({𝒢}^{+}\right)}^{\prime }$ in the ICA equivalence class of ${𝒢}^{+}$ obtained by performing the two steps described in Lemma 17. Note that by the definition of instrumental variable (i.e., Definition 2), $I_{Y_{n^{⋆}}}$ is a special case of ${\text{pa}}_{{𝒢}^{+}\setminus {\tilde{𝒪}}_{n^{⋆}}}\left(Y_{n^{⋆}}\right)$, for $n^{⋆}=1,\ldots ,N^{⋆}$. In particular, $I_{Y_{n^{⋆}}}\in {𝒢}^{+}\setminus {𝒪}_{n^{⋆}}$ is a parent of $Y_{n^{⋆}}$, and $Y_{n^{⋆}}\in {\tilde{𝒪}}_{n^{⋆}}$ is the only child of $I_{Y_{n^{⋆}}}$ in 𝒢. By Lemma 17(ii), the edge $I_{Y_{n^{⋆}}}\to Y_{n^{⋆}}$ in ${𝒢}^{+}$ will be changed to $I_{Y_{n^{⋆}}}\to {\text{pa}}_{{\tilde{𝒪}}_{n^{⋆}}}\left(Y_{n^{⋆}}\right)$ in ${\left({𝒢}^{+}\right)}^{\prime }$. In other words, the only child of $I_{Y_{n^{⋆}}}$ will be different in 𝒢 and ${𝒢}^{\prime }$ (i.e., $Y_{n^{⋆}}\in 𝒢\neq {\text{pa}}_{{\tilde{𝒪}}_{n^{⋆}}}\left(Y_{n^{⋆}}\right)\in {𝒢}^{\prime }$ due to no self-loops), and thus $I_{Y_{n^{⋆}}}$ will not be an instrumental variable for $Y_{n^{⋆}}$ in ${\left({𝒢}^{+}\right)}^{\prime }$. This is the critical point that leads to the identification of ${𝒢}^{+}$, and it is independent of the part that *only* involves the collection of instrumental variables ℐ in ${𝒢}^{+}$.

In summary, we can identify ${𝒢}^{+}$ from ${\left({𝒢}^{+}\right)}^{\prime }$ by utilizing a collection of instrumental variables $I_{Y_{n^{⋆}}}$ for $Y_{n^{⋆}}\in {\tilde{𝒪}}_{n^{⋆}}$, where $n^{⋆}=1,\ldots ,N^{⋆}$. We finish the proof by noting that the above argument can be applied to any ${\left({𝒢}^{+}\right)}^{\prime }$ within the ICA equivalence class of ${𝒢}^{+}$. ■

### A.2 Proof of Lemma 14

**Proof** Let $\phi :R\to R,R=\left\{r_{1},\ldots ,r_{K}\right\}$, denote an irreducible row-permutation applied to the unmixing matrix W associated with a directed graph 𝒢 is admissible. Let ${𝒢}^{\prime }$ denote the directed graph associated with the resulting unmixing matrix $W^{\prime }$ by applying ϕ to W.

- Note that if $\phi \left(r_{k}\right)=r_{k^{\prime }}$, then we have $W_{r_{k},r_{k^{\prime }}}\neq 0$, otherwise ϕ is not admissible. In other words, there exists a directed edge $\left(Y_{r_{k^{\prime }}}\to Y_{r_{k}}\right)\in ℰ$ in the directed graph 𝒢.
  Without loss of generality, we assume that $\phi \left(r_{1}\right)=r_{2}$, which implies $\left(Y_{r_{2}}\to Y_{r_{1}}\right)\in ℰ$. Then we need to determine $\phi \left(r_{2}\right)=r_{k^{\prime }}$ for some $k^{\prime }\neq 2$. In addition, we have $k^{\prime }\neq 1$ due to the irreducible assumption. Similarly, we assume that $\phi \left(r_{2}\right)=r_{3}$, which implies $\left(Y_{r_{3}}\to Y_{r_{2}}\right)\in ℰ$. Same argument can be applied to k=3,…,K-1, i.e., $\phi \left(r_{k}\right)=r_{k+1}$, which indicates the existence of directed edges $\left(Y_{r_{3}}\to Y_{r_{2}}\right)\in ℰ,\ldots ,\left(Y_{r_{K}}\to Y_{r_{K-1}}\right)\in ℰ$. Now we need to determine $\phi \left(r_{K}\right)=r_{k^{\prime }}$ for some $k^{\prime }\neq K$. Due to the irreducible assumption, we have $k^{\prime }\neq 2,\ldots ,K-1$. Therefore, $k^{\prime }=1$, which implies $\left(Y_{r_{1}}\to Y_{r_{K}}\right)\in ℰ$, and also the existence of the directed cycle $𝒪:Y_{r_{1}}\leftarrow Y_{r_{2}}\leftarrow \cdots \leftarrow Y_{r_{K}}\leftarrow Y_{r_{1}}$ in 𝒢.
  In addition, ϕ reverses the direction of 𝒪, i.e., we have ${𝒪}^{\prime }:Y_{r_{1}}\to Y_{r_{2}}\to \cdots \to Y_{r_{K}}\to Y_{r_{1}}$ in the directed graph ${𝒢}^{\prime }$ associated with the resulting unmixing matrix $W^{\prime }$.
- Without loss of generality, assume $\phi \left(r_{k}\right)=r_{k+1}$, for k=1,…,K-1, and $\phi \left(r_{K}\right)=r_{1}$, which indicates the existence of the directed cycle $𝒪:Y_{r_{1}}\leftarrow Y_{r_{2}}\leftarrow \cdots \leftarrow Y_{r_{K}}\leftarrow Y_{r_{1}}$ in 𝒢, by the above argument. Here we provide proof for the cases where k ranges from 1 to K-1, but note that the case k=K can be proven in a similar manner.
  Since $\phi \left(r_{k}\right)=r_{k+1}$, for k=1,…,K-1, then the $r_{k+1}$-th row of $W^{\prime }$ is equivalent to the $r_{k}$-th row of W after appropriate row-scaling. This implies that ${\text{pa}}_{𝒢\setminus 𝒪}\left(Y_{r_{k}}\right)$ will be the parent of $Y_{r_{k+1}}$ in ${𝒢}^{\prime }$, for k=1,…,K-1. The proof is finished by noting that $Y_{r_{k+1}}$ is the only parent of $Y_{r_{k}}$ inside the directed cycle 𝒪 in 𝒢, i.e., $Y_{r_{k+1}}={\text{pa}}_{𝒪}\left(Y_{r_{k}}\right)$. ■

### A.3 Proof of Proposition 16

**Proof** The proposition is trivial if the admissible row-permutation ϕ is irreducible.

Now assume that ϕ is not irreducible. By the definition of irreducible row-permutation, there exists a row-permutation $\phi_{1}:R_{1}\to R_{1}$, where $R_{1}\subset R$, such that $\phi_{1}\left(R_{1}\right)=\phi \left(R_{1}\right)=R_{1}$. Let $R_{2}=R\setminus R_{1}$, then there exists another row-permutation $\phi_{2}:R_{2}\to R_{2}$ such that $\phi_{2}\left(R_{2}\right)=\phi \left(R_{2}\right)=R_{2}$. This is because if $\phi_{2}$ is not a bijective mapping from $R_{2}$ to $R_{2}$, then ϕ can not be a bijective mapping from R to R. Furthermore, since $R_{1}\cap R_{2}=\emptyset$, both $\phi_{1}$ and $\phi_{2}$ are admissible, otherwise ϕ can not be an admissible row-permutation. Therefore, $\left\{\phi_{1},\phi_{2}\right\}$ forms a collection of disjoint admissible row-permutations that is equivalent to ϕ.

If both $\phi_{1}$ and $\phi_{2}$ are irreducible, then the proposition is proved. Since |R|<∞, if $\phi_{1}$ and $\phi_{2}$ are not irreducible, then we can always apply the aforementioned procedure finite times to find two collections of disjoint admissible row-permutations that are equivalent to $\phi_{1}$ and $\phi_{2}$, respectively, each of which only consists of row-permutations that are irreducible. Combining these two collections, we have a collection of disjoint admissible irreducible row-permutations that is equivalent to ϕ. ■

### A.4 Proof of Lemma 17

**Proof** The first part of this Lemma is a direct corollary of Lemma 14 and Proposition 16.

Now consider applying steps (i) and (ii) to any choice of disjoint cycles in any directed graph 𝒢. Without loss of generality, assume that the disjoint directed cycles are ${\tilde{𝒪}}_{n^{⋆}}:Y_{r_{1}}\leftarrow Y_{r_{2}}\leftarrow \cdots \leftarrow Y_{r_{K_{n^{⋆}}}}\leftarrow Y_{r_{1}}$, for $n^{⋆}=1,\ldots ,N^{⋆}$. Then performing steps (i) and (ii) on 𝒢 is equivalent to applying the row-permutation ϕ to the unmixing matrix W associated with 𝒢, where $\phi \left(r_{k_{n^{⋆}}}\right)=r_{k_{n^{⋆}}+1}$ for $k_{n^{⋆}}=1,\ldots ,K_{n^{⋆}}-1$ and $\phi \left(r_{K_{n^{⋆}}}\right)=\phi \left(r_{1}\right),n^{⋆}=1,\ldots ,N^{⋆}$. Note that due to the existence of edges $Y_{r_{k_{n^{⋆}}}}\leftarrow Y_{r_{k_{n^{⋆}}+1}}$ for $k_{n^{⋆}}=1,\ldots ,K_{n^{⋆}}-1$ and $Y_{r_{K_{n^{⋆}}}}\leftarrow Y_{r_{1}}$, the row-permutation ϕ is admissible. Consequently, the resulting directed graph ${𝒢}^{\prime \prime }$ remains in the same ICA equivalence class as 𝒢. ■

### A.5 Proof of Corollary 18

**Proof** Consider the following coefficient matrix B of the proposed model (1):

$$B=\left(\begin{array}{cccccccccccccc} Y_{J} & Y_{J-1} & \cdots & Y_{J-L_{y}} & Y_{J-L_{y}-1} & \cdots & Y_{1} & X_{J} & X_{J-1} & \cdots & X_{J-L_{x}} & X_{J-L_{x}-1} & \cdots & X_{1}\\ B_{0} & B_{1} & \cdots & B_{L_{y}} & 0 & \cdots & 0 & A_{0} & A_{1} & \cdots & A_{L_{x}} & 0 & \cdots & 0\\ 0 & B_{0} & \cdots & B_{L_{y}-1} & B_{L_{y}} & \cdots & 0 & 0 & A_{0} & \cdots & A_{L_{x}-1} & A_{L_{x}} & \cdots & 0\\ \vdots & \vdots & \vdots & \vdots & \vdots & \vdots & \vdots & \vdots & \vdots & \vdots & \vdots & \vdots & \vdots & \vdots \\ 0 & 0 & \cdots & B_{0} & B_{1} & \cdots & 0 & 0 & 0 & \cdots & A_{0} & A_{1} & \cdots & 0\\ 0 & 0 & \cdots & 0 & B_{0} & \cdots & 0 & 0 & 0 & \cdots & 0 & A_{0} & \cdots & 0\\ \vdots & \vdots & \vdots & \vdots & \vdots & \vdots & \vdots & \vdots & \vdots & \vdots & \vdots & \vdots & 0 & \vdots \\ 0 & 0 & \cdots & 0 & 0 & 0 & B_{0} & 0 & 0 & \cdots & 0 & 0 & 0 & A_{0} \end{array}\right)\begin{array}{c} Y_{J}\\ Y_{J-1}\\ \vdots \\ Y_{J-L_{y}}\\ Y_{J-L_{y}-1}\\ \vdots \\ Y_{1} \end{array},$$

and the following simpler (cross-sectional) SCM defined by the instantaneous causal effects $B_{0}$ at each time point j,

$$Y_{j}=B_{0}Y_{j}+E_{j},$$

where $Y_{j}=\left(Y_{j1},\ldots ,Y_{jQ}\right)$ and $X_{j}=\left(X_{j1},\ldots ,X_{jS}\right)$, j=1,2,…,J. We will first prove that the unique identification of the proposed model (1) (with the graph 𝒢) can be achieved by leveraging the unique identification of the SCM (with the graph ${𝒢}_{j}$) described above.

By Lemma 17, any two directed graphs within the same ICA equivalence class can be obtained from each other by performing two steps, both of which are uniquely determined by the disjoint cycles to be reversed. Consequently, for any two directed graphs that share the same cycles, a one-to-one correspondence exists between their ICA equivalence classes. Note that the *only* potential cycles in B are within the instantaneous causal effects $B_{0}$, which characterizes the same causal relationships for $Y_{j}$’s at any time point j. In other words, if ${𝒢}^{\prime }$ is obtained by reversing disjoint cycles in $B_{0}$ of 𝒢, this reversal of disjoint cycles will be applied *simultaneously* to all the rows associated with $Y_{1},\ldots ,Y_{J}$ in B. Therefore, 𝒢 and ${𝒢}_{j}$ essentially share the same cycles, and there exists a one-to-one correspondence between their ICA equivalence classes. Then the unique identification of 𝒢 can be achieved through the unique identification of ${𝒢}_{j}$.

The proof is concluded by demonstrating the unique identification of ${𝒢}_{j}$. By Theorem 9, for any cycle ${𝒪}_{j}$ in ${𝒢}_{j}$, if there exists a variable $Y_{jq}\in {𝒪}_{j}$ which has an instrumental variable $I_{Y_{jq}}$, then ${𝒢}_{j}$ can be uniquely identified from its ICA equivalence class. ■

## Appendix B. MCMC Algorithm

### 1. Update $\beta_{ℒqp}$ and $\alpha_{ℒqs}$

The full conditional distribution for $\beta_{\ell qp}$ and $\alpha_{\ell qs}$ is

$$p\left(\beta_{\ell qp},\alpha_{\ell qs}|\cdot \right)\propto p\left(\beta_{\ell qp}\right)p\left(\alpha_{\ell qs}\right)\prod_{i}\prod_{j=\ell +1}^{J_{i}}𝒩\left(Y_{ijq}|Y_{ijq}^{*},\frac{\sigma_{q}^{2}}{\tau_{ijq}}\right)\left|I-B_{0}\right|,$$

where

$$Y_{ijq}^{*}=\mu_{q}+\sum_{\ell^{\prime }=0}^{L_{y}^{\prime }}\sum_{p=1}^{Q}Y_{i,j-\ell^{\prime },p}\beta_{\ell^{\prime }qp}+\sum_{\ell^{\prime }=0}^{L_{x}^{\prime }}\sum_{s=1}^{S}X_{i,j-\ell^{\prime },s}\alpha_{\ell^{\prime }qs},$$

$$L_{y}^{\prime }=\text{min}\left(j-1,L_{y}\right),\;\text{and}\;L_{x}^{\prime }=\text{min}\left(j-1,L_{x}\right).$$

#### 1.1. Update $\beta_{0qp}$

Since the full conditional distribution for $\beta_{0qp}$ involves the additional term $|I-B_{0}|$, there is no closed form solution. Therefore, we will update it using the Metropolis-Hasting algorithm. At each Metropolis-Hasting step, we will accept the move only if the maximum modulus of $B_{0}$’s eigenvalues is strictly less than 1, to ensure that the proposed model is well-defined.

#### 1.2. Update $\beta_{\ell qp},\ell >0$ and $\alpha_{\ell qs},\ell \geq 0$

The full conditional distribution for $\beta_{\ell qp},\ell >0$ is

$$p\left(\beta_{\ell qp}|\cdot \right)\propto 𝒩\left(0,\gamma_{\ell qp}^{\beta }\nu_{\ell qp}^{\beta }\right)\prod_{i}\prod_{j=\ell +1}^{J_{i}}𝒩\left(Y_{ijq}|Y_{ijq}^{*},\frac{\sigma_{q}^{2}}{\tau_{ijq}}\right)\propto 𝒩\left(\mu_{\beta },\sigma_{\beta }^{2}\right),$$

where

$$\sigma_{\beta }^{2}={\left(\sum_{i}\sum_{j=\ell +1}^{J_{i}}\frac{{\left(Y_{i,j-\ell ,p}\right)}^{2}}{\sigma_{q}^{2}/\tau_{ijq}}+\frac{1}{\gamma_{\ell qp}^{\beta }\nu_{\ell qp}^{\beta }}\right)}^{-1},$$

$$\mu_{\beta }=\sigma_{\beta }^{2}\left(\sum_{i}\sum_{j=\ell +1}^{J_{i}}\frac{Y_{i,j-\ell ,p}}{\sigma_{q}^{2}/\tau_{ijq}}{\tilde{Y}}_{ijq}\right),$$

$${\tilde{Y}}_{ijq}=Y_{ijq}-\mu_{q}-\sum_{\ell^{\prime },p^{\prime }\neq \ell ,p}Y_{i,j-\ell^{\prime },p^{\prime }}\beta_{\ell^{\prime }qp^{\prime }}-\sum_{\ell^{\prime }=0}^{L_{x}^{\prime }}\sum_{s=1}^{S}X_{i,j-\ell^{\prime },s}\alpha_{\ell^{\prime }qs}.$$

We can update $\alpha_{\ell qs},\ell \geq 0$ analogously.

### 2. Update $\gamma_{ℒqp}^{\beta }$ and $\gamma_{ℒqs}^{\alpha }$

The full conditional distribution for $\gamma_{\ell qp}^{\beta }$ is

$$p\left(\gamma_{\ell qp}^{\beta }|\cdot \right)\propto \left\{\rho^{\beta }\delta_{1}\left(\gamma_{\ell qp}^{\beta }\right)+\left(1-\rho^{\beta }\right)\delta_{\nu_{0}}\left(\gamma_{\ell qp}^{\beta }\right)\right\}𝒩\left(\beta_{\ell qp}|0,\gamma_{\ell qp}^{\beta }\nu_{\ell qp}^{\beta }\right),$$

i.e.,

$$\frac{P\left(\gamma_{\ell qp}^{\beta }=1|\beta_{\ell qp},\nu_{\ell qp}^{\beta },\rho^{\beta }\right)}{P\left(\gamma_{\ell qp}^{\beta }=\nu_{0}|\beta_{\ell qp},\nu_{\ell qp}^{\beta },\rho^{\beta }\right)}=\frac{\sqrt{\nu_{0}}\rho^{\beta }}{1-\rho^{\beta }}\text{exp}\left\{\frac{\left(1-\nu_{0}\right){\left(\beta_{\ell qp}\right)}^{2}}{2\nu_{0}\nu_{\ell qp}^{\beta }}\right\}.$$

We can update $\gamma_{\ell qS}^{\alpha }$ analogously.

### 3. Update $\nu_{ℒqp}^{\beta }$ and $\nu_{ℒqs}^{\alpha }$

The full conditional distribution for $\nu_{\ell qp}^{\beta }$ is

$$p\left(\nu_{\ell qp}^{\beta }|\cdot \right)\propto \text{Inverse-Gamma}\left(a_{\nu },b_{\nu }\right)𝒩\left(\beta_{\ell qp}|0,\gamma_{\ell qp}^{\beta }\nu_{\ell qp}^{\beta }\right)\;\propto \text{Inverse-Gamma}\left(a_{\nu }+\frac{1}{2},b_{\nu }+\frac{{\left(\beta_{\ell qp}\right)}^{2}}{2\gamma_{\ell qp}^{\beta }}\right).$$

We can update $\nu_{\ell qs}^{\alpha }$ analogously.

### 4. Update $\rho^{\beta }$ and $\rho^{\alpha }$

The full conditional distribution for $\rho^{\beta }$ is

$$p\left(\rho^{\beta }|\cdot \right)\propto \text{Beta}\left(a_{\rho },b_{\rho }\right)\prod_{\ell =0}^{L_{y}}\prod_{q=1}^{Q}\prod_{p=1}^{Q}\left\{\rho^{\beta }\delta_{1}\left(\gamma_{\ell qp}^{\beta }\right)+\left(1-\rho^{\beta }\right)\delta_{\nu_{0}}\left(\gamma_{\ell qp}^{\beta }\right)\right\}\;\propto \text{Beta}\left(a_{\rho }+\sum_{\ell =0}^{L_{y}}\sum_{q=1}^{Q}\sum_{p=1}^{Q}\delta_{1}\left(\gamma_{\ell qp}^{\beta }\right),b_{\rho }+\sum_{\ell =0}^{L_{y}}\sum_{q=1}^{Q}\sum_{p=1}^{Q}\delta_{\nu_{0}}\left(\gamma_{\ell qp}^{\beta }\right)\right).$$

We can update $\rho^{\alpha }$ analogously.

### 5. Update $\tau_{ijq}$

The full conditional distribution for $\tau_{ijq}$ is

$$p\left(\tau_{ijq}|\cdot \right)\propto \text{Inverse-Gamma}\left(1,\frac{1}{8}\right)𝒩\left(Y_{ijq}|Y_{ijq}^{*},\frac{\sigma_{q}^{2}}{\tau_{ijq}}\right)\;\propto \text{Inverse-Gaussian}\left(\frac{\sigma_{q}}{2\left|Y_{ijq}-Y_{ijq}^{*}\right|},\frac{1}{4}\right).$$

### 6. Update $\mu_{q}$

The full conditional distribution for $\mu_{q}$ is

$$p\left(\mu_{q}|\cdot \right)\propto 𝒩\left(0,\sigma_{\mu }^{2}\right)\prod_{i}\prod_{j=1}^{J_{i}}𝒩\left(Y_{ijq}|Y_{ijq}^{*},\frac{\sigma_{q}^{2}}{\tau_{ijq}}\right)\propto 𝒩\left(\mu_{n},\sigma_{n}^{2}\right),$$

where

$$\sigma_{n}^{2}={\left(\sum_{i}\sum_{j=1}^{J_{i}}\frac{\tau_{ijq}}{\sigma_{q}^{2}}+\frac{1}{\sigma_{\mu }^{2}}\right)}^{-1},$$

$$\mu_{n}=\sigma_{n}^{2}\left(\sum_{i}\sum_{j=1}^{J_{i}}{\tilde{Y}}_{ijq}\frac{\tau_{ijq}}{\sigma_{n}^{2}}\right),$$

$${\tilde{Y}}_{ijq}=Y_{ijq}-\sum_{\ell^{\prime }=0}^{L_{y}^{\prime }}\sum_{p=1}^{Q}Y_{i,j-\ell^{\prime },p}\beta_{\ell^{\prime }qp}-\sum_{\ell^{\prime }=0}^{L_{x}^{\prime }}\sum_{s=1}^{S}X_{i,j-\ell^{\prime },s}\alpha_{\ell^{\prime }qs}.$$

### 7. Update $\sigma_{q}^{2}$

The full conditional distribution for $\sigma_{q}^{2}$ is

$$p\left(\sigma_{q}^{2}|\cdot \right)\propto \text{Inverse-Gamma}\left(a_{\sigma },b_{\sigma }\right)\prod_{i}\prod_{j=1}^{J_{i}}𝒩\left(Y_{ijq}|Y_{ijq}^{*},\frac{\sigma_{q}^{2}}{\tau_{ijq}}\right)\;\propto \text{Inverse-Gamma}\left(a_{\sigma }+\frac{1}{2}\sum_{i}\sum_{j=1}^{J_{i}}1,b_{\sigma }+\frac{1}{2}\sum_{i}\sum_{j=1}^{J_{i}}{\left(Y_{ijq}-Y_{ijq}^{*}\right)}^{2}\tau_{ijq}\right).$$

## Appendix C. Additional Simulation Studies

### C.1 Identifying ICA Equivalence Classes

To further illustrate the superior performance of the proposed Bayesian learning algorithm in identifying ICA equivalence classes, we conducted additional simulation studies using a different simulated true causal graph from the one discussed in Section 5.1. In particular, the simulated true causal graph was set to be 𝒢 in the left panel of Figure C1 with an instrumental variable $I_{Y_{j2}}$ for $Y_{j2}$. The right panel of Figure C1 plots the only graph ${𝒢}^{\prime }$ in the ICA equivalence class of 𝒢 for which the corresponding SCM is stable. We considered two types of instrumental variables: $I_{Y_{j2}}=X_{j1}\sim N(0,1)$, and $I_{Y_{j2}}=Y_{j-1,2}$. We generated the simulated true $Y_{ijq}$ using the following data-generating process,

$$\left\{\begin{array}{l} Y_{ij1}=\mu_{1}-0.4\times Y_{ij2}+0.4\times Y_{ij3}+e_{ij1},\\ Y_{ij2}=\mu_{2}+2\times Y_{ij1}-0.5\times I_{Y_{ij2}}+e_{ij2},\\ Y_{ij3}=\mu_{3}+0.5\times Y_{ij2}+e_{ij3}. \end{array}\right.$$

The rest of the simulation setup was the same as in Section 5.1.

Figures C2 summarizes all the individual casual graphs identified by the proposed model when $X_{j1}$ and $Y_{j-1,2}$ were used as the instrumental variable $I_{Y_{j2}}$. The proposed Bayesian learning algorithm successfully identified 𝒢 and ${𝒢}^{\prime }$ in the ICA equivalence class.

Figures C3–C6 present the estimated causal graphs under alternative methods. When $Y_{j-1,2}$ was used as the instrumental variable $I_{Y_{j2}}$, PCMCI^+^ and VAR-LiNGAM identified 14 and 32 individual graphs across 100 replications, respectively. Therefore, for clarity and effective presentation, we reported only the top 10 individual causal graphs identified by PCMCI^+^ and VAR-LiNGAM based on their relative frequency across 100 replications.

As shown in Figures C3–C6, PCMCI^+^ and VAR-LiNGAM failed to identify the instantaneous directed cycles. While LiNG-D successfully recovered the ICA equivalence classes when $X_{j1}$ was used as the instrumental variable $I_{Y_{j2}}$, it produced spurious causal links when the time-lagged variable $Y_{j-1,2}$ was used as the instrumental variable $I_{Y_{j2}}$. Similarly, both PCMCI^+^ and VAR-LiNGAM also detected spurious causal links under these scenarios.

In summary, the proposed Bayesian learning algorithm demonstrated the best performance in identifying ICA equivalence classes compared to all alternative methods.

### C.2 Varying Number of Visits $J_{i}$

To empirically verify the causal identification theory under the general case where different individuals have varying numbers of visits $J_{i}$, we conducted additional simulation studies. Specifically, we assumed that there were 1,000 individuals and the number of visits $J_{i}$ was randomly generated between four and eight, instead of fixing at five. The other simulation setups remained the same as in simulation scenario I.

Figures C7 and C8 present all the individual causal graphs identified by the proposed model across 100 repeated experiments, using $X_{j1}$ and $Y_{j-1,1}$ as the instrumental variable $I_{Y_{j1}}$, respectively. We observed similar results to those in simulation scenario I, indicating that our causal identification theory applies effectively to general cases with varying $J_{i}$.

## Appendix D. Prior Specification for WIHS Data Analysis

For better biomedical interpretations in the WIHS data analysis, we slightly modified the spike-and-slab priors on both $\beta_{\ell qp}$ and $\alpha_{\ell qs}$ introduced in Section 4 of the manuscript. We describe the prior for $\beta_{\ell qp}$, and the prior for $\alpha_{\ell qs}$ is analogously defined.

Specifically, we impose a conditional prior on the instantaneous (i.e., $\beta_{0qp}$) and time-lagged effects $\beta_{\ell qp},\ell >0$ by assuming that (i) $\beta_{\ell qp}=0,\ell >0$ if $\beta_{0qp}=0$; and (ii) sign($\beta_{\ell qp}$) is the same for all ℓ≥0 such that $\beta_{\ell qp}\neq 0$. We expand $\beta_{\ell qp}=\eta_{\ell qp}\xi_{qp}$ to be a product of two scalars $\eta_{\ell qp}$ and $\xi_{qp}$. The scalar $\xi_{qp}$ is a sign indicator for $\beta_{\ell qp}$, which represents whether the causal effect is positive or negative when $\beta_{\ell qp}\neq 0$. We assume that if there exist causal relationships between two variables, then the instantaneous (i.e., $Y_{jq}\leftarrow Y_{jp},p\neq q$) and time-lagged (i.e., $Y_{jq}\leftarrow Y_{j-\ell ,p},\ell >0$) effects are on the same direction, therefore, $\xi_{qp}$ does not depend on ℓ. We assign equal prior probabilities for either direction, i.e., $P\left(\xi_{qp}=1\right)=P\left(\xi_{qp}=-1\right)=0.5$. The (non-negative) scalar $\eta_{\ell qp}$ is assigned a spike-and-slab prior $\eta_{\ell qp}\sim {𝒩}^{+}\left(0,\gamma_{\ell qp}\nu_{\ell qp}\right)$ with $\nu_{\ell qp}\sim \text{Inverse-Gamma}\left(a_{\nu },b_{\nu }\right)$ and $\gamma_{\ell qp}\sim \rho \delta_{1}\left(\gamma_{\ell qp}\right)+(1-\rho )\delta_{\nu_{0}}\left(\gamma_{\ell qp}\right)$, where ${𝒩}^{+}$ denotes the normal distribution truncated by [0,∞), and $\delta_{x}(\cdot )$ denotes the Dirac measure at x. By the conditional prior assumption, $\gamma_{\ell qp}=\nu_{0},\ell >0$ if $\gamma_{0qp}=\nu_{0}$.

The rest of the prior specifications are the same as before. For the MCMC algorithm, we only need to replace Step 1 in Appendix B with the following Step 1*.

### 1*. Update $\beta_{ℒqp}=\eta_{ℒqp}^{\beta }\xi_{qp}^{\beta }$ and $\alpha_{ℒqs}=\eta_{ℒqs}^{\alpha }\xi_{qs}^{\alpha }$

The full conditional distribution for $\beta_{\ell qp}$ and $\alpha_{\ell qs}$ is

$$p\left(\beta_{\ell qp},\alpha_{\ell qs}|\cdot \right)\propto p\left(\beta_{\ell qp}\right)p\left(\alpha_{\ell qs}\right)\prod_{i}\prod_{j=\ell +1}^{J_{i}}𝒩\left(Y_{ijq}|Y_{ijq}^{*},\frac{\sigma_{q}^{2}}{\tau_{ijq}}\right)\left|I-B_{0}\right|,$$

where

$$Y_{ijq}^{*}=\mu_{q}+\sum_{\ell^{\prime }=0}^{L_{y}^{\prime }}\sum_{p=1}^{Q}Y_{i,j-\ell^{\prime },p}\beta_{\ell^{\prime }qp}+\sum_{\ell^{\prime }=0}^{L_{x}^{\prime }}\sum_{s=1}^{S}X_{i,j-\ell^{\prime },s}\alpha_{\ell^{\prime }qs},$$

$$L_{y}^{\prime }=\text{min}\left(j-1,L_{y}\right),\;\text{and}\;L_{x}^{\prime }=\text{min}\left(j-1,L_{x}\right).$$

#### 1.1*. Update $\xi_{qp}^{\beta }$ and $\xi_{qs}^{\alpha }$

The full conditional distribution for $\xi_{qp}^{\beta }$ is

$$\frac{P\left(\xi_{qp}^{\beta }=1|\cdot \right)}{P\left(\xi_{qp}^{\beta }=-1|\cdot \right)}\propto \text{exp}\left(\sum_{i}\sum_{j=1}^{J_{i}}\left\{2\frac{{\tilde{Y}}_{ijq}\sum_{\ell^{\prime }=0}^{L_{y}^{\prime }}Y_{i,j-\ell^{\prime },p}\eta_{\ell^{\prime }qp}^{\beta }}{\sigma_{q}^{2}/\tau_{ijq}}+\text{log}\left(\frac{\left|I-B_{0}^{+}\right|}{\left|I-B_{0}^{-}\right|}\right)\right\}\right),$$

where $B_{0}^{+}$ and $B_{0}^{-}$ denote $B_{0}$ with $\xi_{qp}^{\beta }=1$ and $\xi_{qp}^{\beta }=-1$, respectively, and

$${\tilde{Y}}_{ijq}=Y_{ijq}-\mu_{q}-\sum_{\ell^{\prime }=0}^{L_{y}^{\prime }}\sum_{p^{\prime }\neq p}Y_{i,j-\ell^{\prime },p^{\prime }}\beta_{\ell^{\prime }qp^{\prime }}-\sum_{\ell^{\prime }=0}^{L_{x}^{\prime }}\sum_{s=1}^{S}X_{i,j-\ell^{\prime },s}\alpha_{\ell^{\prime }qs}.$$

The full conditional distribution for $\xi_{qs}^{\alpha }$ is similar, except it does not involve the additional term $\left|I-B_{0}^{+}\right|/\left|I-B_{0}^{-}\right|$. We can update $\xi_{qs}^{\alpha }$ analogously.

#### 1.2*. Update $\eta_{0qp}^{\beta }$

Since the full conditional distribution for $\eta_{0qp}^{\beta }$ involves the additional term $|I-B_{0}|$, there is no closed form solution. Therefore, we will update it using the Metropolis-Hasting algorithm. At each Metropolis-Hasting step, we will accept the move only if the maximum modulus of $B_{0}$’s eigenvalues is strictly less than 1, to ensure that the proposed model is well-defined.

#### 1.3*. Update $\eta_{\ell qp}^{\beta },\ell >0$ and $\eta_{\ell qs}^{\alpha },\ell \geq 0$

The full conditional distribution for $\eta_{\ell qp}^{\beta },\ell >0$ is

$$p\left(\eta_{\ell qp}^{\beta }|\cdot \right)\propto {𝒩}^{+}\left(0,\gamma_{\ell qp}^{\beta }\nu_{\ell qp}^{\beta }\right)\prod_{i}\prod_{j=\ell +1}^{J_{i}}𝒩\left(Y_{ijq}|Y_{ijq}^{*},\frac{\sigma_{q}^{2}}{\tau_{ijq}}\right)\propto {𝒩}^{+}\left(\mu_{\eta },\sigma_{\eta }^{2}\right),$$

where

$$\sigma_{\eta }^{2}={\left(\sum_{i}\sum_{j=\ell +1}^{J_{i}}\frac{{\left(Y_{i,j-\ell ,p}\xi_{qp}^{\beta }\right)}^{2}}{\sigma_{q}^{2}/\tau_{ijq}}+\frac{1}{\gamma_{\ell qp}^{\beta }\nu_{\ell qp}^{\beta }}\right)}^{-1},$$

$$\mu_{\eta }=\sigma_{\eta }^{2}\left(\sum_{i}\sum_{j=\ell +1}^{J_{i}}\frac{Y_{i,j-\ell ,p}\xi_{qp}^{\beta }}{\sigma_{q}^{2}/\tau_{ijq}}{\tilde{Y}}_{ijq}\right),$$

$${\tilde{Y}}_{ijq}=Y_{ijq}-\mu_{q}-\sum_{\ell^{\prime },p^{\prime }\neq \ell ,p}Y_{i,j-\ell^{\prime },p^{\prime }}\beta_{\ell^{\prime }qp^{\prime }}-\sum_{\ell^{\prime }=0}^{L_{x}^{\prime }}\sum_{s=1}^{S}X_{i,j-\ell^{\prime },s}\alpha_{\ell^{\prime }qs}.$$

We can update $\eta_{\ell qS}^{\alpha },\ell \geq 0$ analogously.
